# Halogen‐Bonding Heteroditopic [2]Catenanes for Recognition of Alkali Metal/Halide Ion Pairs

**DOI:** 10.1002/anie.202214785

**Published:** 2022-12-20

**Authors:** Hui Min Tay, Yuen Cheong Tse, Andrew Docker, Christian Gateley, Amber L. Thompson, Heike Kuhn, Zongyao Zhang, Paul D. Beer

**Affiliations:** ^1^ Department of Chemistry University of Oxford Chemistry Research Laboratory Mansfield Road Oxford OX1 3TA UK; ^2^ Department of Chemistry The University of Hong Kong Pokfulam Road Hong Kong P. R. China

**Keywords:** Halogen Bonding, Heteroditopic Receptors, Ion-Pair Recognition, Mechanically Interlocked Molecules, Solid–Liquid Extraction

## Abstract

The first examples of halogen bonding (XB) heteroditopic homo[2]catenanes were prepared by discrete Na^+^ template‐directed assembly of oligo(ethylene glycol) units derived from XB donor‐containing macrocycles and acyclic bis‐azide precursors, followed by a Cu^I^‐mediated azide‐alkyne cycloaddition macrocyclisation reaction. Extensive ^1^H NMR spectroscopic studies show the [2]catenane hosts exhibit positive cooperative ion‐pair recognition behaviour, wherein XB‐mediated halide recognition is enhanced by alkali metal cation pre‐complexation. Notably, subtle changes in the catenanes’ oligo(ethylene glycol) chain length dramatically alters their ion‐binding affinity, stoichiometry, complexation mode, and conformational dynamics. Solution‐phase and single‐crystal X‐ray diffraction studies provide evidence for competing host‐separated and direct‐contact ion‐pair binding modes. We further demonstrate the [2]catenanes are capable of extracting solid alkali‐metal halide salts into organic media.

## Graphical abstract

A series of halogen bonding heteroditopic [2]catenanes exhibit positive cooperative ion‐pair recognition behaviour, which has been exploited for the extraction of solid sodium or potassium halide salts into organic media. Changing the size of the component macrocycles dramatically influences the ion‐binding affinity, stoichiometry, and ion‐pair complexation mode of the [2]catenanes.

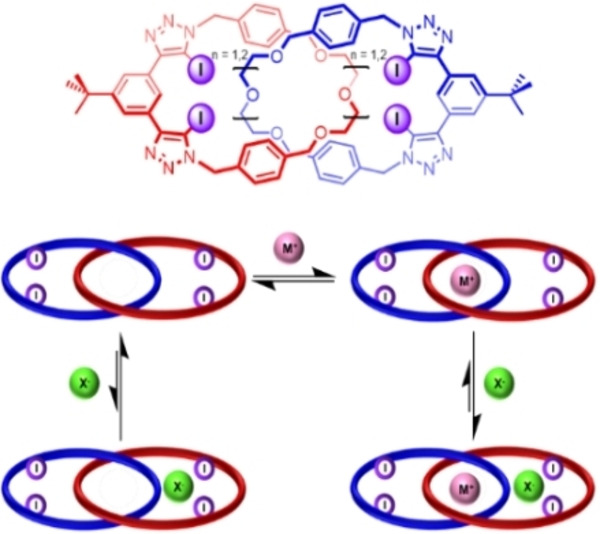

## Introduction

The strong and selective recognition of charged species remains a considerable challenge in supramolecular host–guest chemistry.[^1^, ^2^, ^3^, ^4^, ^5^, ^6^] In this context, heteroditopic receptors possessing both cation and anion binding sites typically offer advantages over monotopic analogues by exploiting the cooperative effects associated with ion‐pair binding.[^7^, ^8^, ^9^, ^10^, ^11^] The favourable affinity and selectivity profiles displayed by heteroditopic ion‐pair receptors have enabled their use in a myriad of applications, including salt extraction/solubilisation,[^12^, ^13^, ^14^, ^15^, ^16^, ^17^, ^18^, ^19^, ^20^, ^21^, ^22^] membrane transport[^23^, ^24^] and the recognition of biologically‐relevant zwitterions.[^25^, ^26^]

From a molecular recognition perspective, mechanically interlocked molecules (MIMs) present a unique opportunity to generate 3D binding sites which can be tailored to the specific geometric requirements of a target guest by functionalisation with appropriate recognition motifs.[^27^, ^28^, ^29^, ^30^, ^31^, ^32^, ^33^, ^34^] Inspired by nature's oxoanion‐selective protein designs, during the past two decades we have strategically constructed mechanically interlocked host structures (MIMs) for anion recognition.[^35^, ^36^, ^37^, ^38^, ^39^, ^40^] Recently the integration of halogen bonding (XB) donors to decorate acyclic, macrocyclic and MIM receptor binding sites has proven to be a promising alternative to traditionally employed hydrogen bonding (HB) interactions by virtue of the former's often superior anion binding strength, more stringent linear interaction geometries and contrasting selectivity profiles.[^41^, ^42^, ^43^, ^44^, ^45^, ^46^] Surprisingly, the use of interlocked structures for ion‐pair recognition remains remarkably rare, with the handful of reported receptors largely limited to [2]rotaxane topologies,[^47^, ^48^, ^49^, ^50^, ^51^, ^52^] and one example of a [2]catenane topology being used to this end.^[53]^

**Figure 1:**
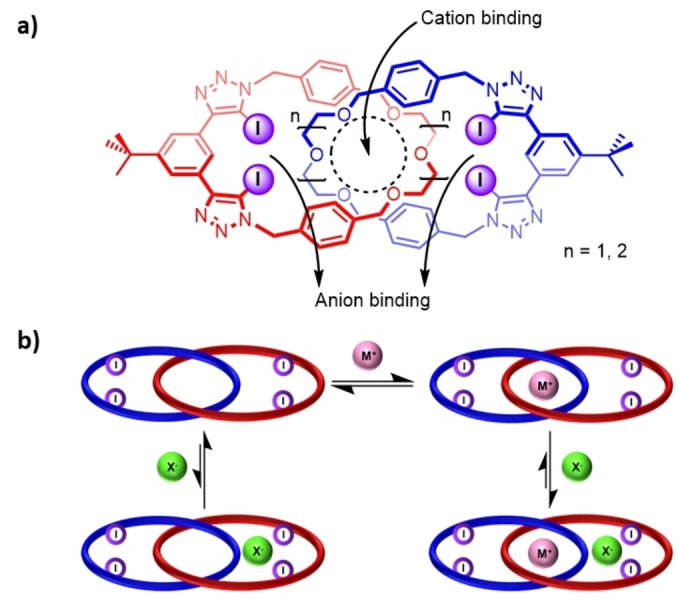
a) Chemical structures of the heteroditopic [2]catenane ion‐pair receptors studied in this work; b) Schematic showing ion‐pair recognition in the [2]catenane host systems.

Herein, we report the synthesis and ion‐pair binding properties of a series of XB heteroditopic [2]catenanes prepared by an alkali metal cation template‐directed approach (Figure 1), constituting the first report of neutral all‐XB [2]catenanes. Extensive quantitative ^1^H NMR titration studies with alkali metal cations, halide anions and their respective ion‐pairs reveal significant positive cooperativity between cation and anion binding events. Differences in the binding modes and stoichiometries of the ion‐pair bound [2]catenane complexes are probed by crystallographic analysis, revealing an unusual example of heteroditopic ion‐pair receptor systems wherein subtle changes in cavity size result in switching between either host‐separated or direct contact ion‐pair binding modes. The prodigious ion‐pair recognition properties of the [2]catenanes are also exploited to the end of developing solid–liquid extraction agents selective for alkali metal halide salts.

## Results and Discussion

**Scheme 1:**
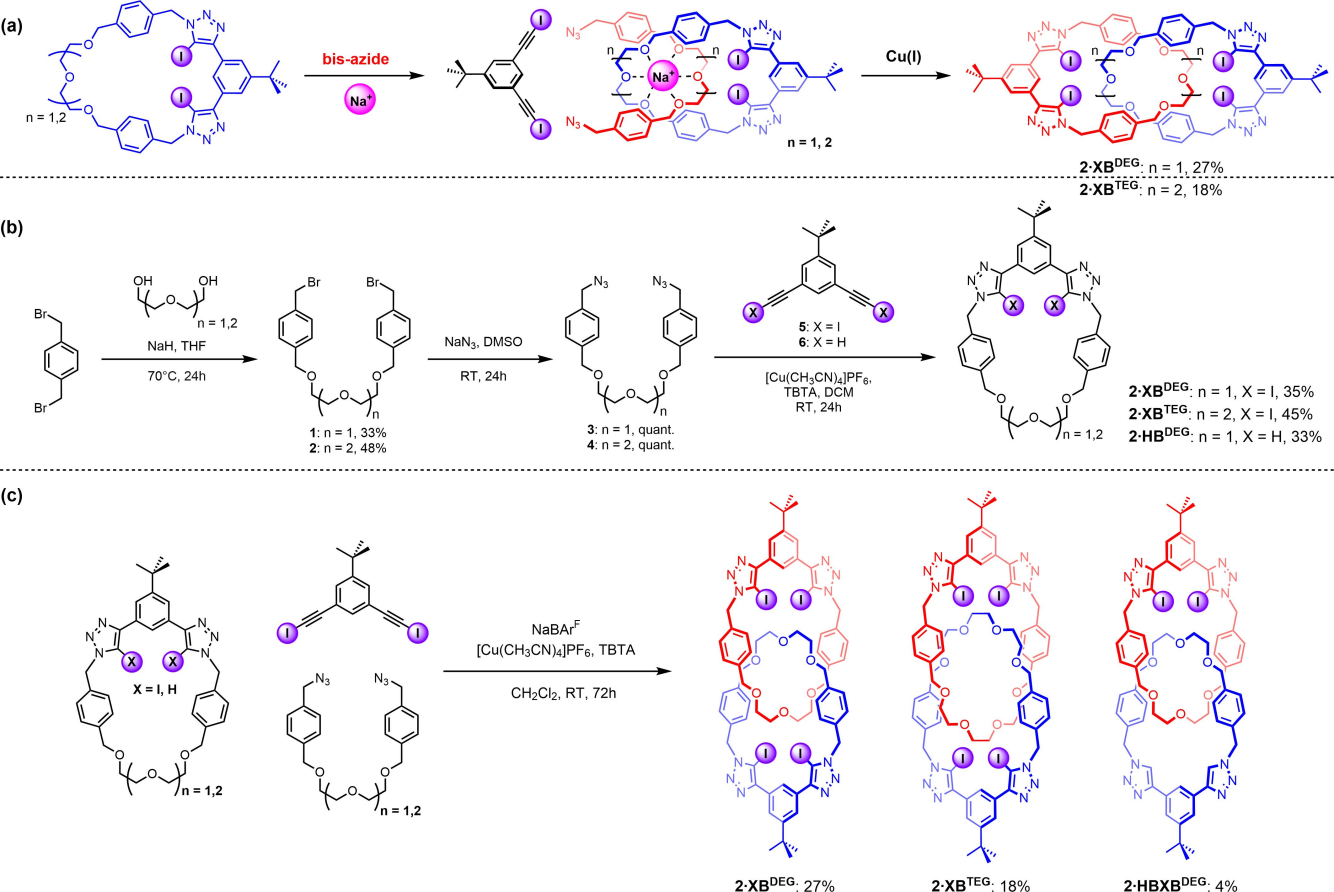
a) Alkali‐metal template‐directed approach for the synthesis of [2]catenanes. b) Synthesis of oligo(ethylene glycol)‐based macrocycles. c) Synthesis of heteroditopic [2]catenanes.

Inspired by Chiu's elegant use of alkali metal cation templates to direct the synthesis of functional interlocked topologies,[^54^, ^55^, ^56^, ^57^, ^58^, ^59^, ^60^] we sought to exploit this approach to construct XB heteroditopic [2]catenane host systems for ion‐pair binding. Conceivably, the target [2]catenanes could be prepared via a clipping methodology involving a sodium cation‐templated self‐assembly of a pseudo‐[2]rotaxane complex between an oligo(ethylene glycol)‐functionalised XB macrocycle and a bis‐azide, followed by a copper(I) catalysed alkyne‐azide cycloaddition (CuAAC)‐mediated cyclisation reaction with a bis‐iodoalkyne to form an additional bidentate XB donor motif (Scheme 1a). The mechanical bond formation step would thereby simultaneously generate both the cation and anion recognition sites.

### Synthesis and Characterisation

To prepare the requisite oligo(ethylene glycol)‐functionalised macrocycles **1⋅XB^DEG^** and **1⋅XB^TEG^**, di‐ and tri(ethylene glycol) were first alkylated with α,α′‐dibromo‐*p*‐xylene in the presence of NaH in THF, affording dibromides **1** and **2** in 33 % and 48 % respective yields after purification by column chromatography. Quantitative azidation was achieved by stirring the dibromides **1**‐**2** with excess NaN_3_ in DMSO, followed by aqueous work‐up to give bis‐azides **3**‐**4**. A high‐dilution CuAAC‐mediated macrocyclization reaction between **3**‐**4** and bis(iodo‐alkyne) **5** in the presence of [Cu(CH_3_CN)_4_]PF_6_ and TBTA in CH_2_Cl_2_ afforded XB macrocycles **1⋅XB^DEG^** and **1⋅XB^TEG^** in 35 % and 45 % yields respectively (Scheme 1b). A HB analogue macrocycle **1⋅HB^DEG^** was synthesised in 33 % yield via a similar reaction of **3** with bis(proto‐alkyne) **6**.

To confirm a sodium cation template could be used to assemble a pseudo‐[2]rotaxane topology, ^1^H NMR spectra of XB macrocycle **1⋅XB^DEG^** in the absence and presence of NaBAr^F^ and bis‐azide **3** were recorded (Figure S28). Addition of 1 equiv NaBAr^F^ to a solution of macrocycle **1⋅XB^DEG^** in CDCl_3_ led to notable perturbations in the proton resonances corresponding to H_g_, H_h_, H_i_, which are proximal to the di(ethylene glycol) region of **1⋅XB^DEG^**, indicating cation binding to the macrocycle. The subsequent addition of 1 equiv bis(azide) **3** to the mixture of **1⋅XB^DEG^** and NaBAr^F^ resulted in general upfield shifts of the proton resonances of **3**, with the largest changes (Δ*δ*>0.6 ppm) occurring at the di(ethylene glycol) protons. This is consistent with the formation of an interpenetrated complex of **1⋅XB^DEG^** and **3**, wherein the di(ethylene glycol) region of the bis‐azide thread experiences increased shielding from the ring current of the macrocycle xylene spacers,^[55]^ providing compelling evidence for the formation of the desired pseudo‐[2]rotaxane assembly. Similar indicative chemical shift perturbations were observed in the tri(ethylene glycol) proton resonances of TEG‐based bis(azide) **4** in the presence of TEG‐based XB macrocycle **1⋅XB^TEG^** and KBAr^F^ (Figure S29).

Encouraged by the evidence of pseudo‐[2]rotaxane formation, attention turned to the sodium cation template‐directed synthesis of the target [2]catenanes (Scheme 1c). In a typical reaction, an XB macrocycle and the corresponding bis‐azide were stirred in CH_2_Cl_2_ in the presence of one equivalent of NaBAr^F^ for 30 minutes at room temperature to facilitate assembly of the interpenetrated pseudo‐[2]rotaxane complex, to which bis(iodo‐alkyne) **5**, [Cu(CH_3_CN)_4_]PF_6_ and TBTA were added. The reaction mixture was allowed to stir at room temperature overnight. After aqueous work‐up with basic EDTA/NH_4_OH and purification by preparative TLC, [2]catenanes **2⋅XB^DEG^**, **2⋅XB^TEG^** and **2⋅HBXB^DEG^** were isolated in 27 %, 18 % and 4 % yields respectively and characterised by ^1^H NMR, ^13^C NMR, high‐resolution tandem mass spectrometry (Figure S11–27). Notably, the higher yield obtained for **2⋅XB^DEG^** relative to **2⋅XB^TEG^** is concordant with previous observations that Na^+^ is a more suitable template for di(ethylene glycol) chains.[^54^, ^61^] The lower yield of the hetero[2]catenane **2⋅HBXB^DEG^** was attributed to the poor solubility of the Na^+^‐complexed HB macrocycle **1⋅HB^DEG^** in CH_2_Cl_2_.

**Figure 2:**
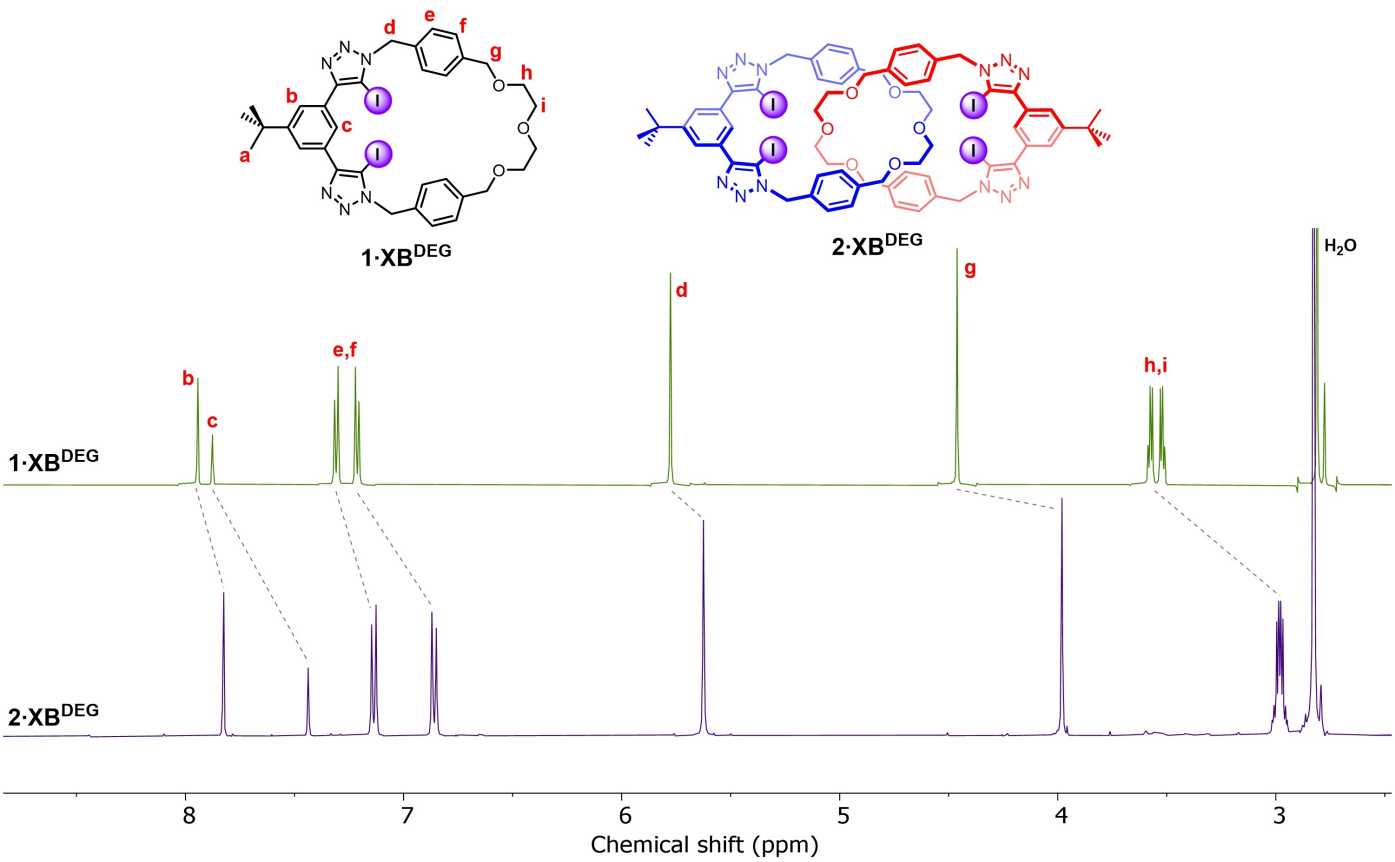
Stacked ^1^H NMR spectra of macrocycle **1⋅XB^DEG^** (top) and [2]catenane **2⋅XB^DEG^** (bottom) (500 MHz. acetone‐d_6_, 298 K).

Inspection of the ^1^H NMR spectra of the [2]catenanes with their respective parent macrocycles revealed diagnostic shifts consistent with an interlocked topology, namely a splitting of the aryl spacer signals and large upfield perturbations of the ethylene glycol signals (Figure 2, S19, S26), attributed to a shielding effect arising from the formation of the mechanical bond. Unequivocal evidence for the interlocked nature of the products was obtained via single crystal X‐ray diffraction analysis (Figure S77).

### Cation, Anion and Ion‐Pair Binding Studies

**Figure 3:**
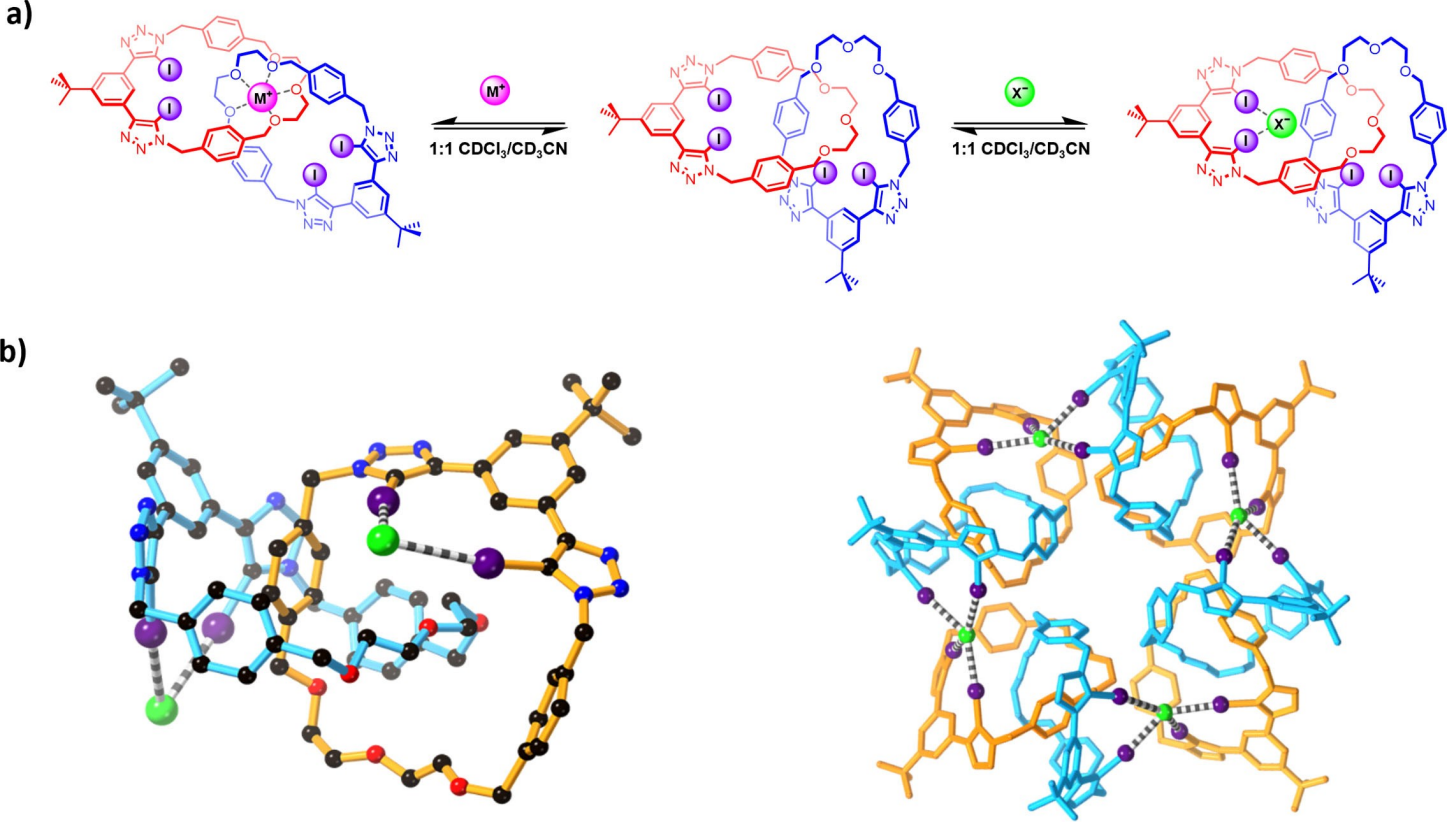
a) Proposed binding modes of [2]catenane **2⋅XB^DEG^** to alkali‐metal cations (left) and halide anions (right). b) Solid‐state structure of **2⋅XB^DEG^** ⋅TBACl, showing the XB interactions between a [2]catenane and two chloride anions (left), and the supramolecular tetramer comprising four [2]catenanes and chloride anions arranged around a crystallographic four‐fold axis (right). Hydrogen atoms and TBA^+^ counterions have been omitted for clarity. Where a ball‐and‐stick representation has been used, atom colours are as follows: black (carbon), blue (nitrogen), red (oxygen), purple (iodine), green (chlorine).

To firstly establish the cation binding properties of the homo[2]catenanes **2⋅XB^DEG^** and **2⋅XB^TEG^**, ^1^H NMR titration experiments were conducted by adding aliquots of NaBAr^F^ or KBAr^F^ to a 1 mM solution of the respective catenane in 1 : 1 CDCl_3_/CD_3_CN. Upon the addition of increasing equivalents of the cation, significant perturbations in the resonances corresponding to the ethylene glycol protons were observed, indicating that alkali metal cation complexation is occurring via the polyether oxygen atoms. Additional perturbations in proton resonances remote from the polyether signals, in particular upfield shifts of the internal benzene proton H_c_, were ascribed to co‐conformational changes occurring upon cation binding. Conceivably, cation binding might induce rearrangement and structural rigidification of the polyether regions to form a suitable binding cavity, as shown in Figure 3a.

The perturbations of multiple proton signals proximal to the cation binding site were monitored and the resulting titration isotherms were fitted to a global 1 : 1 host–guest stoichiometric binding model using Bindfit analysis^[62]^ to determine the cation association constants (Table 1). The two [2]catenanes exhibit reversed preferences for metal cations, with **2⋅XB^DEG^** having a >3‐fold preference for Na^+^ over K^+^, while **2⋅XB^TEG^** shows a >2‐fold preference for K^+^ over Na^+^, the origin of which is presumably size complementarity between the shorter di(ethylene glycol) linker and the smaller Na^+^ cation, as well as between the longer tri(ethylene glycol) linker and the larger K^+^ cation. The salient role of the mechanical bond for cation binding is reflected by similar binding studies conducted with the XB macrocycles **1⋅XB^DEG^** and **1⋅XB^TEG^** which determined no measurable *K*_a_ values.

**Table 1:** Cation association constants (*K*_a_/M^−1^) for macrocycles **1⋅XB^DEG^** and **1⋅XB^TEG^**, and [2]catenanes **2⋅XB^DEG^** and **2⋅XB^TEG^** determined by ^1^H NMR titrations.^[a]^

Cation	**1⋅XB^DEG^**	**1⋅XB^TEG^**	**2⋅XB^DEG^**	**2⋅XB^TEG^**
Na^+^	N.B.^[b]^	N.B.	756	753
K^+^	N.B.	N.B.	284	1734

[a] *K*_a_ values calculated using Bindfit with a 1 : 1 host–guest binding model. Errors are all <2 %. All cations added as BArF^−^ salts. Solvent=1 : 1 CDCl_3_/CD_3_CN. *T*=298 K. [Receptor]=1.0 mM. [b] N.B.=No binding.

The anion binding properties of the XB catenane and macrocycle receptors were also investigated by analogous titration experiments conducted with halides as their tetrabutylammonium (TBA^+^) salts also in 1 : 1 CDCl_3_/CD_3_CN solvent mixtures. With increasing anion concentration, the most significant perturbations were observed in the internal benzene proton H_c_ which shifted progressively downfield, indicating an anion binding event occurring via XB interactions in the bis(iodotriazole)benzene cleft (Figure 3a). Bindfit analysis of the resulting binding isotherms determined 1 : 1 host–guest stoichiometric halide anion association constants (Table 2). Interestingly, attempts to fit the anion binding isotherms of **2⋅XB^DEG^** and **2⋅XB^TEG^** to 1 : 2 host–guest binding models were unsuccessful, which is perhaps surprising given the [2]catenanes possess two bidentate XB binding motifs. However, the poor quality of fit obtained suggests that the second anion binding association constant is too small to be measureable (*K*_a_<10 M^−1^). To account for the presence of two degenerate anion binding sites in the [2]catenanes, the observed association constants of **2⋅XB^DEG^** and **2⋅XB^TEG^** were treated with a statistical factor of 2.^[63]^

**Table 2:** Anion association constants (*K*_a_/M^−1^) for macrocycles **1⋅XB^DEG^** and **1⋅XB^TEG^**, and [2]catenanes **2⋅XB^DEG^** and **2⋅XB^TEG^** determined by ^1^H NMR titrations.^[a]^

Anion	**1⋅XB^DEG^**	**1⋅XB^TEG^**	**2⋅XB^DEG^**	**2⋅XB^TEG^**
Cl^−^	125	65	66^[b]^	84^[b]^
Br^−^	154	97	78^[b]^	142^[b]^
I^−^	186	146	87^[b]^	135^[b]^

[a] *K*_a_ values calculated using Bindfit with a 1 : 1 host–guest binding model. Errors (±) are all <5 %. All anions added as TBA^+^ salts. Solvent=1 : 1 CDCl_3_/CD_3_CN. *T*=298 K. [Receptor]=1.0 mM. [b] The reported *K*_a_ values have been statistically corrected to determine the anion affinity of one bidentate XB donor motif, where *K*_a,reported_=*K*_a,observed_/2.

In general, the neutral XB macrocycles and [2]catenanes displayed relatively weak halide anion affinities in 1 : 1 CDCl_3_/CD_3_CN (*K*_a_<200 M^−1^). It is interesting to note that the statistically‐adjusted anion *K*_a_ values of **2⋅XB^DEG^** were significantly lower than the parent di(ethylene glycol)‐based macrocycle **1⋅XB^DEG^** for all three halides. This suggests that the mechanical bond reduces the anion binding potency of each bis(iodotriazole)benzene XB donor motif, presumably due to steric effects reducing access to the XB binding site. This is supported by the anion affinities obtained for the larger catenane **2⋅XB^TEG^** being comparable to that of the parent macrocycle **1⋅XB^TEG^**.

Evidence for the proposed chloride binding mode of **2⋅XB^DEG^** was provided by solid state structural analysis.^[64]^ Crystals suitable for analysis by single crystal X‐ray diffraction were obtained via diffusion of diethyl ether into a 1 : 1 CDCl_3_/CD_3_CN solution of **2⋅XB^DEG^** and excess TBACl. The solid‐state structure revealed a 1 : 1 complex of [2]catenane **2⋅XB^DEG^** and TBACl stabilised by a network of intercomponent XB interactions. Each [2]catenane binds two chloride ions via XB interactions between the anion and the two independent bis(iodotriazole)benzene motifs on the interlocked macrocycles, as shown by the geometry as well as the I⋅⋅⋅Cl^−^ distances (3.156(4)–3.344(4) Å, which is 85–90 % of the sum of the van der Waal radii of the two atoms) (Figure 3b). Each chloride is also bound by two pairs of XB interactions, with the second pair arising from the bis(iodotriazole)benzene motif of a [2]catenane in a neighbouring asymmetric unit, giving rise to an overall 1 : 1 binding stoichiometry.

In line with the weak anion association constants of **2⋅XB^DEG^** determined via ^1^H NMR binding studies, the chloride anions bind to the periphery of each macrocycle. It would appear that an endotopic anion binding mode wherein the two bis‐iodotriazole motifs cooperatively bind a single anion in the interlocked cavity is not sterically feasible. Instead, the [2]catenane and chloride anions act as two‐connecting linkers and self‐assemble to form a supramolecular tetramer stabilised by intercomponent halogen bonding (Figure 3b). Also notable is the significant positional disorder in the di(ethylene glycol) regions of the macrocycles, which, in the absence of an alkali metal guest, exhibit considerable conformational flexibility.

**Figure 4:**
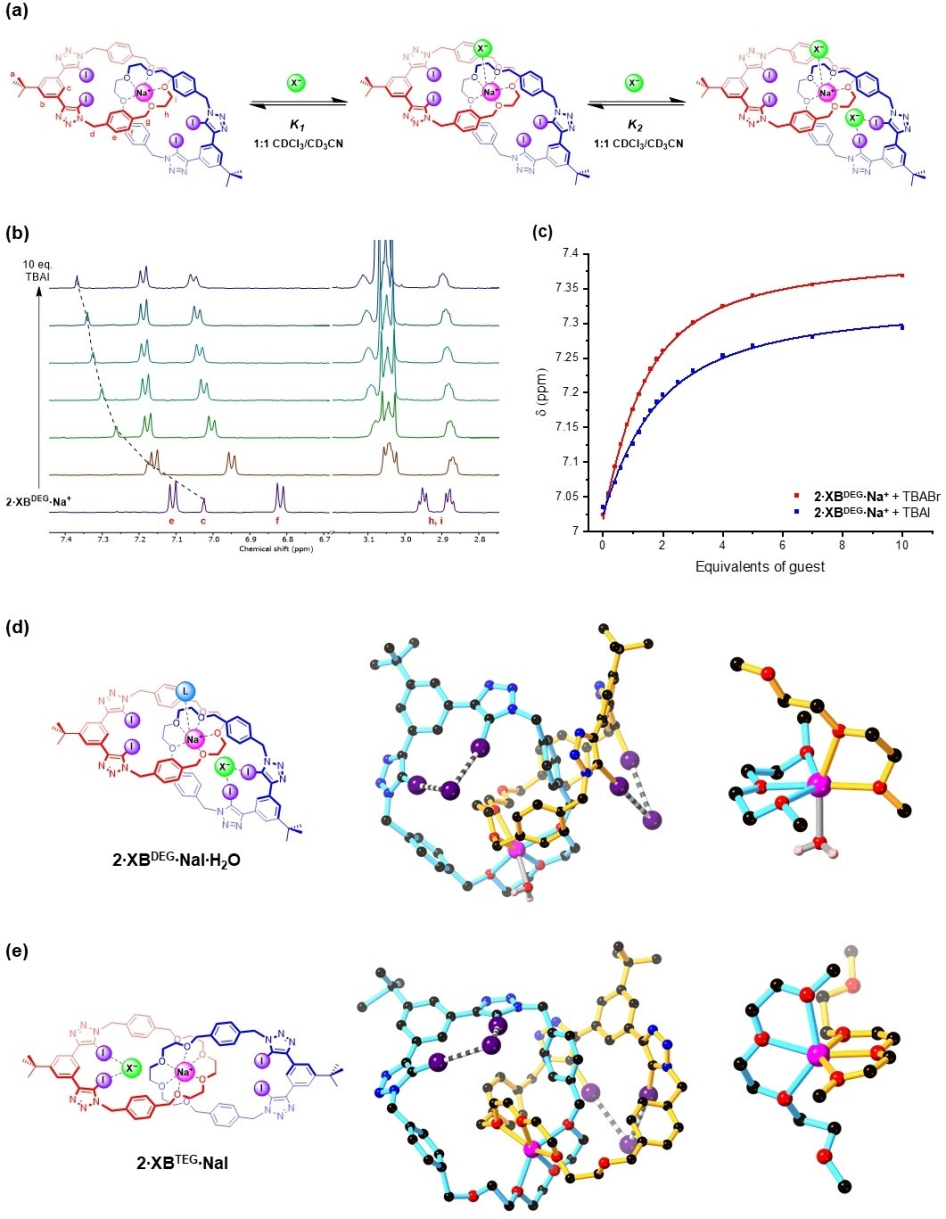
a) ^1^H NMR titration experiments of TBABr and **2⋅XB^DEG^** in the presence of 1 equiv NaBAr^F^ (500 MHz, 1 : 1 CD_3_CN/CDCl_3_, 298 K). b) Binding isotherms of **2⋅XB^DEG^⋅Na^+^** with TBABr (red) and TBAI (blue) generated by monitoring the chemical shifts of the internal benzene proton Hc, both fitted to the 1 : 2 (Full) host–guest binding model. Solid‐state structures of c) **2⋅XB^DEG^⋅NaI⋅H_2_O** and d) **2⋅XB^TEG^⋅NaI**, showing a single [2]catenane unit bound to a sodium cation and two iodide anions (middle) and the ethylene glycol region (right) highlighting the coordination environment around the sodium ion. Atom colours are as follows: black (carbon), blue (nitrogen), red (oxygen), purple (iodine), light pink (hydrogen), bright pink (sodium). The chemical structures of **2⋅XB^DEG^** and **2⋅XB^TEG^** are shown on the left for reference. e) Proposed ion‐pair binding equilibrium of **2⋅XB^DEG^**.

Having determined both the cation and anion binding behaviour of the neutral XB [2]catenanes, their ability to recognise alkali metal halides as ion‐pairs was investigated. This was undertaken by adding aliquots of TBA halide salts to a 1 mM solution of the [2]catenane in the presence of 1 equivalent M^I^BAr^F^ (M^I^=Na or K) **(**Figure 4a **)**. Under these conditions, only a fraction of the [2]catenane molecules in solution are initially complexed to the cationic guest, hence there remains at equilibrium a significant proportion of both free [2]catenane and cation. It is therefore important to consider the proportion of metal‐complexed [2]catenane present in each ion‐pair titration, which were calculated from the respective cation association constants and are shown in Table 3. Upon addition of Br^−^ and I^−^, downfield shifts in the internal benzene proton H_c_ were seen in both [2]catenanes alongside perturbations of the signals at the ethylene glycol region, suggesting that binding of the anion simultaneously enhances cation binding to the [2]catenane. In contrast, addition of Cl^−^ led to salt recombination and precipitation of NaCl or KCl, reflecting the high lattice energy of the alkali metal chloride salts.

**Table 3:** Apparent anion association constants (*K*_a_/M^−1^) for **2⋅XB^DEG^** and **2⋅XB^TEG^** in the presence of 1 equiv M^I^BAr^F^ (M^I^=Na, K) determined by ^1^H NMR titrations.^[a]^

Host	Cation	% bound	Anion	Binding model	*K*_1_	*K*_2_
**2⋅XB^DEG^**	Na^+^	34	Br^−^	1 : 2 (Full)	1900	435
			I^−^	1 : 2 (Full)	3080	787
	K^+^	18	Br^−^	1 : 1	216^[b]^	–
			I^−^	1 : 1	244^[b]^	–
**2⋅XB^TEG^**	Na^+^	33	Br^−^	1 : 1	373^[b]^	–
			I^−^	1 : 1	409^[b]^	–
	K^+^	47	Br^−^	1 : 1	492^[b]^	–
			I^−^	1 : 1	525^[b]^	–

[a] *K*_a_ values calculated using Bindfit with a 1 : 1 host–guest binding model. Errors (±) are all <5%. All cations added as BAr^F−^ salts. Solvent=1 : 1 CDCl_3_/CD_3_CN. *T*=298 K. [Receptor]=1.0 mM. [b] The reported *K*_a_ values have been statistically corrected to determine the anion affinity of one bidentate XB donor motif, where *K*_a,reported_=*K*_a,observed_/2.

Since metal complexation to the [2]catenane produces a monocationic species, there is a possibility of 1 : 2 host–guest stoichiometric binding that utilises both the anion binding sites. As such, all binding isotherms were fitted to both the 1 : 1 host–guest binding model, as well as four variants of the 1 : 2 host–guest binding model **(**Figure 4b **)**. Following an approach developed by Thordarson and co‐workers,^[65]^ the co‐variance values obtained from fitting the binding isotherms to each model were compared to determine the most appropriate binding model (details in Supporting Information). The binding models and corresponding *K_1_* and *K_2_* values obtained from this analysis are shown in Table 3.

Despite the introduction of a positive charge to the receptors, the apparent binding stoichiometry of **2⋅XB^DEG^⋅K^+^**, **2⋅XB^TEG^⋅Na^+^** and **2⋅XB^TEG^⋅K** ^+^ to both halide anions remains 1 : 1. Notable enhancements in the apparent *K*_1_ values were observed for both halides (Table 3). This was attributed to cooperative electrostatic effects commonly observed in ion‐pair receptors. All three [2]catenane‐metal complexes exhibited moderately enhanced binding to iodide over bromide. **2⋅XB^TEG^⋅K** ^+^ exhibited the strongest anion binding to both bromide and iodide, followed by **2⋅XB^TEG^⋅Na** ^+^, while **2⋅XB^DEG^⋅K** ^+^ showed the weakest binding. This trend was attributed to a combination of the intrinsically higher anion binding strength of **2⋅XB^TEG^** compared to **2⋅XB^DEG^** in the metal‐free neutral state (Table 2), as well as the differing proportions of metal‐bound complex initially present in solution (47 % for **2⋅XB^TEG^⋅K^+^**, 33 % for **2⋅XB^TEG^⋅Na^+^** and 17 % for **2⋅XB^DEG^⋅K^+^**).

In contrast, **2⋅XB^DEG^⋅Na^+^** exhibited a 1 : 2 host–guest binding stoichiometry with Br^−^ and I^−^. The binding isotherms of the two anions fit to the 1 : 2 (Full) model, which assumes there are two distinct anion binding sites in the receptor and that changes in chemical shift (Δ*δ*) corresponding to the 1 : 1 and 1 : 2 complexes are unrelated. The differences in binding stoichiometry between **2⋅XB^DEG^⋅Na^+^** and the other three metal‐[2]catenane complexes were initially difficult to rationalise as all the receptors contain a bound alkali metal cation and two XB anion binding sites. Analysis of the ion‐pair binding modes of the [2]catenanes in the solid state offered a possible explanation for this behaviour.

Vapour diffusion of pentane into a 1 : 1 CHCl_3_/CH_3_CN solution of **2⋅XB^DEG^** in the presence of 1 equiv NaBAr^F^ and excess TBAI resulted in the formation of colourless needle‐shaped single crystals suitable for X‐ray diffraction studies. The asymmetric unit contains one **2⋅XB^DEG^** catenane, a sodium cation, an iodide anion as well as a sodium‐bound water molecule, giving an overall chemical composition of **2⋅XB^DEG^⋅NaI⋅H_2_O**. In sharp contrast to the structure of **2⋅XB^DEG^⋅TBACl**, the di(ethylene glycol) chains of the two macrocycles are arranged orthogonally to form a binding pocket in which a sodium cation resides (Figure 4c). This observed inter‐ring circumrotation of the [2]catenane in the presence of a sodium cation is consistent with the postulated cation‐induced conformational change in solution, as suggested by the chemical shift perturbations of proton signals distal to the cation binding site in ^1^H NMR. Importantly, the cation forms coordination interactions with five di(ethylene glycol) oxygen atoms from the interlocked macrocycles and a sixth with a Na^+^‐ligated water molecule.

The anion binding mode in **2⋅XB^DEG^⋅NaI⋅H_2_O** is similar to **2⋅XB^DEG^⋅TBACl**, where each [2]catenane binds two iodide ions via the bidentate XB donor motifs of the two interlocked macrocycles (I⋅⋅⋅I^−^: 3.4592(19)–3.6524(19)Å, 87–92 % Σ_vdW_), while each iodide ion bridges two adjacent [2]catenanes **(**Figure 4c **)**. Unlike **2⋅XB^DEG^⋅TBACl**, however, this network of intercomponent XB interactions is propagated throughout the crystal, linking the [2]catenane units into 1D polymeric chains (Figure S78).

When **2⋅XB^TEG^** was crystallised under similar conditions, needle‐like crystals of chemical composition **2⋅XB^TEG^⋅NaI** formed. Similar to **2⋅XB^DEG^⋅NaI⋅H_2_O**, sodium ions occupy the binding site formed by the tri(ethylene glycol) regions of the interlocked macrocycles and each [2]catenane unit binds two iodide anions via XB interactions (I⋅⋅⋅I^−^: 3.4914(9)–3.709(6) Å, 88–94 % Σ_vdW_) **(**Figure 4d **)** to form XB‐stabilised polymeric chains (Figure S79). However, a key difference in the two structures lies in the coordination environment of the sodium ion; in **2⋅XB^TEG^⋅NaI**, all six sodium coordination sites are occupied by polyether oxygens, presumably due to the presence of additional oxygen atoms in the longer tri(ethylene glycol) linkers. Consequently, this leaves no vacant coordination site to accommodate an external ligand such as water or an additional halide.

Comparing the sodium cation coordination modes in the two structures suggests that the 1 : 2 stoichiometry of anion binding in **2⋅XB^DEG^⋅Na^+^** observed by solution‐state ^1^H NMR arises from a contact ion‐pair between a coordinatively unsaturated Na^+^ and the halide in addition to XB‐mediated anion binding **(**Figure 4e **)**. Importantly, this rationalises the higher apparent *K*_1_ values of **2⋅XB^DEG^⋅Na^+^** for the two anions (*K*_1_(Br^−^)=1900 M^−1^, *K*_1_(I^−^)=3080 M^−1^) compared to the other three metal‐[2]catenane complexes. As before, the stronger iodide binding is likely due to the lower lattice enthalpy of NaI making the competing salt recombination equilibrium less favourable.

Whilst it may be somewhat counterintuitive that **2⋅XB^DEG^⋅K^+^** did not exhibit the same 1 : 2 binding stoichiometry as **2⋅XB^DEG^⋅Na^+^**, given the larger ionic radius of potassium, it is important to note that this might be due to the low proportion of potassium‐bound [2]catenane complex present in a 1 mM solution of **2⋅XB^DEG^** and KBAr^F^, meaning that the binding isotherm derived from the titration is in reality a superposition of anion binding to neutral **2⋅XB^DEG^** (major, 83 %) and **2⋅XB^DEG^⋅K^+^** (minor, 17 %).

### Binding properties of mixed HB/XB hetero[2]catenane 2⋅HBXB^DEG^

**Figure 5:**
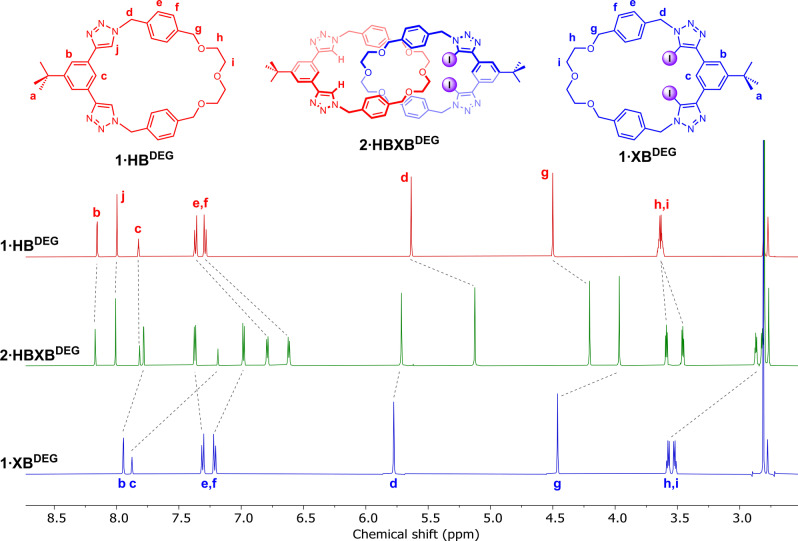
Stacked ^1^H NMR spectra of **1⋅HB^DEG^** (top), **2⋅HBXB^DEG^** (middle) and **1⋅XB^DEG^** (bottom) (acetone‐*d_6_*, 500 MHz, 298 K).

To evaluate the contribution of XB interactions to the ion‐pair recognition properties of the [2]catenane receptors, the hetero[2]catenane **2⋅HBXB^DEG^** was subsequently prepared (Scheme 1c), wherein one macrocycle features a bis(iodotriazole) XB donor motif and the other a bis(prototriazole) HB donor motif. A comparison of the ^1^H NMR spectrum of **2⋅HBXB^DEG^** with that of the parent macrocycles revealed chemical shift perturbations indicative of mechanical bond formation (Figure 5). Analysis of **2⋅HBXB^DEG^** by 2D ^1^H‐^1^H ROESY NMR enabled a complete assignment of the proton resonances (Figure S24).

**Figure 6:**
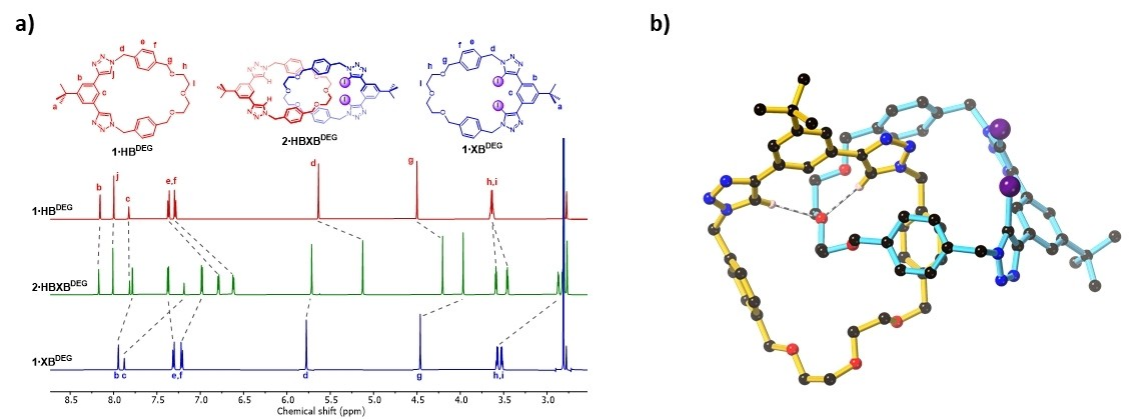
Solid‐state structure of **2⋅HBXB^DEG^**, showing the HB interactions between H_j_ and a polyether oxygen atom. All other hydrogen atoms have been omitted for clarity. Atom colours are as follows: black (carbon), blue (nitrogen), red (oxygen), purple (iodine), pink (hydrogen).

Crystals suitable for X‐ray diffraction studies were obtained by slow evaporation of a cooled acetone solution of **2⋅HBXB^DEG^**. The structure confirmed the interlocked [2]catenane topology of **2⋅HBXB^DEG^** (Figure 6). Notably, the crystal structure features intercomponent CH⋅⋅⋅O hydrogen bonding interactions between the triazole donor atoms *H_j_* and a polyether oxygen on the opposite ring (C⋅⋅⋅O distances: 3.333(8)–3.398(8) Å). As a result, the di(ethylene glycol) chains are not oriented in the optimal conformation to facilitate sodium cation complexation.

The binding affinities of **2⋅HBXB^DEG^** for NaBAr^F^ and TBAI were determined in 1 : 1 CDCl_3_/CD_3_CN (Supporting Information Figures S63‐64, 69). Fitting the resulting isotherms to a 1 : 1 host–guest stoichiometric binding model in Bindfit gave *K*_a_ values of 36 M^−1^ for Na^+^ and 31 M^−1^ for I^−^. The drastically reduced sodium cation affinity of **2⋅HBXB^DEG^** relative to **2⋅XB^DEG^** (*K*_a_=756 M^−1^) was attributed to the reduced basicity of the polyether oxygens involved in intercomponent HB interactions, as seen in the crystal structure of **2⋅HBXB^DEG^**. The low iodide affinity of **2⋅HBXB^DEG^** likely results from hindered access to the single bis(iodotriazole) anion binding cleft upon mechanical bond formation. Notably, the HB macrocycle **1⋅HB^DEG^** exhibits no binding to I^−^ in this solvent system. The weak sodium binding of **2⋅HBXB^DEG^** precluded a determination of an apparent iodide binding constant of **2⋅HBXB^DEG^⋅Na^+^** due to the low proportion (3 %) of sodium‐complexed receptor present in an equimolar mixture of **2⋅HBXB^DEG^** and NaBAr^F^.

### Solid‐liquid extraction studies

Encouraged by the ion‐pair recognition capabilities of homo[2]catenanes **2⋅XB^DEG^** and **2⋅XB^TEG^**, the ability of these XB [2]catenanes to act as alkali metal halide salt extractants was investigated. Solid‐liquid extraction (SLE) studies were conducted by sonicating a 1 mM CDCl_3_ solution of the [2]catenane in the presence of excess solid NaX for **2⋅XB^DEG^** or KX for **2⋅XB^TEG^** (X=Cl^−^, Br^−^, I^−^). After 30 minutes, the solution was filtered and a ^1^H NMR spectrum recorded.

**Figure 7:**
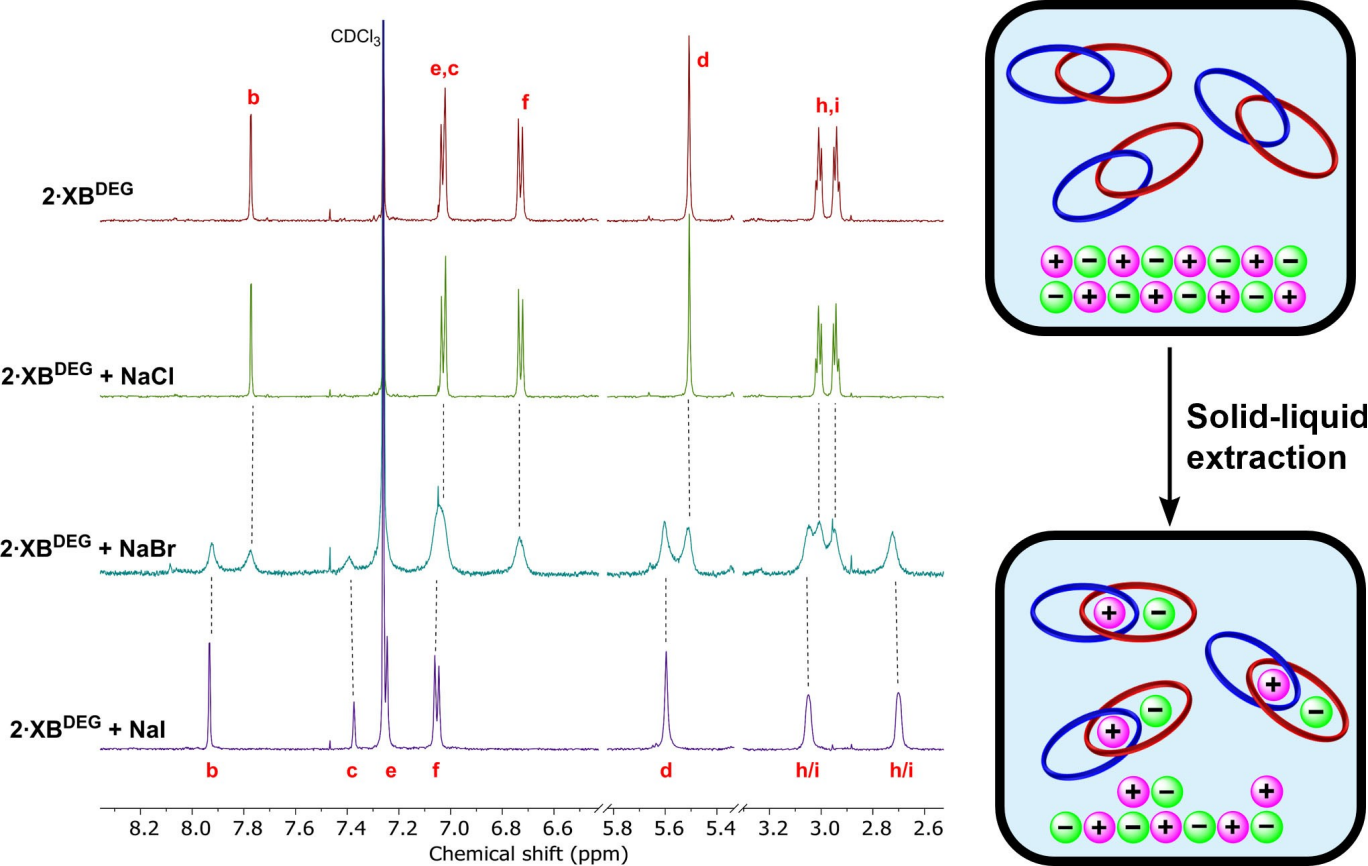
Pre‐ and post‐extraction spectra of **2⋅XB^DEG^** with excess solid NaCl, NaBr, and NaI (CDCl_3_, 500 MHz, 298 K).

The pre‐ and post‐extraction ^1^H NMR spectra of **2⋅XB^DEG^** showed perturbations consistent with those observed in the ion‐pair titrations of **2⋅XB^DEG^** with NaBAr^F^ and TBAI, namely a divergence of the ethylene glycol protons H_h_ and H_i_, as well as a downfield shift of internal benzene proton H_c_ (Figure 7), indicative of successful extraction of NaI by the receptor. Notably, the subsequent addition of free [2]catenane **2⋅XB^DEG^** to the solution gave rise to a second set of peaks corresponding to free **2⋅XB^DEG^** (Figure S70), suggesting that the system is in slow exchange between the fully NaI‐complexed and free receptors. An analogous study using NaBr resulted in two sets of broad peaks, one corresponding to unbound **2⋅XB^DEG^** while the other displays similar chemical shifts to that of NaI‐bound **2⋅XB^DEG^**, indicating partial extraction of NaBr by the [2]catenane. Integration of these respective signals estimated that 50 % of the [2]catenane in solution was present as the NaBr‐bound complex. In contrast, no extraction of NaCl occurred, which was attributed to the higher lattice enthalpy of NaCl.

Treating **2⋅XB^TEG^** with potassium halide salts in analogous SLE experiments gave rise to a single set of peaks in all cases, indicative of systems in fast exchange (Figure S74). The extraction efficiencies were estimated from the magnitude of the observed chemical shift perturbations and found to be in the order: KCl≪KBr<KI. Importantly, the parent macrocycles **1⋅XB^DEG^** and **1⋅XB^TEG^** were subjected to the same SLE conditions with NaX and KX but no evidence of salt extraction was observed, adding further testimony to the critical role of mechanical bond formation in enhancing the ion‐pair recognition capabilities of the receptors.

## Conclusion

In summary, an alkali metal template‐directed approach was applied to the preparation of a series of unprecedented neutral XB heteroditopic [2]catenanes, comprising oligo(ethylene glycol) motifs for alkali metal cation binding and XB donor motifs for anion recognition. Extensive ^1^H NMR binding studies indicate a drastic turn‐on of cation affinity in the interlocked hosts relative to the parent macrocycles, while solid‐state structural analysis provides evidence for cation‐induced conformational dynamism. Importantly, the weak halide anion affinities of the neutral XB [2]catenanes were significantly augmented by pre‐complexation of an alkali metal cation to the receptors, demonstrating cooperative electrostatic effects that make ion‐pair receptors so desirable in the context of supramolecular host–guest recognition. An unexpected switch from a 1 : 1 to 1 : 2 host‐anion binding stoichiometry in **2⋅XB^DEG^⋅Na^+^** was attributed to the formation of an additional contact ion‐pair between the anion and the coordinatively‐unsaturated metal centre. This constitutes an unusual example of an ion‐pair receptor in which the binding stoichiometry of the anion can be indirectly modulated by changing the size of the cation binding site, which may be valuable in informing the considered design of future multicomponent interlocked receptors for ion‐pairs. The potential capability of the XB [2]catenane receptors to function as extractants of alkali halide salts was demonstrated via a series of solid–liquid extraction studies, highlighting the myriad of exciting possibilities associated with exploiting the mechanical bond for the development of novel receptors for ion‐pair recognition and extraction.

## Conflict of interest

The authors declare no conflict of interest.

1

## Supporting information

As a service to our authors and readers, this journal provides supporting information supplied by the authors. Such materials are peer reviewed and may be re‐organized for online delivery, but are not copy‐edited or typeset. Technical support issues arising from supporting information (other than missing files) should be addressed to the authors.

Supporting Information

Supporting Information

Supporting Information

## Data Availability

The data that support the findings of this study are available in the Supporting Information of this article.
