# Supplementary material for: Halogen‐Bonding Heteroditopic [2]Catenanes for Recognition of Alkali Metal/Halide Ion Pairs
Source: Angew Chem Int Ed Engl. 2022 Dec 20;62(5):e202214785. doi: 10.1002/anie.202214785 (PMC10108176; doi:10.1002/anie.202214785)
Supplement: Supplementary file 1 — Supporting Information [file ANIE-62-0-s003.pdf]

Angewandte Chemie
Eine Zeitschrift der Gesellschaft Deutscher Chemiker

Supporting Information

Halogen-Bonding Heteroditopic [2]Catenanes for Recognition of Alkali Metal/Halide Ion Pairs

*H. M. Tay, Y. C. Tse, A. Docker, C. Gateley, A. L. Thompson, H. Kuhn, Z. Zhang, P. D. Beer**

Table of Contents

Materials and Methods 3

Synthesis and characterisation 4

^{1}H NMR pseudorotaxane studies 29

^{1}H NMR binding studies 31

Solid-liquid extraction experiments 54

Crystal structure determination 58

Materials and Methods

All solvents and reagents were purchased from commercial suppliers and used as received unless otherwise stated. Dry solvents were obtained by purging with nitrogen and then passing through an MBraun MPSP-800 column. H2O was de-ionized and micro filtered using a Milli-Q ® Millipore machine. Column chromatography was carried out on Merck® silica gel 60 under a positive pressure of nitrogen. Routine NMR spectra were recorded on either a Varian Mercury 300, a Bruker AVIII 400 or a Bruker AVIII 500 spectrometer with ^{1}H NMR titrations recorded on a Bruker AVIII 500 spectrometer. TBA salts were stored in a vacuum desiccator containing phosphorus pentoxide prior to use. Where mixtures of solvents were used, ratios are reported by volume. Chemical shifts are quoted in parts per million relative to the residual solvent peak. Mass spectra were recorded on a Bruker µTOF spectrometer. Triethylamine was distilled from and stored over potassium hydroxide. Tris[(1-benzyl-1H-1,2,3- triazol-4-yl)methyl]amine (TBTA),[1] di- and triethylene glycol-based dibromides (**1-2**),[2] 1-(tert-butyl)-3,5-bis(iodoethynyl)benzene (**5**) and 1-(tert-butyl)-3,5-diethynylbenzene (**6**)[3] were prepared according to previous literature reports.

Synthesis and characterisation

General procedure 1: Conversion of dibromide precursors to bis-azides

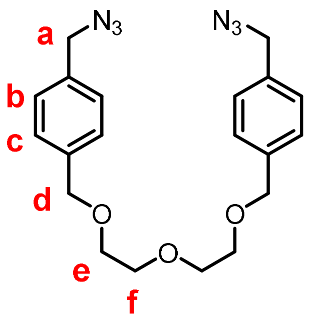

Diethylene glycol-based bis-azide (3). Dibromide **1** (1.38 g, 2.92 mmol) was dissolved in DMSO (18 mL). Sodium azide (0.76 g, 11.69 mmol) was added and the reaction mixture was stirred at room temperature overnight. The crude reaction mixture was diluted with H_2O (50 mL) and extracted with diethyl ether (30 mL × 3). The combined organic layer was washed with brine (30 mL × 3), dried over anhydrous $MgSO_4$, filtered and concentrated in vacuo to afford **3** as a yellow oil (1.16 g, quant.).

^{1}H NMR (500 MHz, $CDCl_3$) δ (ppm) 7.40 – 7.34 (m, $4H_b$), 7.30 – 7.27 (m, $4H_c$), 4.58 (s, $4H_a$), 4.32 (s, $4H_d$), 3.75 – 3.60 (m, $8H_{e,f}$).

^{13}C NMR (126 MHz, $CDCl_3$) δ (ppm) 138.70, 134.78, 128.43, 128.27, 72.98, 70.86, 69.76, 54.69.

HRMS (ESI +ve) m/z: 419.1793 ($[M+Na]^+$, $C_{20}H_{24}O_3N_6Na$ requires 419.1802).

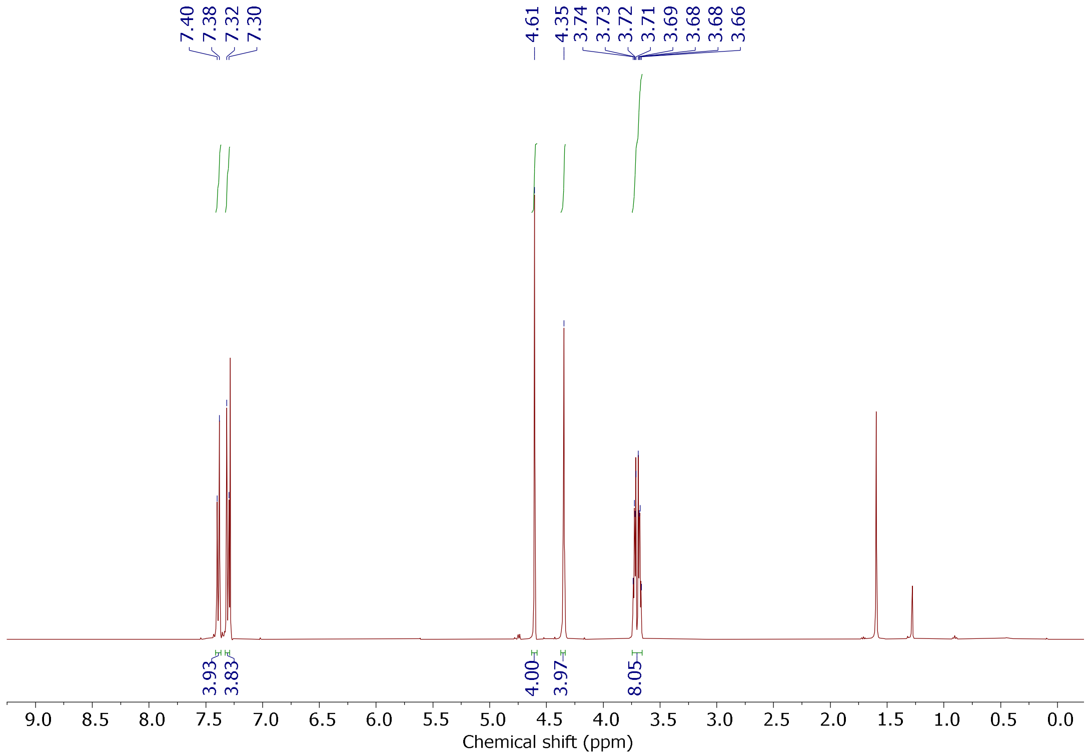

*Figure S1. ^{1}H-NMR spectrum of DEG-bis(azide) **3** (500 MHz, $CDCl_3$, 298 K)*

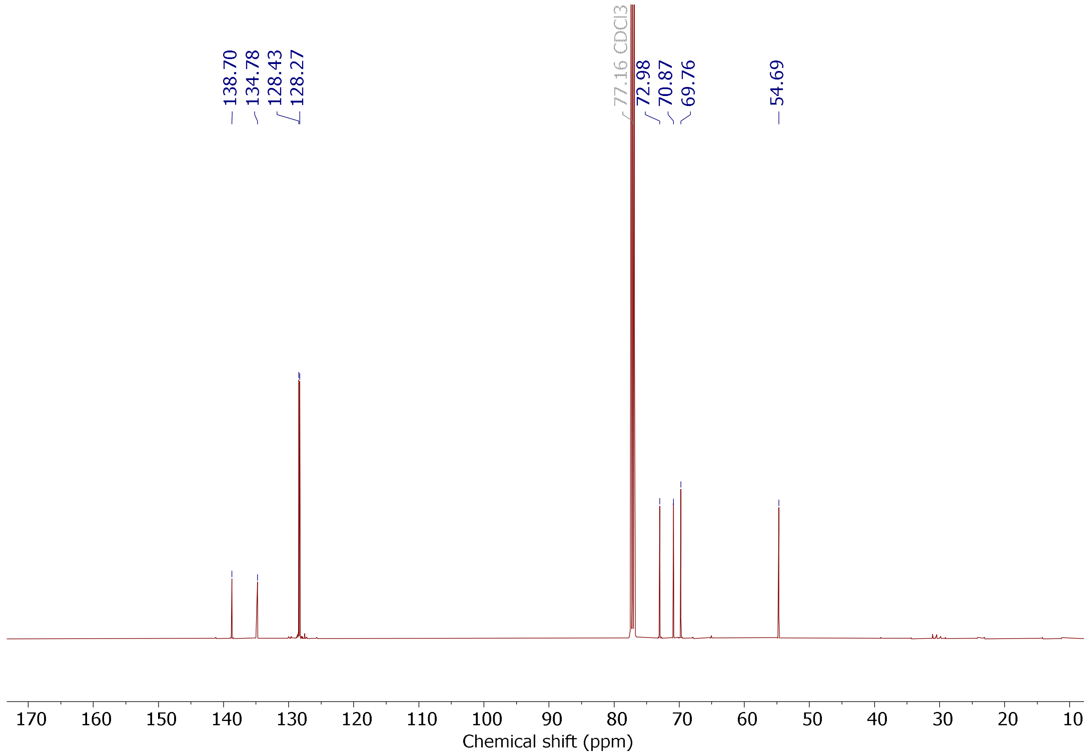

*Figure S2. ^{13}C-NMR spectrum of DEG-bis(azide) **3** (126 MHz, $CDCl_3$, 298 K)*

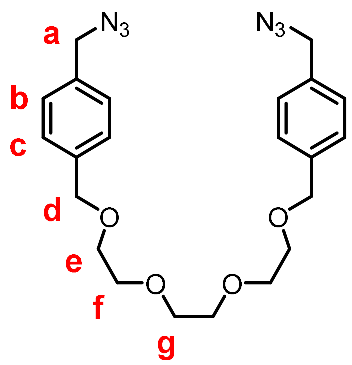

Triethylene glycol-based bis-azide (4). Following general procedure 1, the reaction of dibromide **2** (2.59 g, 5.01 mmol) and sodium azide (1.30 g, 20.1 mmol) yielded the desired product as a yellow oil (2.19 g, quant).

^{1}H NMR (500 MHz, acetone-*d*$_6$) δ (ppm) 7.38 (m, *J* = 8.3 Hz, 8$H_{b,c}$), 4.56 (s, 4H_a), 4.42 (s, 4H_d), 3.69 – 3.57 (m, 12$H_{e,f,g}$).

^{13}C NMR (126 MHz, acetone-*d*$_6$) δ (ppm) 140.08, 135.75, 129.21, 128.66, 73.05, 71.35, 71.29, 70.58, 54.84.

HRMS (ESI +ve) m/z: 463.2057 ([M+Na]$^+$, $C_{22}H_{28}O_3N_6Na$ requires 463.2064).

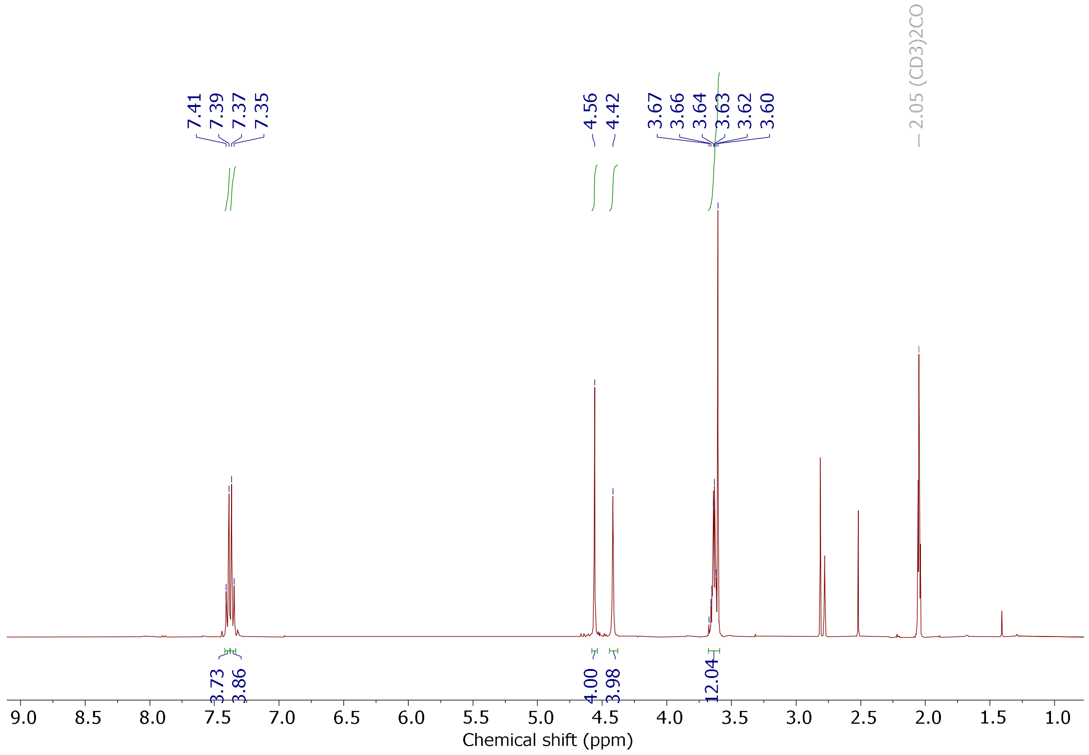

Figure S3. ^{1}H-NMR spectrum of TEG-bis(azide) ***4*** *(500 MHz, acetone-d_6, 298 K)*

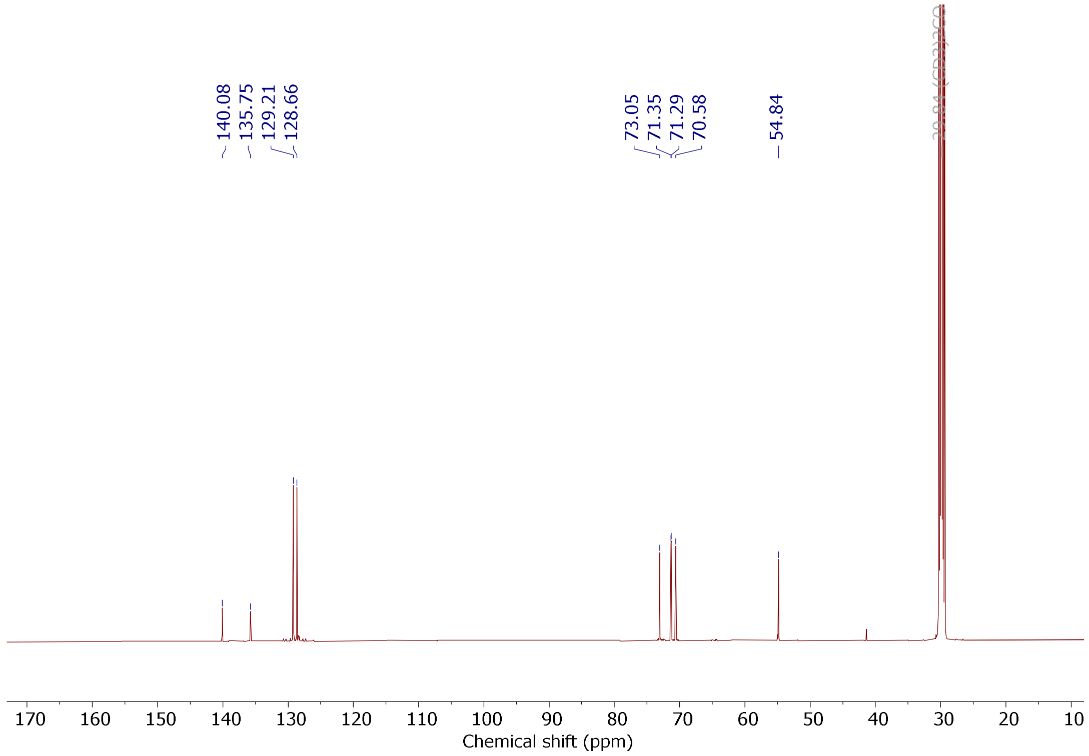

Figure S4. ^{13}C-NMR spectrum of TEG-bis(azide) ***4*** *(126 MHz, acetone-d_6, 298 K)*

General procedure 2: Synthesis of macrocycles via CuAAC reaction

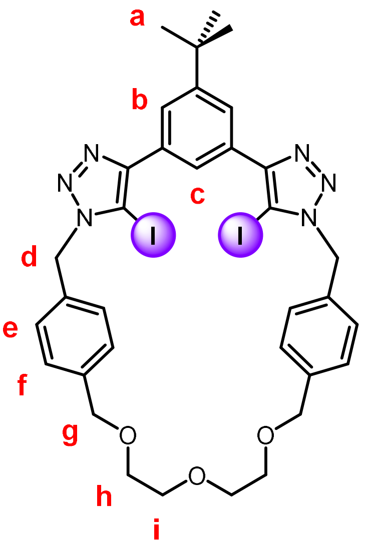

Diethylene glycol-based halogen bonding macrocycle (1·XB^{DEG}). [Cu(CH_3CN)$_4$]PF_6 (172 mg, 0.46 mmol) and TBTA (122 mg, 0.23 mmol) were dissolved in dry, degassed CH_2Cl_2 (180 mL) and the mixture was stirred at room temperature under N_2 for 15 minutes. A solution of diethylene glycol-based bis-azide **3** (365 mg, 0.92 mmol) in dry, degassed CH_2Cl_2 (2 mL) was added, followed by the dropwise addition of a solution of bis(iodoalkyne) **5** (400 mg, 0.92 mmol) in dry, degassed CH_2Cl_2 (2 mL). The reaction mixture was stirred overnight at room temperature under N_2 in the dark. The crude reaction mixture was diluted with CH_2Cl_2 and washed with aqueous EDTA/NH_4OH solution (50 mL x 2) and brine (50 mL). The organic layer was dried over anhydrous $MgSO_4$, filtered and concentrated on the rotary evaporator. The residue was purified by silica gel chromatography (eluent: 15% EtOAc/DCM) to give **1·XB^{DEG}** as a white solid (265 mg, 35%).

^{1}H NMR (500 MHz, acetone-d_6) δ (ppm) 7.94 (d, J = 1.6 Hz, 2H_b), 7.88 (d, J = 1.6 Hz, 1H_c), 7.31 (d, J = 8.1 Hz, 4H_e), 7.21 (d, J = 8.1 Hz, 4H_f), 5.78 (s, 4H_d), 4.46 (s, 4H_g), 3.61 – 3.48 (m, 8$H_{h,i}$), 1.45 (s, 9H_a).

^{13}C NMR (126 MHz, acetone-d_6) δ (ppm) 153.14, 152.29, 139.68, 135.66, 131.92, 129.20, 128.63, 125.99, 125.44, 79.01, 73.33, 71.26, 70.60, 35.72, 31.75.

HRMS (ESI +ve) m/z: 831.1000 ([M+H]$^+$, $C_{34}H_{37}O_3N_6I_2$ requires 831.1011).

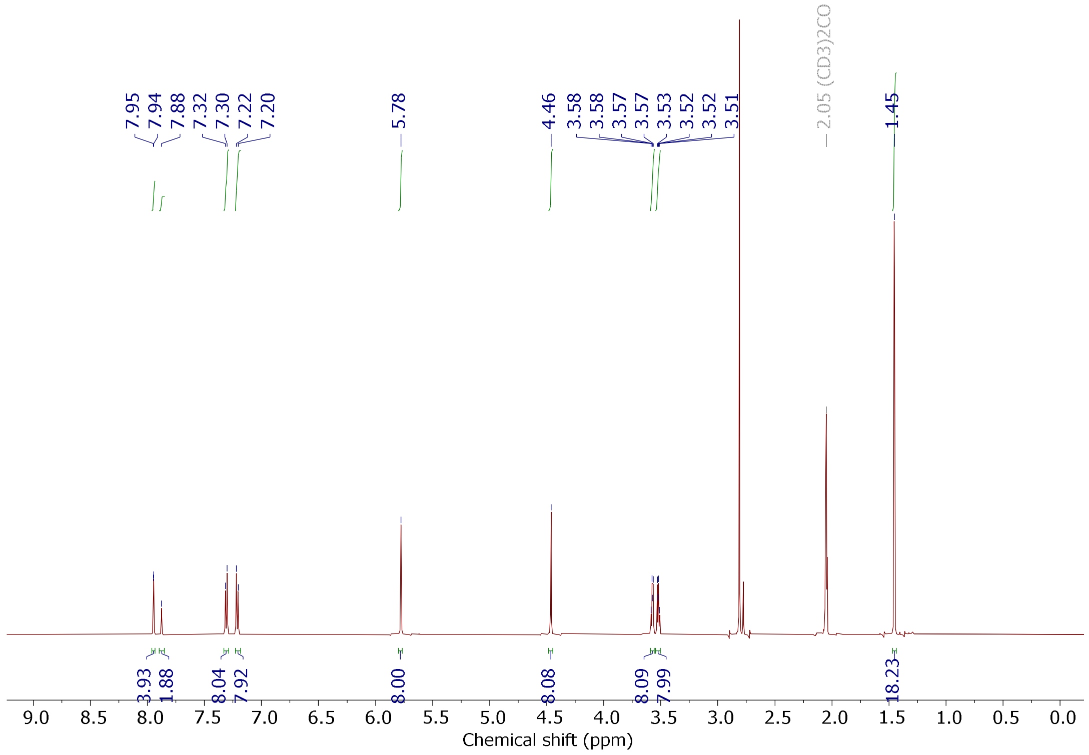

*Figure S5. ^{1}H-NMR spectrum of **1·XB^{DEG}** (500 MHz, acetone-d_6, 298 K)*

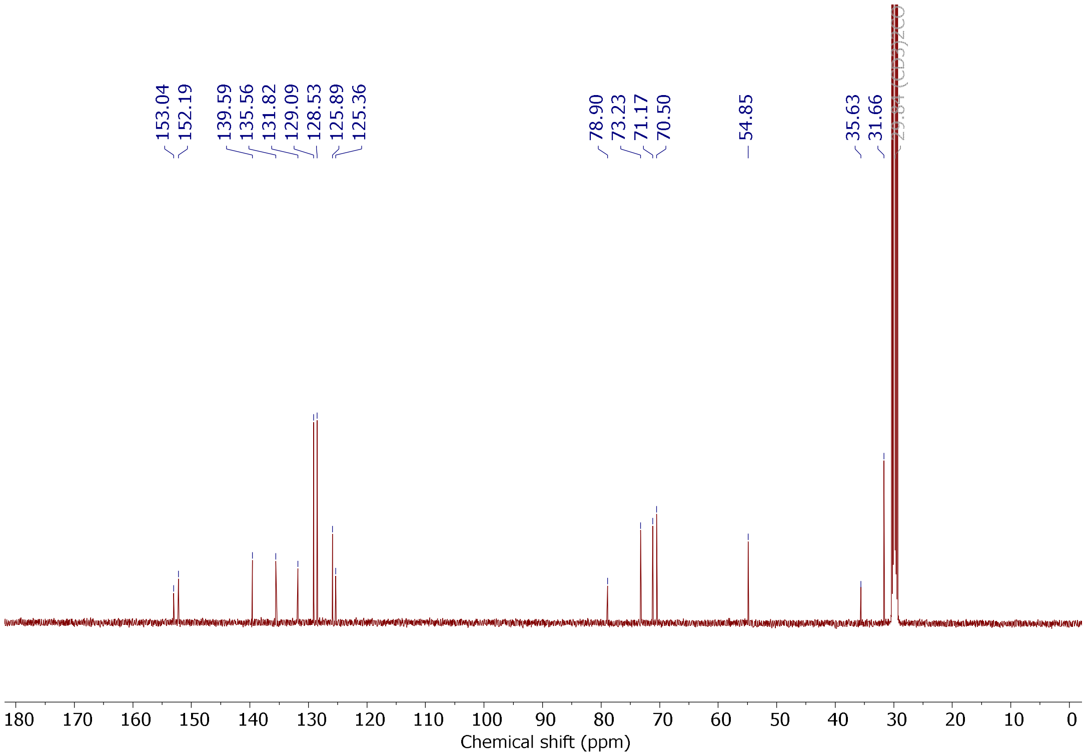

*Figure S6. ^{13}C-NMR spectrum of **1·XB^{DEG}** (126 MHz, acetone-d_6, 298 K)*

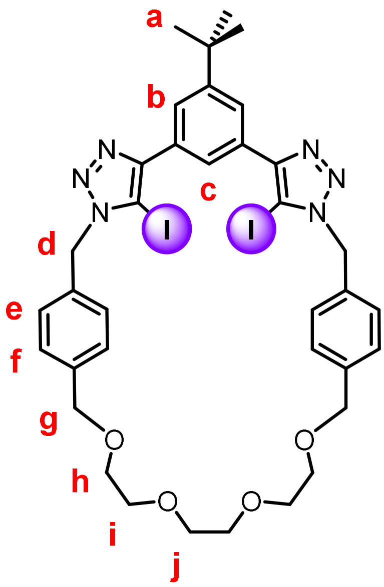

Triethylene glycol-based halogen bonding macrocycle (1·XB^{TEG}). Using general procedure 2, the CuAAC click reaction between triethylene glycol-based bis-azide **4** (391 mg, 0.89 mmol) and bis(iodoalkyne) **5** (385 mg, 0.89 mmol), followed by purification by silica gel chromatography (eluent: 30% EtOAc/DCM) yielded **1·XB^{TEG}** as a white solid (348 mg, 45%).

^{1}H NMR (500 MHz, acetone-*d*$_6$) δ (ppm) 8.12 (t, *J* = 1.6 Hz, 1H$_c$), 8.00 (d, *J* = 1.6 Hz, 2H$_b$), 7.34 (d, *J* = 8.1 Hz, 4H$_e$), 7.24 (d, *J* = 8.1 Hz, 4H$_f$), 5.79 (s, 4H$_d$), 4.50 (s, 4H$_g$), 3.61 – 3.50 (m, 12H$_{h,i,j}$), 1.45 (s, 9H$_a$).

^{13}C NMR (126 MHz, acetone-*d*$_6$) δ (ppm) 152.92, 151.69, 140.05, 135.57, 131.96, 128.84, 128.44, 125.84, 124.84, 79.32, 79.28, 73.16, 71.51, 71.40, 70.86, 35.78, 31.76.

HRMS (ESI +ve) m/z: 875.1256 ([M+H]$^+$, $C_{36}H_{41}O_4N_6I_2$ requires 875.1273).

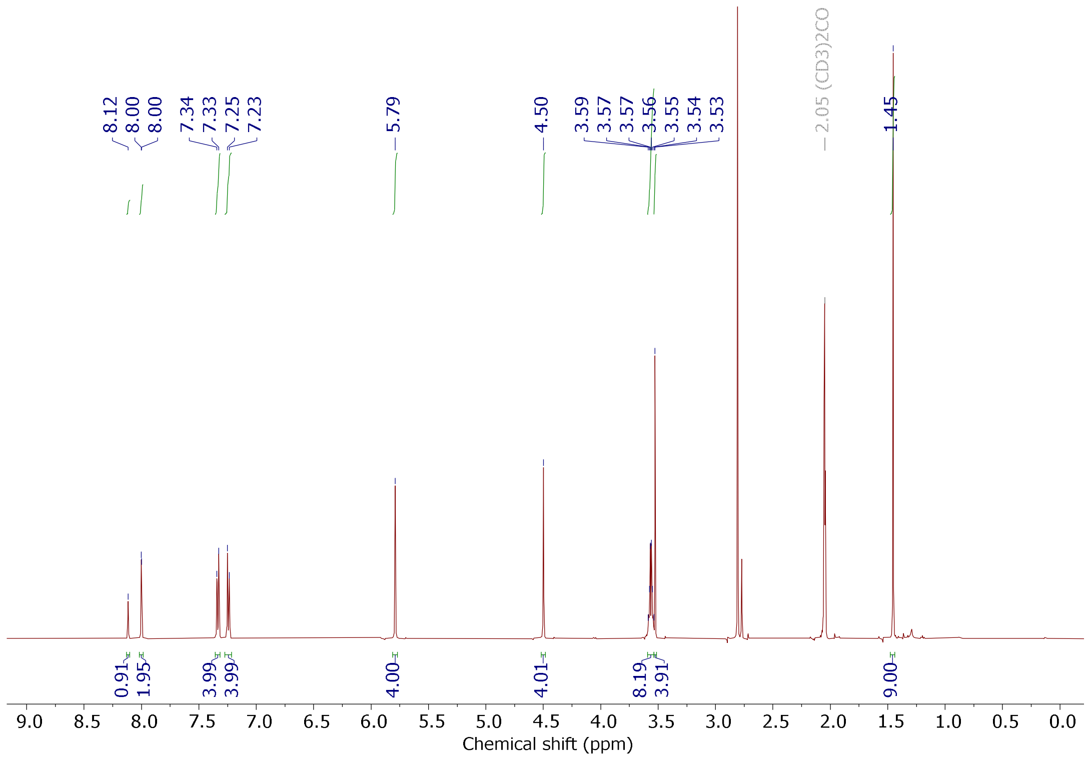

*Figure S7. ^{1}H-NMR spectrum of **1·XB^{TEG}** (500 MHz, acetone-d$_6$, 298 K)*

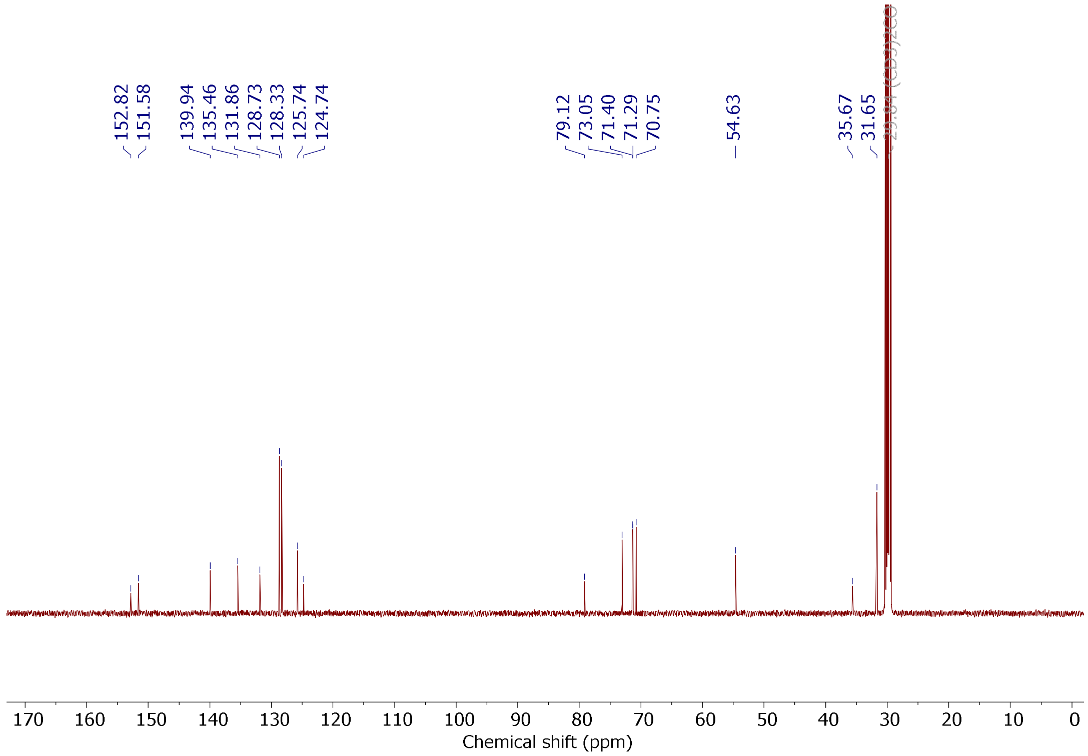

*Figure S8. ^{13}C-NMR spectrum of **1·XB^{TEG}** (126 MHz, acetone-d$_6$, 298 K)*

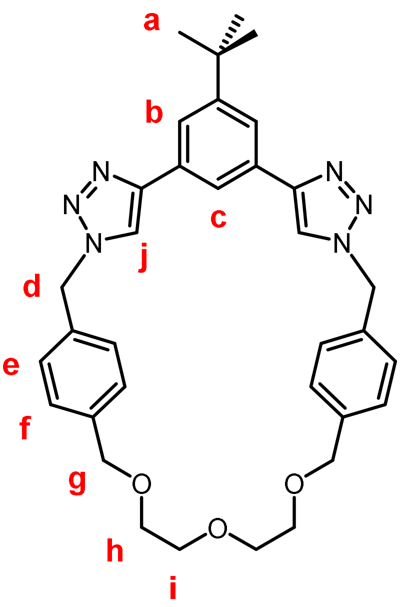

Diethylene glycol-based hydrogen bonding macrocycle ($1{\cdot}HB^{DEG}$). Using general procedure 2, the CuAAC click reaction between diethylene glycol-based bis-azide **3** (159 mg, 0.87 mmol) and bis(alkyne) **6** (346 mg, 0.87 mmol), followed by purification by silica gel chromatography (eluent: 30% EtOAc/DCM) yielded **$1{\cdot}HB^{DEG}$** as a white solid (165 mg, 33%).

^{1}H NMR (500 MHz, acetone-d_6) δ 8.17 (d, J = 1.5 Hz, 2H_b), 8.01 (s, 2H_j), 7.84 (t, J = 1.5 Hz, 1H_c), 7.38 (d, J = 8.2 Hz, 4$H_{e,f}$), 7.31 (d, J = 8.2 Hz, 4$H_{e,f}$), 5.65 (s, 4H_d), 4.52 (s, 4H_g), 3.68-3.63 (m, 8$H_{h,i}$), 1.45 (s, 9H_a).

^{13}C NMR (126 MHz, acetone-d_6) δ (ppm) 152.10, 147.82, 139.10, 134.91, 131.49, 128.41, 128.18, 121.35, 120.61, 120.18, 72.32, 70.34, 69.90, 53.30, 34.64, 30.77.

HRMS (ESI +ve) m/z: 579.3070 ($[M+H]^+$, $C_{34}H_{39}O_3N_6$ requires 579.3084).

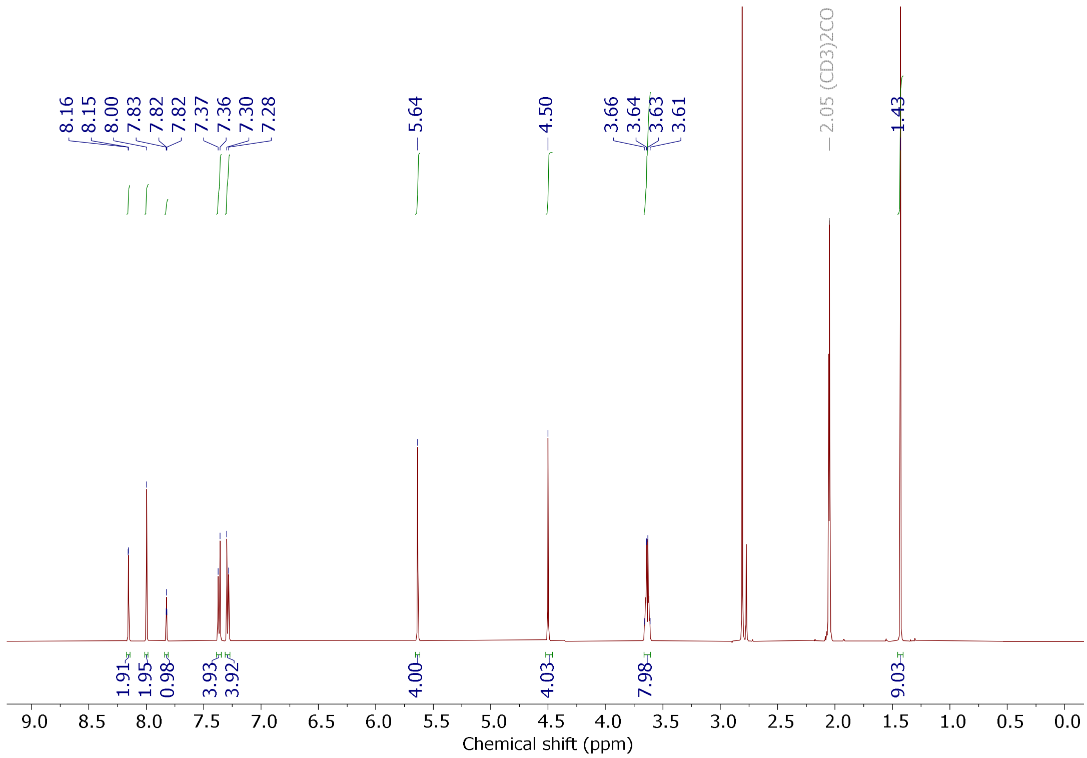

*Figure S9. ^{1}H-NMR spectrum of **1·HB^DEG** (500 MHz, acetone-d$_6$, 298 K)*

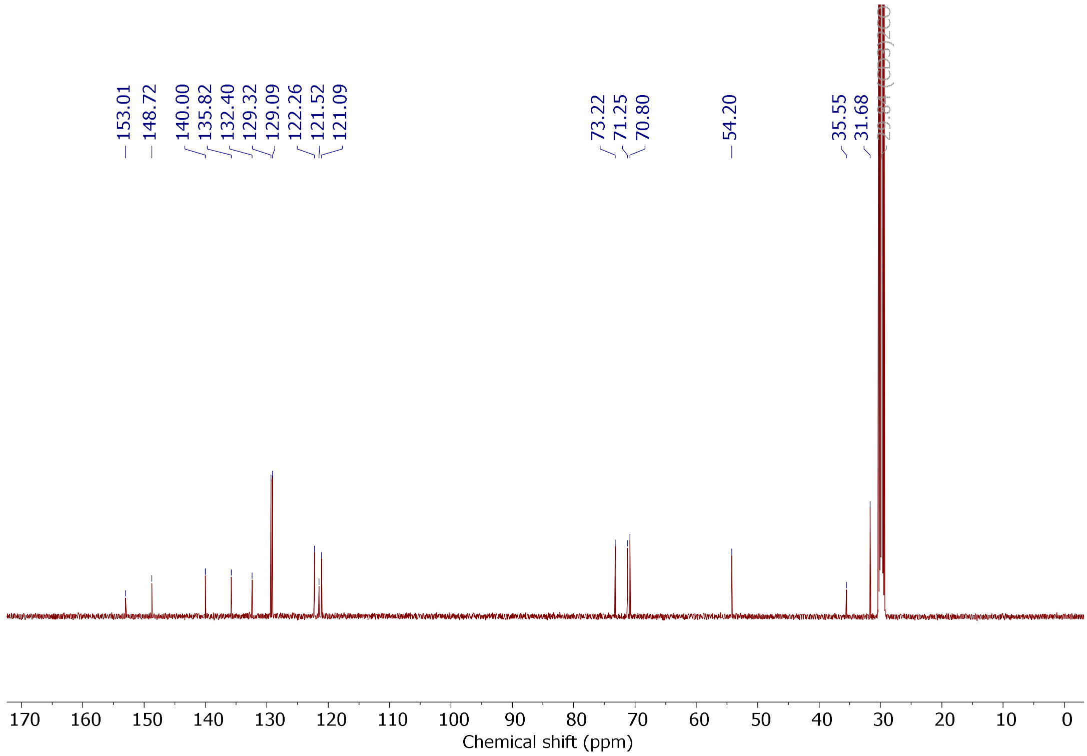

*Figure S 10. ^{13}C-NMR spectrum of **1·HB^DEG** (126 MHz, acetone-d$_6$, 298 K)*

General procedure 3: Alkali metal-templated synthesis of [2]catenanes

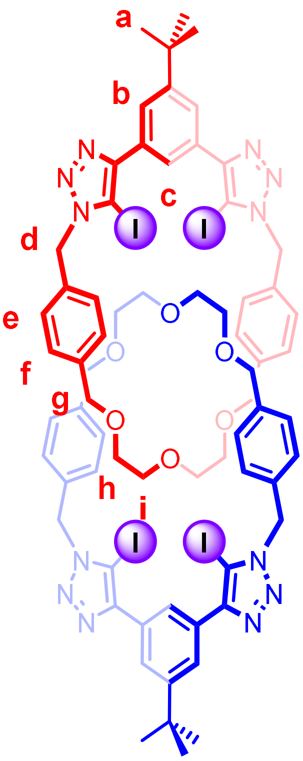

Diethylene glycol-based halogen bonding [2]catenane (2·XB^{DEG}). Macrocycle **1·XB^{DEG}** (50.0 mg, 0.060 mmol) and $NaBAr_4^F$ (53.4 mg, 0.060 mmol) were dissolved in dry, degassed CH_2Cl_2 (4 mL) and stirred for 30 minutes at room temperature. A solution of bis-azide **3** (23.9 mg, 0.060 mmol) in CH_2Cl_2 (2 mL) was added and the mixture stirred for a further 30 minutes. A solution of bis(iodoalkyne) **5** (26.1 mg, 0.060 mmol) in CH_2Cl_2 (2 mL) was added, followed by a dropwise addition of a premixed solution of $[Cu(CH_3CN)_4]PF_6$ (11.2 mg, 0.030 mmol) and TBTA (16.0 mg, 0.030 mmol) in CH_2Cl_2 (4 mL). The reaction mixture was stirred at room temperature in the dark for 48 hours, then was diluted with CH_2Cl_2 (40 mL). The organic layer was washed with EDTA/NH_4OH (2 × 25 mL) and H_2O (2 × 25 mL), dried over $MgSO_4$, filtered and concentrated under vacuum. The crude was purified by preparative TLC in 50:50:1 CH_2Cl_2/EtOAc/MeOH to afford **2·XB^{DEG}** as a white solid (20 mg, 20%).

^{1}H NMR (500 MHz, acetone-d_6) δ 7.83 (d, J = 1.6 Hz, 4H_b), 7.44 (t, J = 1.6 Hz, 2H_c), 7.17 – 7.10 (m, 8H_e), 6.89 – 6.81 (m, 8H_f), 5.62 (s, 8H_d), 3.98 (s, 8H_g), 2.99 (m, 16$H_{h,i}$), 1.44 (s, 18H_a) ppm.

^{13}C NMR (126 MHz, acetone-d_6) δ 152.66, 152.49, 139.96, 135.21, 132.08, 129.40, 129.01, 126.66, 126.01, 80.17, 73.02, 70.79, 70.02, 35.74, 32.80, 31.82 ppm.

HRMS (ESI +ve) m/z: 1661.1899 ([M+H]$^+$, $C_{68}H_{73}O_6N_{12}I_4$ requires 1661.1949).

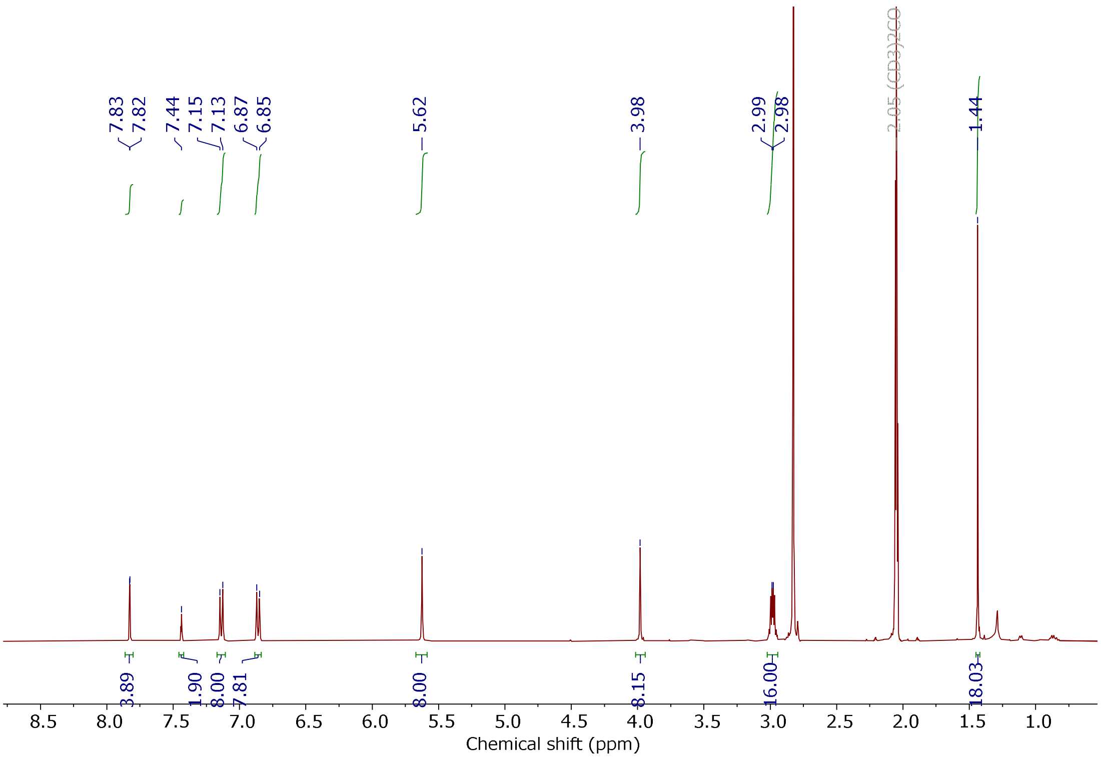

*Figure S11. ^{1}H-NMR spectrum of **2·XB^{DEG}** (600 MHz, acetone-d$_6$, 298 K)*

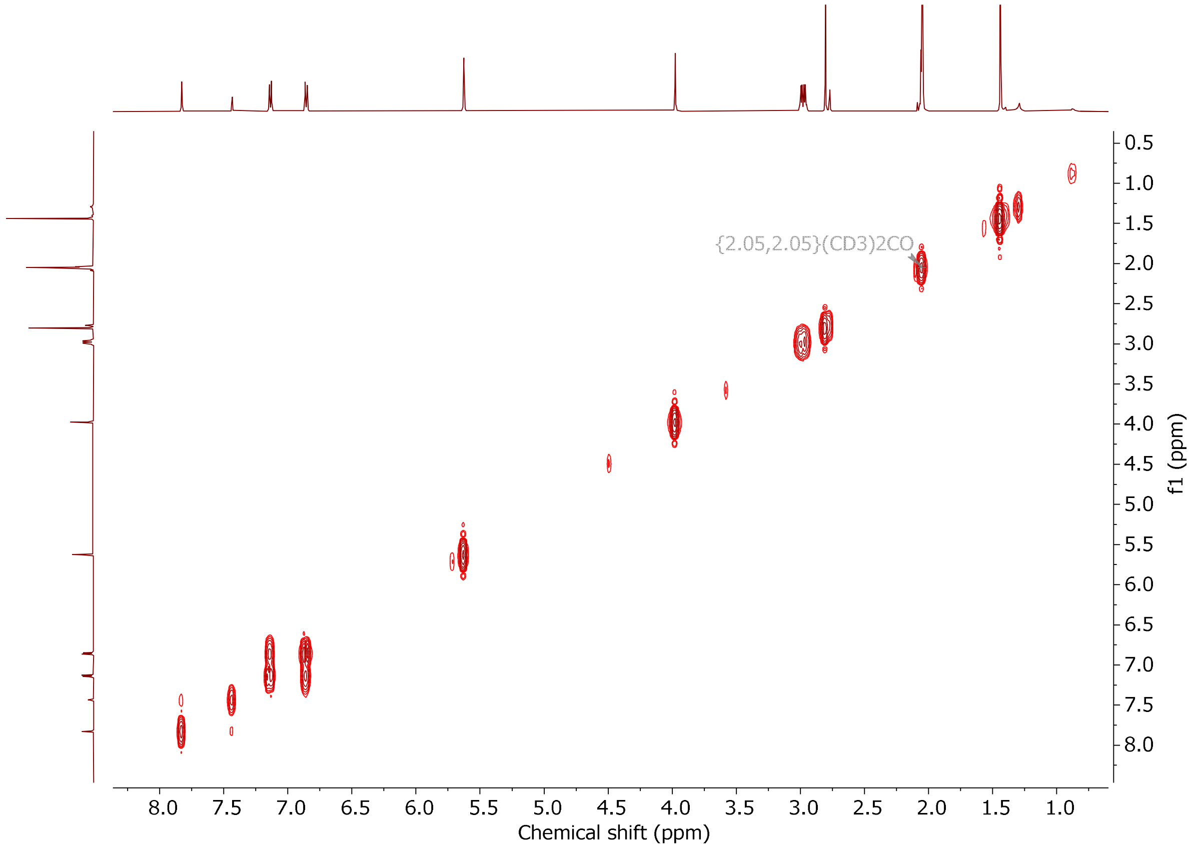

*Figure S12. ^{1}H-^{1}H COSY NMR spectrum of **2·XB^{DEG}** (500 MHz, acetone-d$_6$, 298 K)*

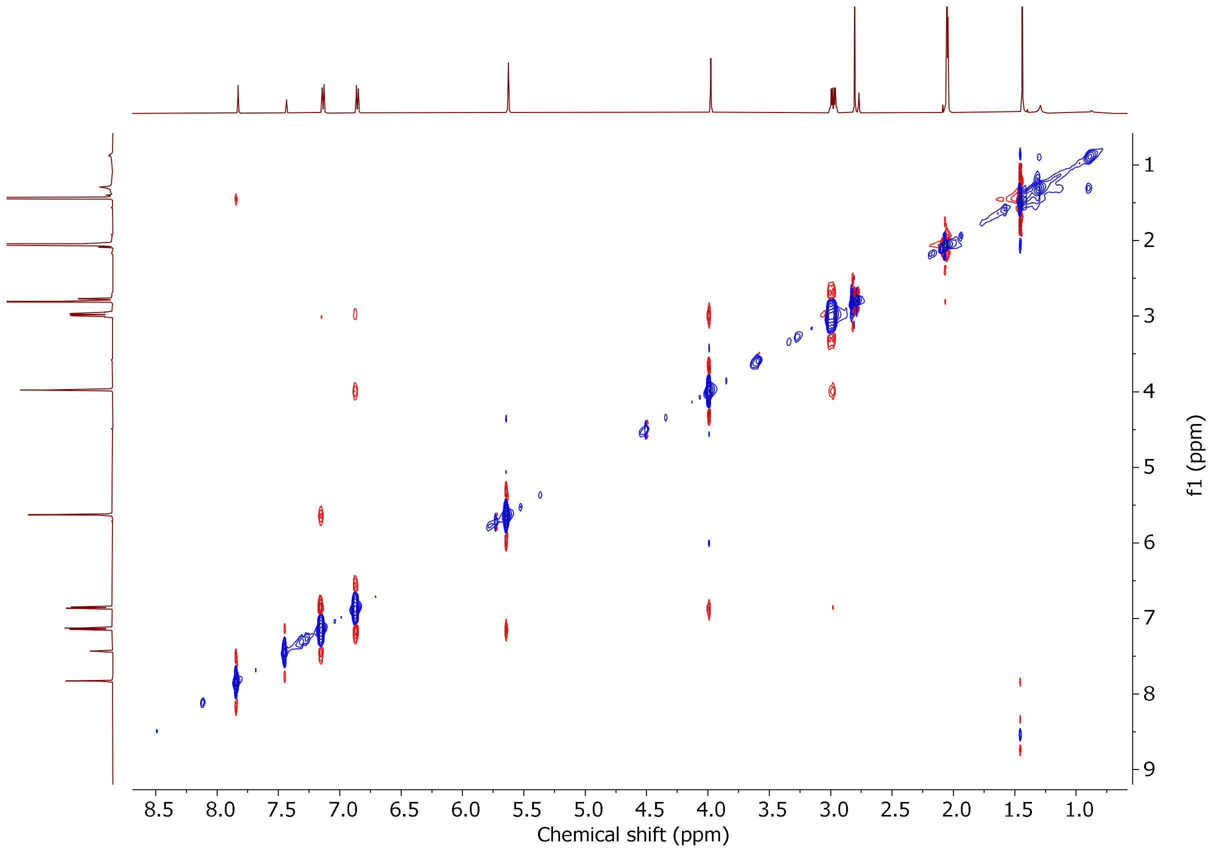

*Figure S13. 2D ^{1}H-^{1}H ROESY NMR spectrum of **2·XB^{DEG}** (500 MHz, acetone-d$_6$, 298 K)*

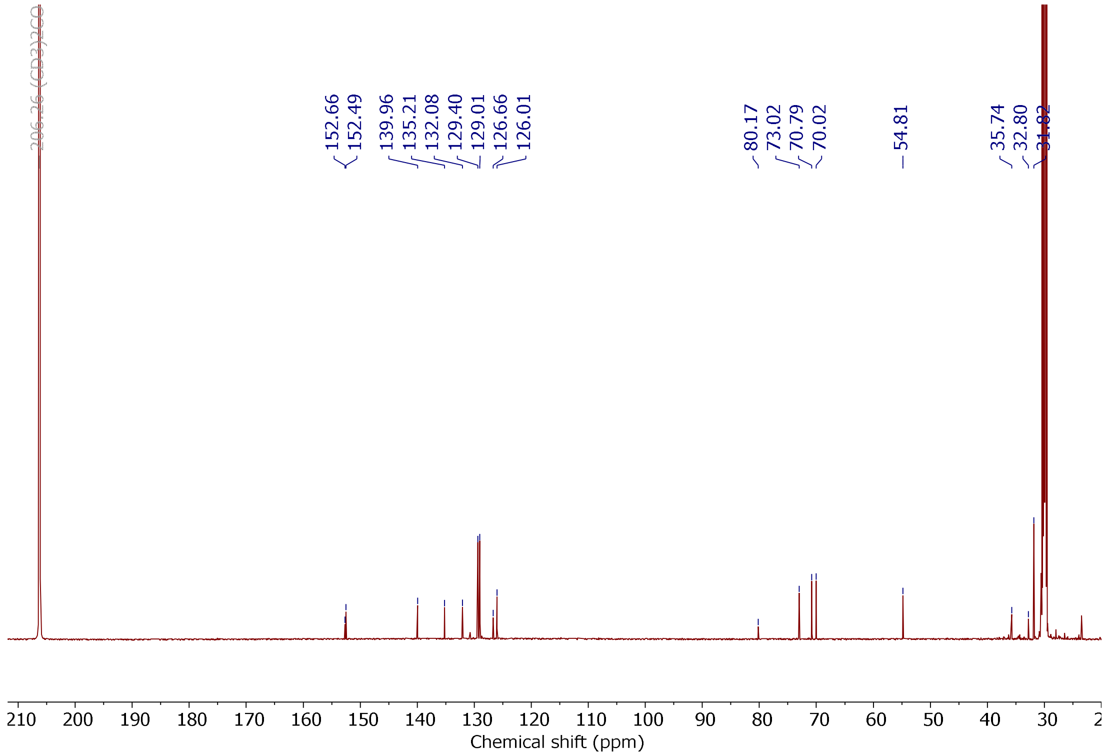

*Figure S14. ^{13}C-NMR spectrum of **2·XB^{DEG}** (151 MHz, acetone-d$_6$, 298 K)*

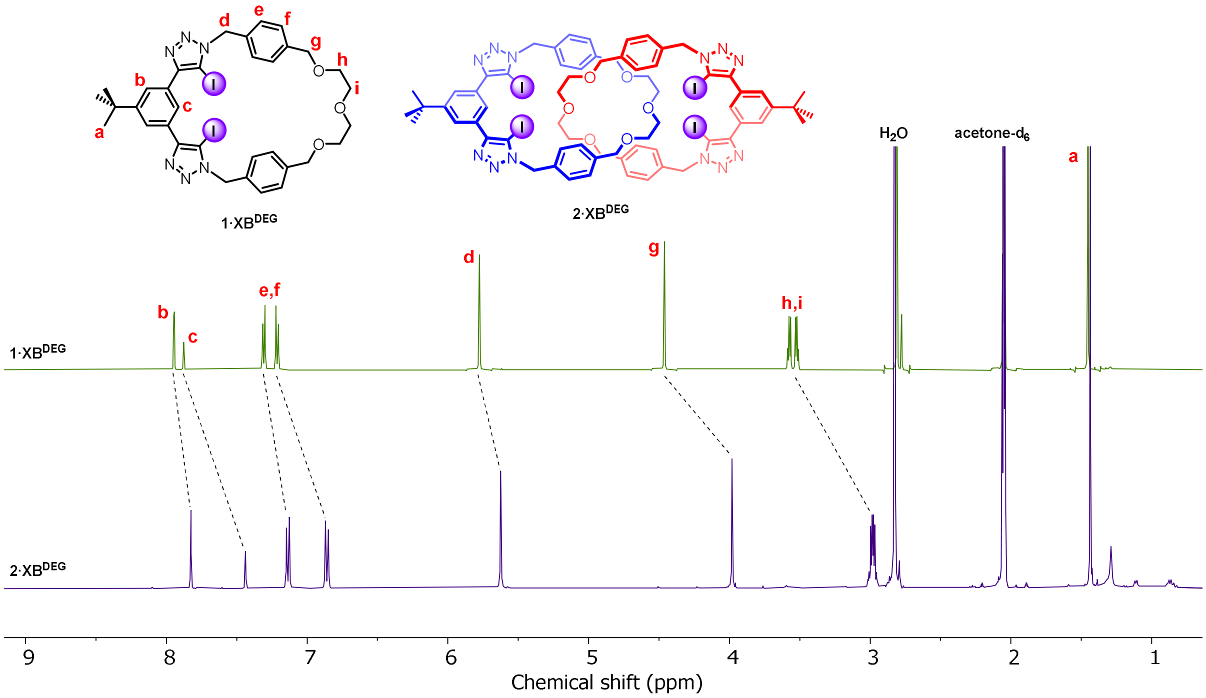

*Figure S15. Stacked ^{1}H NMR spectra of **1·XB^{DEG}** (top) and **2·XB^{DEG}** (bottom) (500 MHz, acetone-d$_6$, 298 K).*

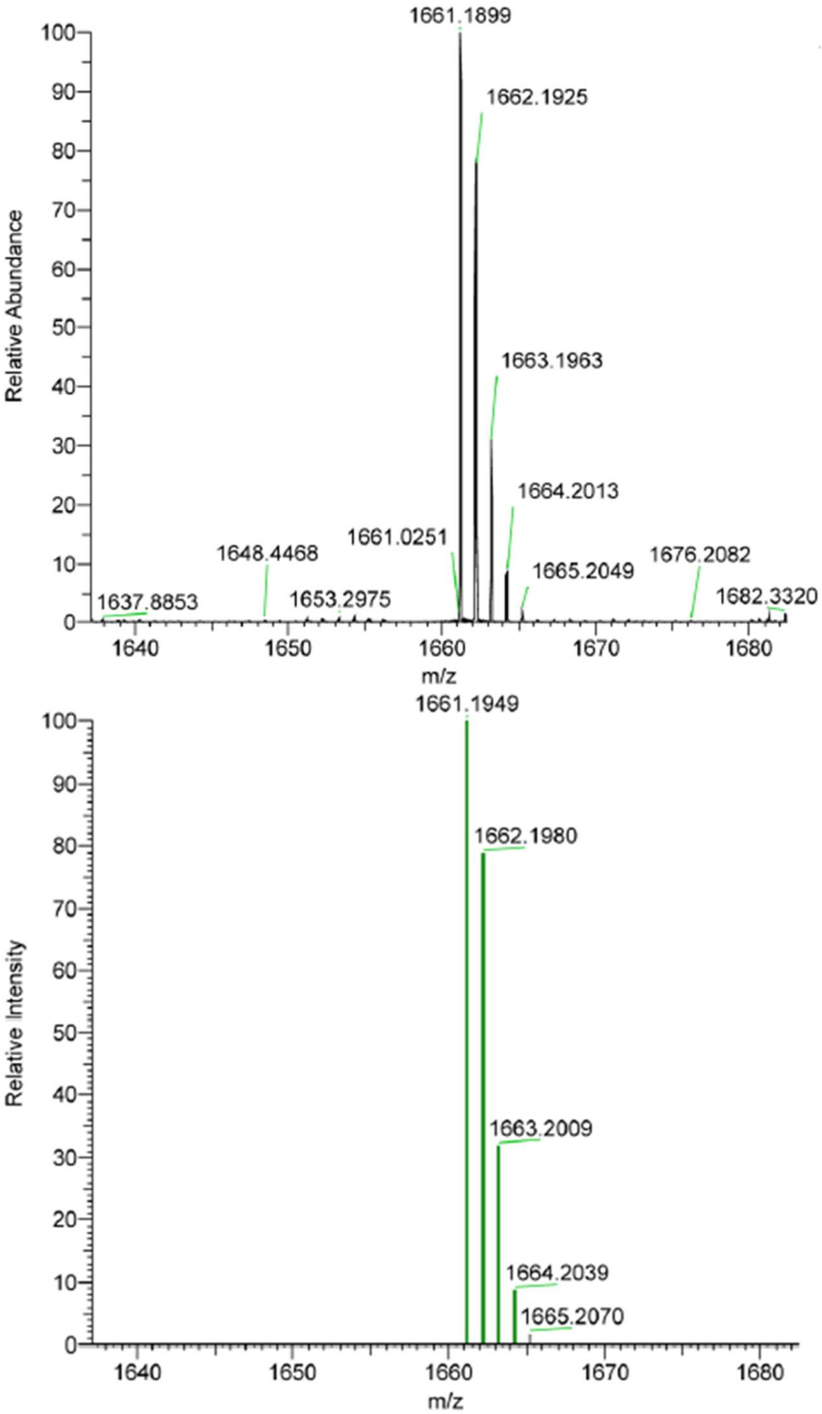

*Figure S16. High-resolution mass spectrum (ESI +ve) of **2·XB^{DEG}** (top: experimental; bottom: theoretical).*

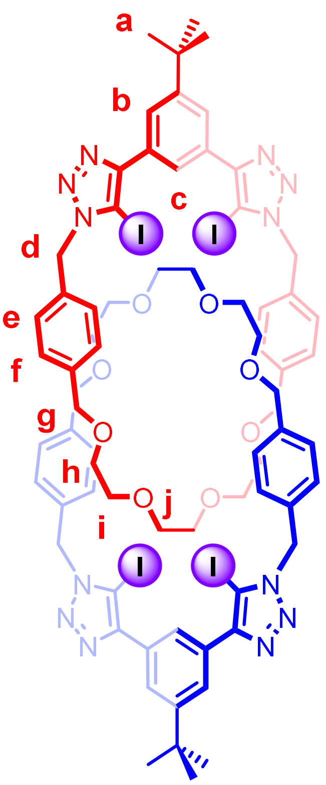

Triethylene glycol-based halogen bonding [2]catenane (2·XB^{TEG}). Following general procedure 3, the reaction between macrocycle **1·XB^{TEG}** (50.0 mg, 0.057 mmol), NaBAr$_4^F$ (50.6 mg, 0.057 mmol), bis-azide **4** (25.2 mg, 0.057 mmol), bis-iodoalkyne **5** (24.8 mg, 0.057 mmol), [Cu(CH$_3$CN)$_4$]PF$_6$ (10.7 mg, 0.026 mmol) and TBTA (15.2 mg, 0.026 mmol), followed by iterative preparative TLC purification in 50:50:2 CH$_2$Cl$_2$/EtOAc/MeOH, afforded **2·XB^{TEG}** as a white solid (18 mg, 18%).

^{1}H NMR (500 MHz, acetone-*d*$_6$) δ (ppm) 7.95 (d, *J* = 1.6 Hz, 4H$_b$), 7.84 (t, 2H$_c$), 7.09 (d, *J* = 8.0 Hz, 8H$_e$), 7.00 (d, *J* = 8.0 Hz, 8H$_f$), 5.62 (s, 8H$_d$), 4.17 (s, 8H$_g$), 3.28 – 3.17 (m, 24H$_{h,i,j}$), 1.45 (s, 18H$_a$).

^{13}C NMR (126 MHz, acetone-*d*$_6$) δ (ppm) 152.67, 151.66, 140.02, 135.21, 131.97, 128.80, 128.77, 125.75, 125.26, 79.93, 73.09, 71.34, 71.16, 70.75, 35.81, 31.84, 23.49.

HRMS (ESI +ve) m/z: 1749.2437 ([M+H]$^+$, $C_{72}H_{81}O_8N_{12}I_4$ requires 1749.2474).

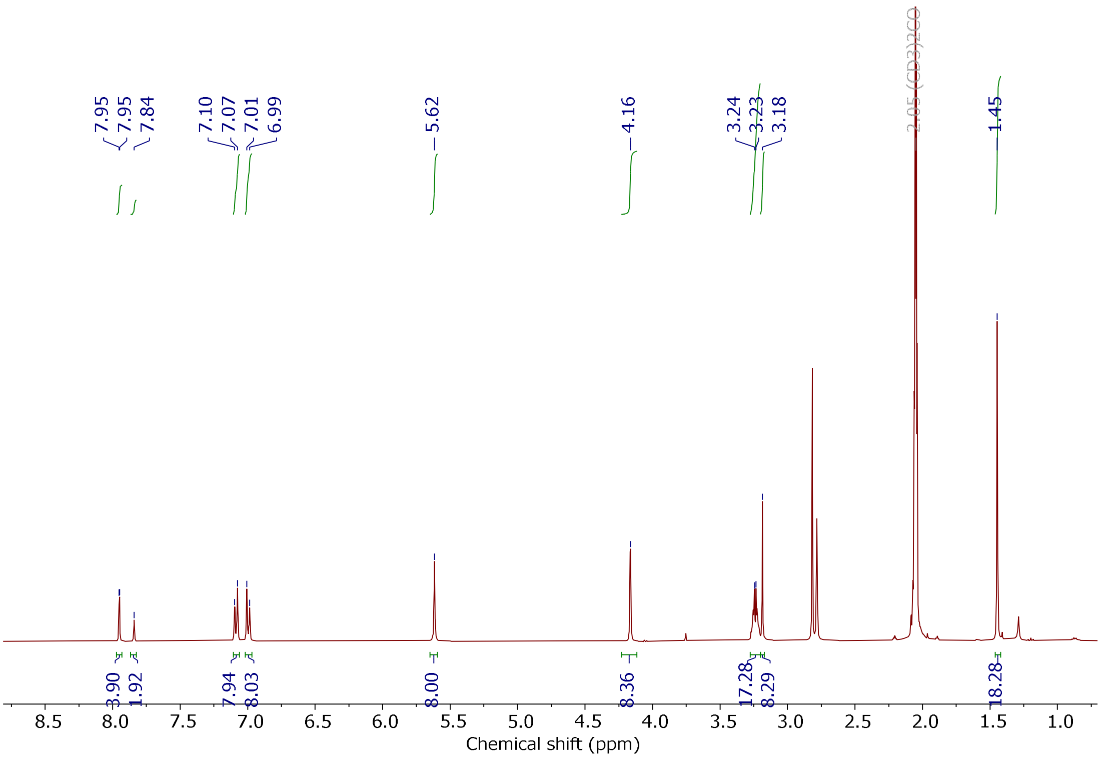

*Figure S17. ^{1}H-NMR spectrum of **2·XB^{TEG}** (600 MHz, acetone-d$_6$, 298 K)*

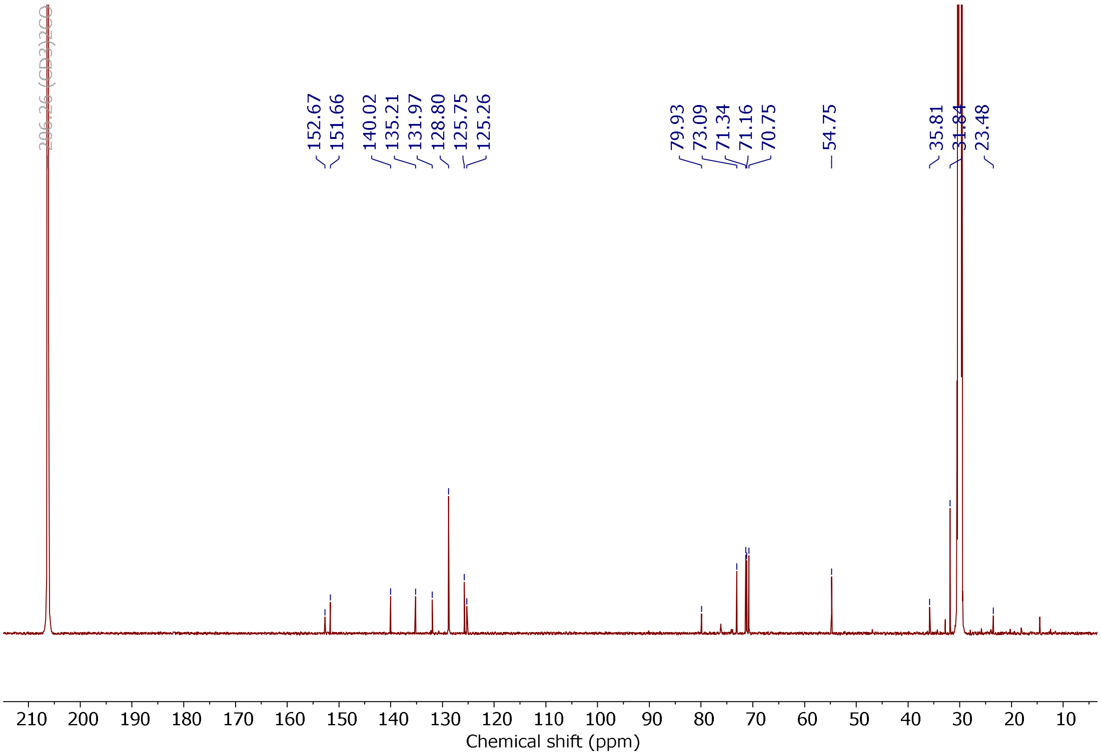

*Figure S18. ^{13}C-NMR spectrum of **2·XB^{TEG}** (151 MHz, acetone-d$_6$, 298 K)*

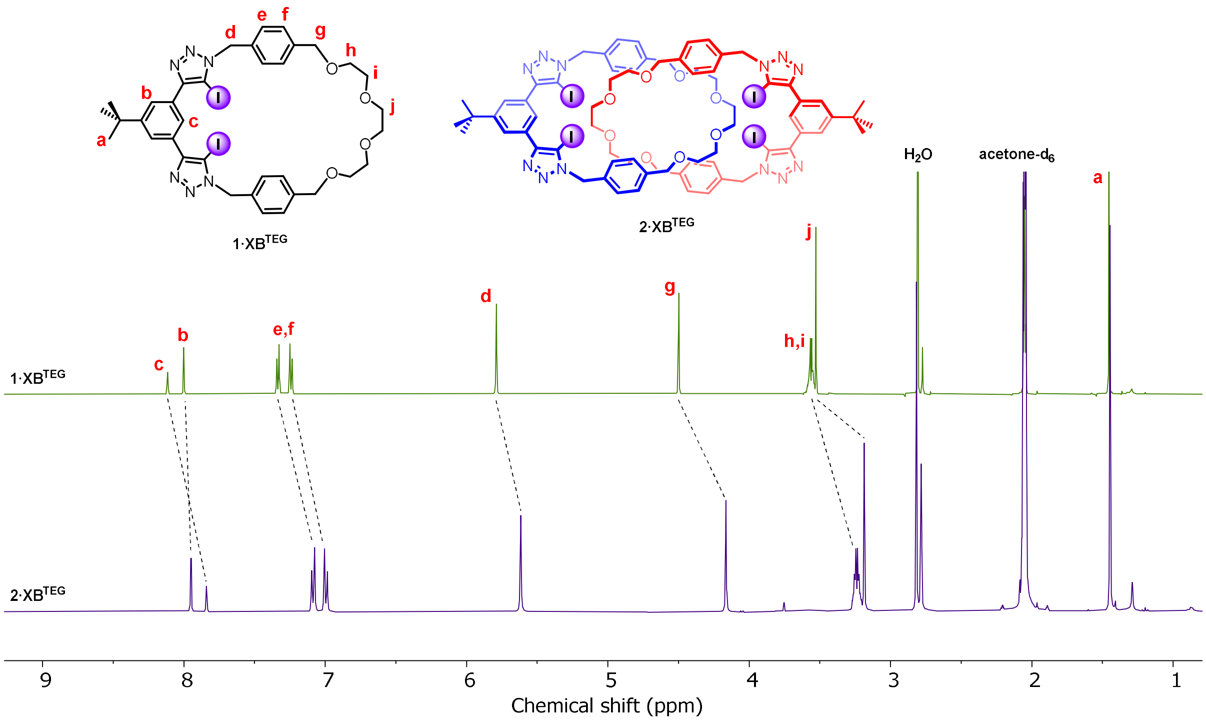

*Figure S19. Stacked ^{1}H NMR spectra of **1·XB^{TEG}** (top) and **2·XB^{TEG}** (bottom) (500 MHz, acetone-d$_6$, 298 K).*

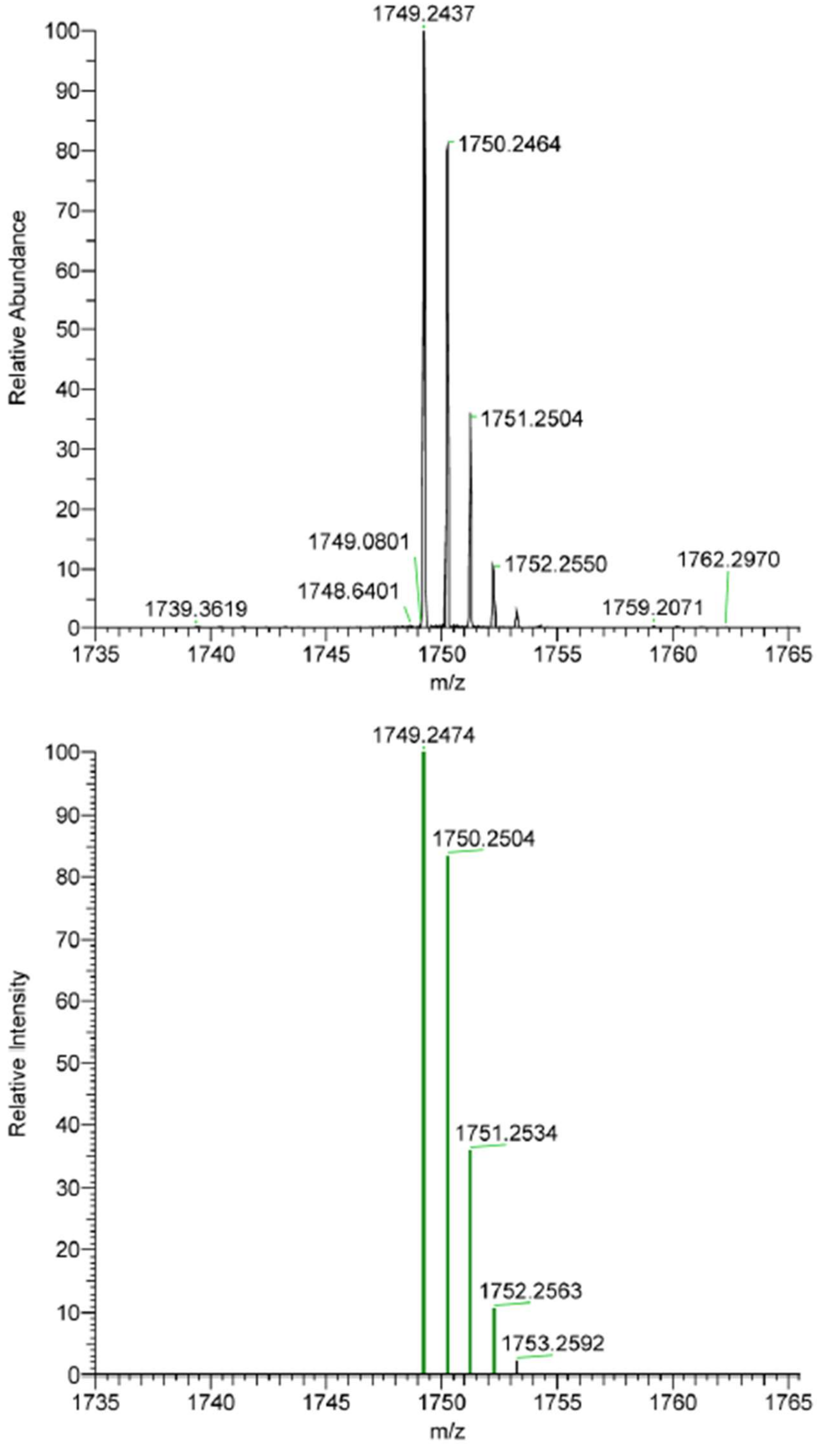

*Figure S20. High-resolution mass spectrum (ESI +ve) of **2·XB**^{TEG} (top: experimental; bottom: theoretical).*

Characterisation of ***2·XB***$^{\boldsymbol{DEG}}$ *and* ***2·XB***$^{\boldsymbol{TEG}}$ *by tandem mass spectrometry*

To ascertain that the compounds isolated from the reactions were indeed the desired homo[2]catenanes instead of the topologically isomeric [2+2]macrocycles, which are expected to have similar ^{1}H NMR spectra and identical molecular ion peaks in ESI-MS, tandem mass spectrometry experiments were conducted on the two putative [2]catenanes. The [2]catenane would be expected to contain no or few peaks with *m/z* values between that of the parent macrocycle and the molecular ion peak of the [2]catenane as any bond cleavage resulting in ring-opening would cause the two interlocked rings to dissociate. When **2·XB**$^{\mathbf{DEG}}$ was subjected to a collision energy of 40 V, the only fragments detected in the range of interest (831 < *m/z* < 1662) had *m/z* 1506.20 and 1351.42, presumably due to the loss of I+N_2 moieties from one and two iodotriazole groups respectively. Notably, the bond breaking reactions that generate these fragments do not cause ring-opening of the macrocycle and is therefore consistent with the hypothesised [2]catenane topology. Peaks with *m/z* 1594.31 and 1439.36 were observed in the MS/MS spectrum of **2·XB**$^{\mathbf{TEG}}$, likewise corresponding to sequential loss of I+N_2. For both compounds, an intense peak corresponding to a single parent macrocycle was observed, which likely arises from disassembly of the interlocked structure.

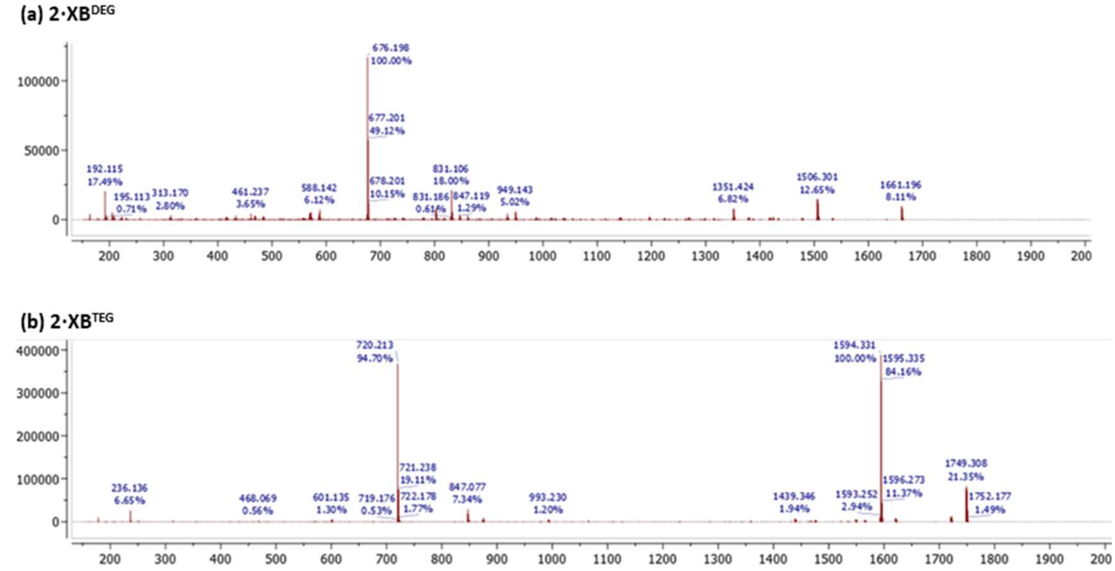

Figure S21. High resolution tandem mass spectra of a) ***2·XB***$^{\boldsymbol{DEG}}$*; b)* ***2·XB***$^{\boldsymbol{TEG}}$ *(Xevo G2-S, collision energy: 40 V).*

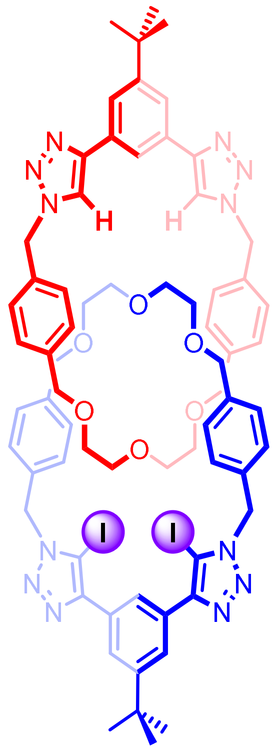

Diethylene glycol-based halogen- and hydrogen bonding [2]catenane (2·HBXB^{DEG}). Following general procedure 3, the reaction between macrocycle **1·HB^{DEG}** (50.0 mg, 0.086 mmol), NaBAr$_4^F$ (76.6 mg, 0.086 mmol), bis-azide **4** (34.3 mg, 0.086 mmol), bis-iodoalkyne **5** (37.5 mg, 0.086 mmol), $[Cu(CH_3CN)_4]PF_6$ (16.1 mg, 0.043 mmol) and TBTA (22.3 mg, 0.043 mmol), followed by iterative preparative TLC purification in 50:50:2 CH_2Cl_2/EtOAc/MeOH, afforded **2·HBXB^{DEG}** as a white solid (4.8 mg, 4%).

^{1}H NMR (500 MHz, acetone-*d*$_6$) δ (ppm) 8.17 (d, *J* = 1.5 Hz, 2H), 8.01 (s, 2H), 7.81 (s, 1H), 7.78 (d, *J* = 1.5 Hz, 2H), 7.37 (d, *J* = 8.3 Hz, 4H), 7.19 (s, 1H), 6.98 (d, *J* = 8.3 Hz, 4H), 6.79 (d, *J* = 8.1 Hz, 4H), 6.62 (d, *J* = 8.1 Hz, 4H), 5.71 (s, 4H), 5.13 (s, 4H), 4.20 (s, 4H), 3.61 – 3.57 (m, 4H), 3.49 – 3.44 (m, 4H), 2.89 – 2.85 (m, 4H), 2.83 – 2.81 (m, 4H), 2.77 (s, 4H), 1.45 (s, 9H), 1.42 (s, 9H).

^{13}C NMR (126 MHz, acetone-*d*$_6$) δ (ppm) 152.76, 152.44, 152.28, 148.44, 139.52, 139.40, 135.86, 134.81, 132.62, 131.75, 130.00, 129.41, 128.81, 126.86, 125.69, 121.94, 121.89, 73.31, 72.92, 71.19, 70.98, 70.55, 69.75, 54.55, 54.16, 35.57, 35.55, 31.79, 31.66, 24.42, 23.33, 14.36, 13.84.

HRMS (ESI +ve) m/z: 1409.4000 ($[M+H]^+$, $C_{68}H_{77}O_6N_{12}$ requires 1409.4016).

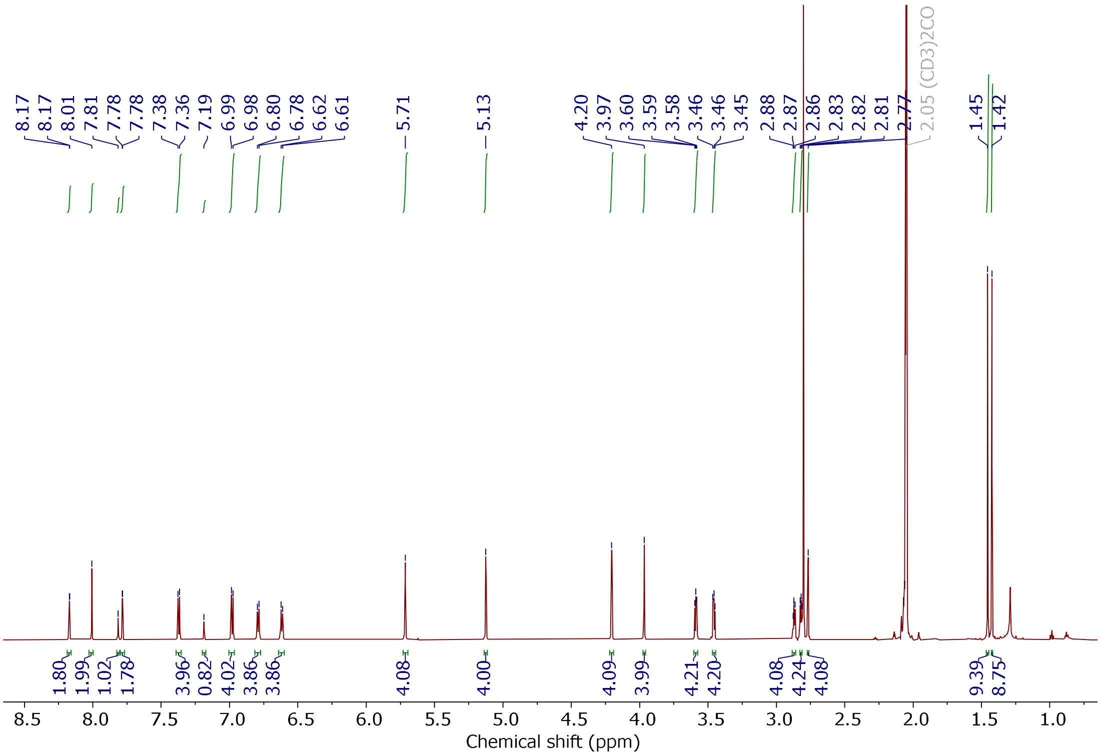

*Figure S22. ^{1}H-NMR spectrum of **2·HBXB^{DEG}** (600 MHz, acetone-d$_6$, 298 K)*

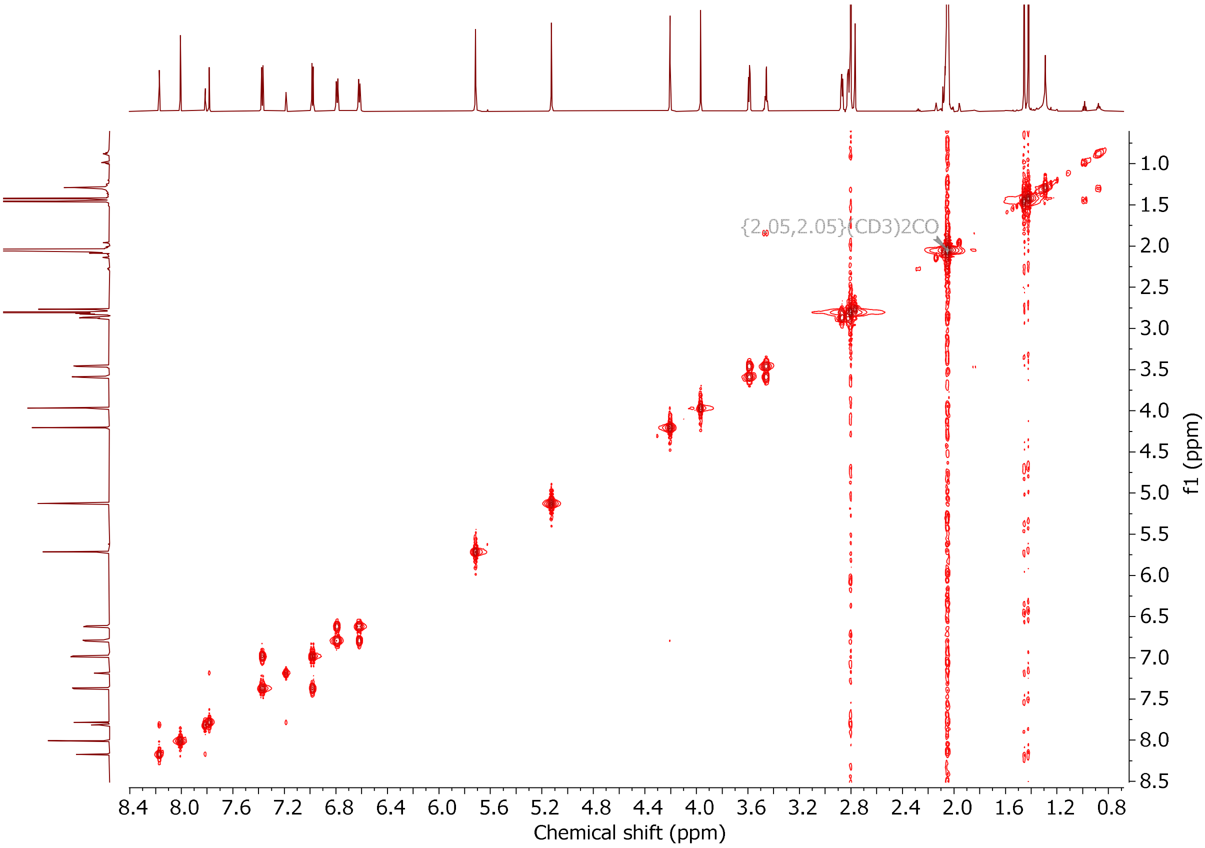

*Figure S23. ^{1}H-^{1}H COSY NMR spectrum of **2·HBXB^{DEG}** (700 MHz, acetone-d$_6$, 298 K).*

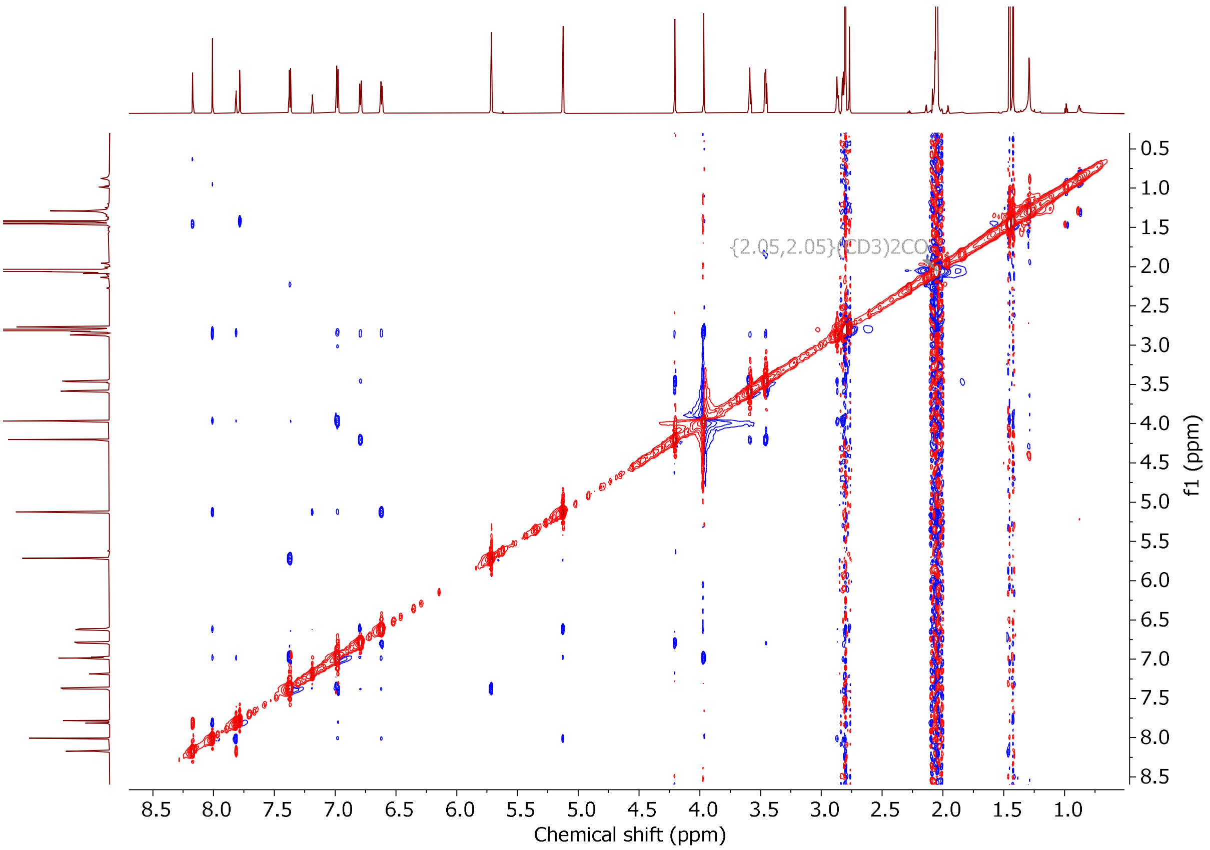

*Figure S24. 2D ^{1}H-^{1}H ROESY NMR spectrum of **2·HBXB^{DEG}** (700 MHz, acetone-d_6, 298 K).*

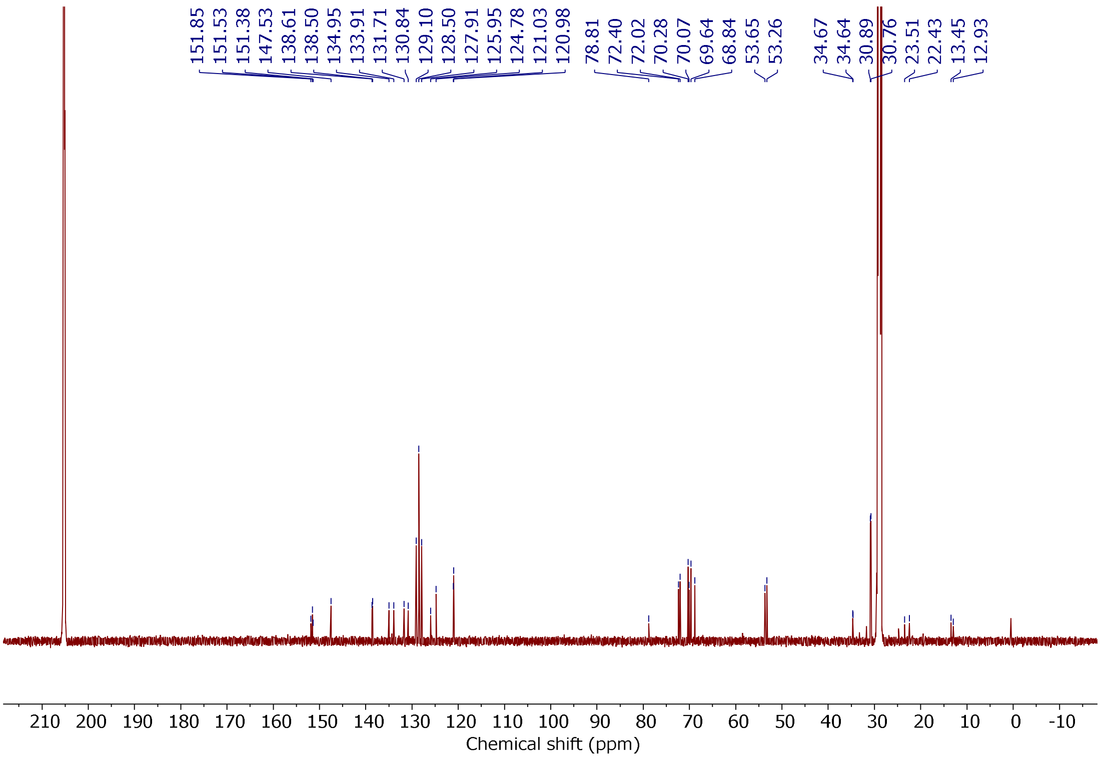

*Figure S25. ^{13}C-NMR spectrum of **2·HBXB^{DEG}** (151 MHz, acetone-d_6, 298 K)*

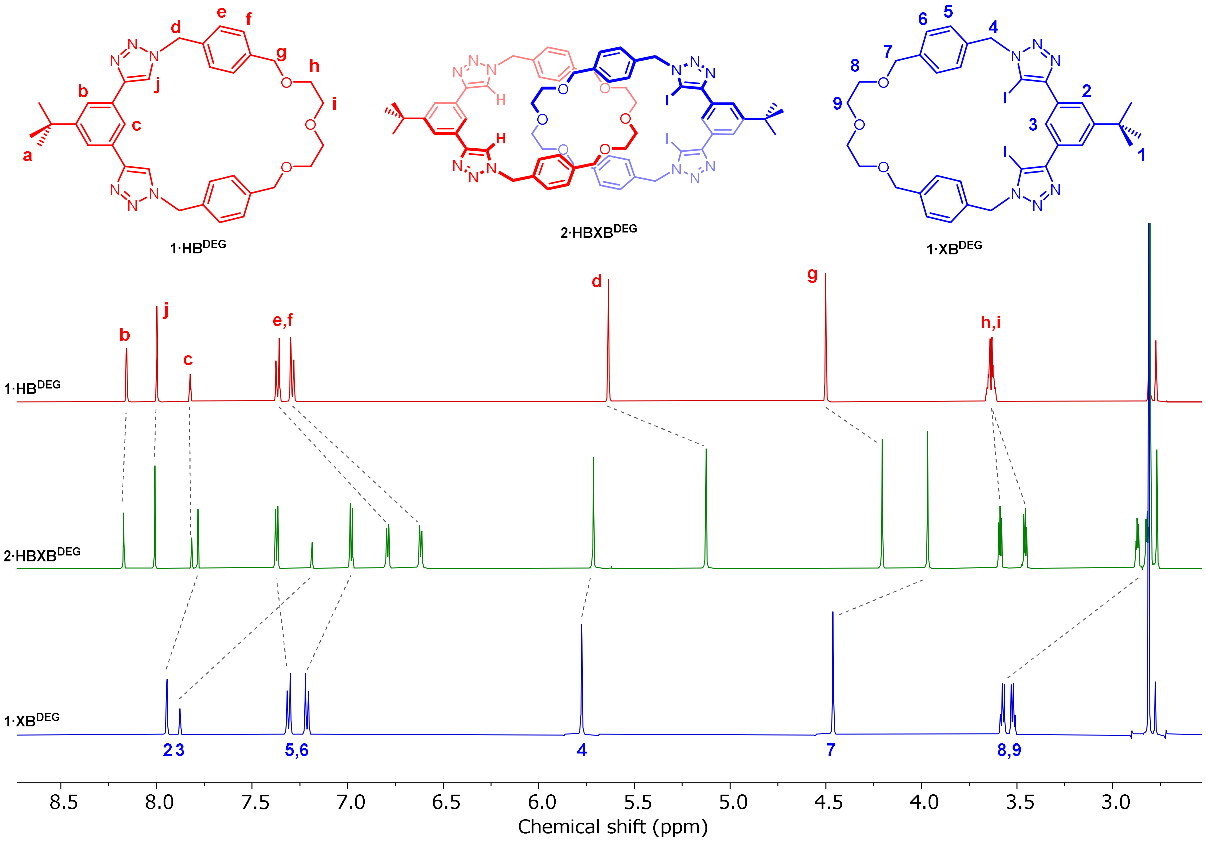

*Figure S26. Stacked ^{1}H NMR spectra of **1·HB^{DEG}** (top), **2·HBXB^{DEG}** (middle) and **1·XB^{DEG}** (bottom) (500 MHz. acetone-d_6, 298 K).*

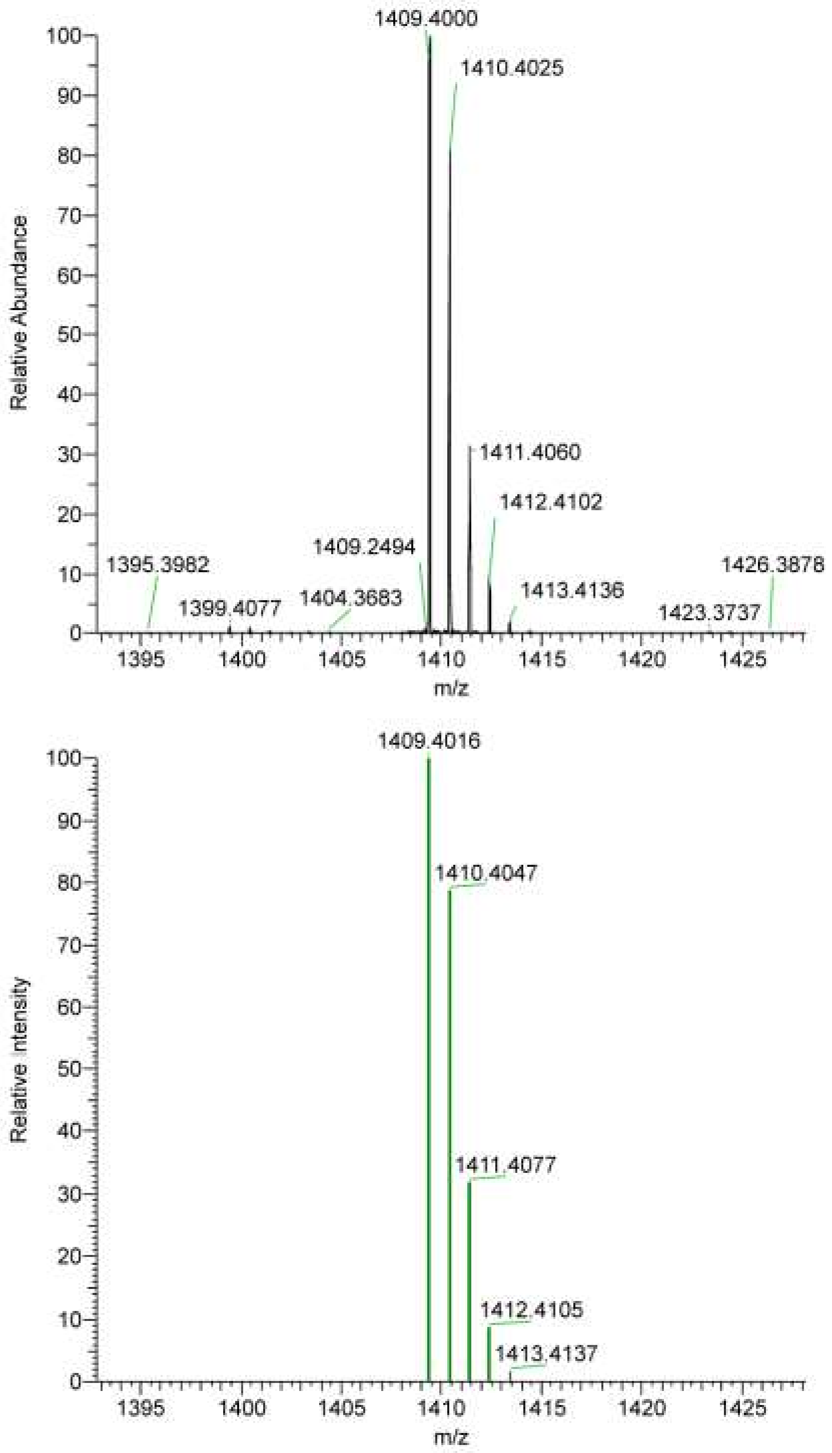

*Figure S27. High-resolution mass spectrum (ESI +ve) of **2·HBXB^{DEG}** (top: experimental; bottom: theoretical).*

^{1}H NMR pseudorotaxane studies

General Procedure

Qualitative pseudo-[2]rotaxane studies were undertaken to demonstrate the self-assembly of the macrocycles and with the respective bis(azide) precursor in the presence of a metal cation template. In a typical experiment, four separate $CDCl_3$ solutions were prepared, containing: the macrocycle, the bis(azide), the macrocycle with an equimolar amount of $MBAr^F$ (M = Na, K), and the macrocycle with equimolar amounts of the bis(azide) and $MBAr^F$. All species were present at a concentration of 4.0 mM. The ^{1}H NMR spectra of the solutions were recorded on a Bruker AVIII 500 MHz spectrometer at 298 K.

^{1}H NMR spectra

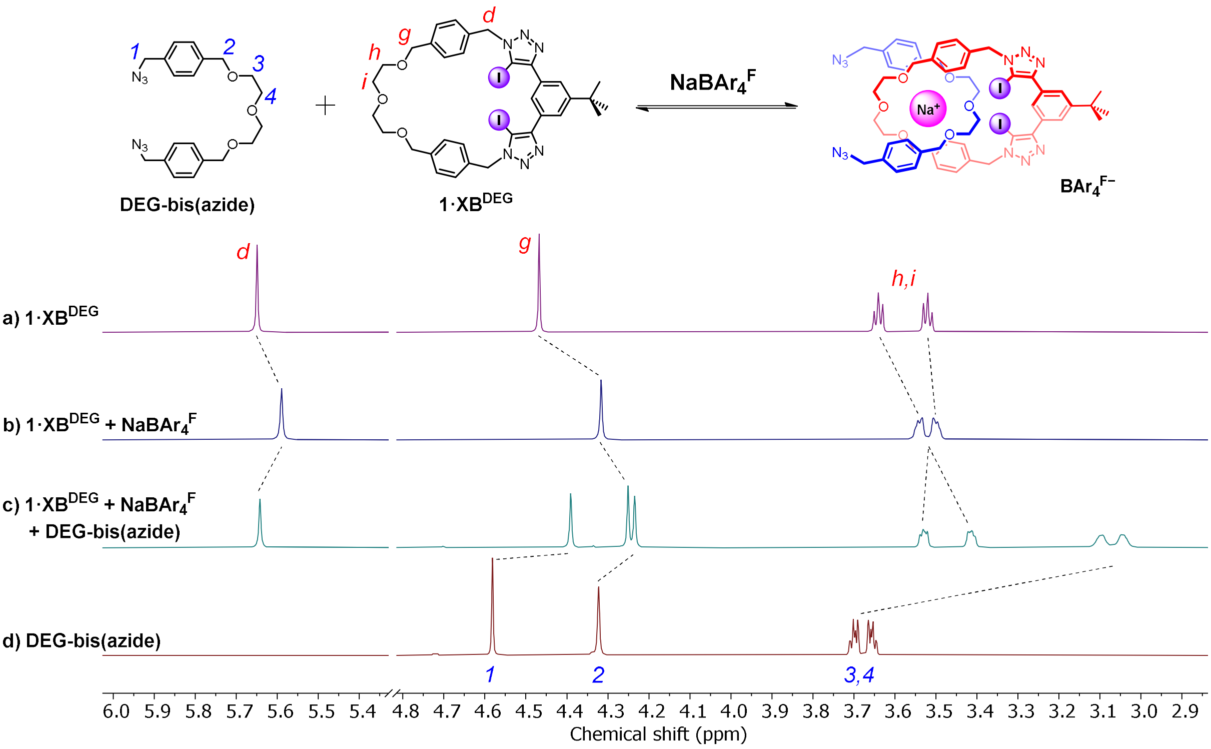

*Figure S28. Stacked ^{1}H NMR spectra of a) **1·XB^{DEG}**; b) **1·XB^{DEG}** + 1 eq. $NaBAr^F$; c) **1·XB^{DEG}** + 1 eq. $NaBAr^F$ + 1 eq. DEG-bis(azide); d) DEG-bis(azide)*

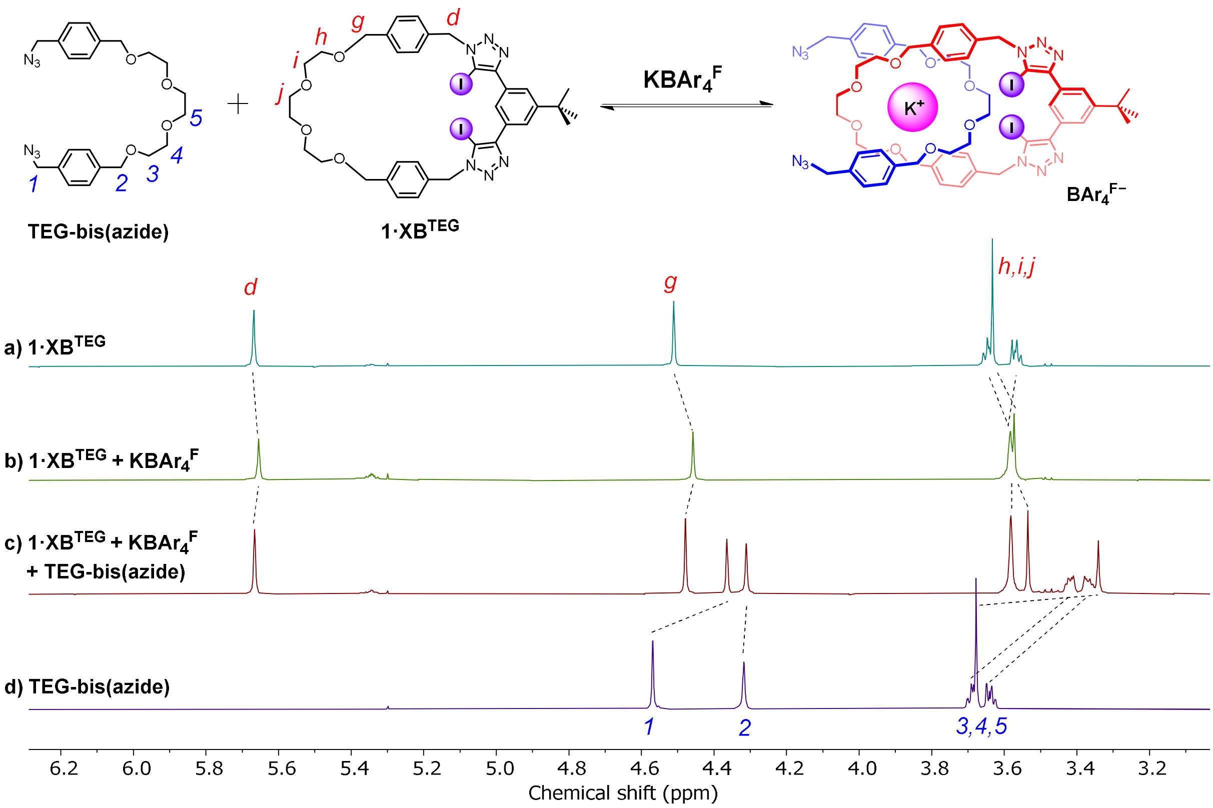

Figure S29. Stacked ^{1}H NMR spectra of a) $1{\cdot}XB^{TEG}$; b) $1{\cdot}XB^{TEG}$ + 1 eq. $KBAr^F$; c) $1{\cdot}XB^{TEG}$ + 1 eq. $KBAr^F$ + 1 eq. TEG-bis(azide); d) TEG-bis(azide)

^{1}H NMR binding studies

General procedure

All ^{1}H NMR titration experiments were performed on a Bruker AVIII 500 MHz spectrometer at 298 K. In a typical cation or anion titration, a 1.0 mM solution of the neutral receptor was prepared in 1:1 $CDCl_3/CD_3CN$. In an ion-pair titration, an equimolar amount of the [2]catenane and M^IBAr^F, each present at 1.0 mM concentration, was dissolved in 1:1 $CDCl_3/CD_3CN$. The solution was sonicated for 20 min to form the metal-catenane complex. A 50 mM solution of M^IBAr^F (M = Na, K) or TBAX (X = Cl, Br, I) was added in aliquots to the solution containing the receptor, where 1.0 equivalent of the salt added corresponds to 10.0 µL of the salt solution. 17 spectra were recorded, corresponding to 0.0, 0.2, 0.4, 0.6, 0.8, 1.0, 1.2, 1.4, 1.6, 1.8, 2.0, 2.5, 3.0, 4.0, 5.0, 7.0, 10.0 equivalents of the added guest ion. The binding of cations and anions to all receptors were found to be fast on the NMR timescale. For cation titrations, the chemical shifts of multiple peaks around the cation binding site (H_d, H_e, H_f, H_g, $H_{h/i}$) were monitored and used for subsequent fitting. For anion and ion-pair titrations, the chemical shift of the internal benzene proton H_c was used. The values of the observed chemical shift(s) and concentration of guest at each titration point were entered into the Bindfit software alongside initial estimates of the binding constants and limiting chemical shifts. These parameters were refined using nonlinear least-squares analyses to obtain the best fit between empirical and calculated chemical shifts based on five host-guest binding models (1:1, 1:2 Full, 1:2 Non-cooperative, 1:2 Additive and 1:2 Non-statistical). The input parameters were varied until convergence of the best fit values of the binding constants was attained.

^{1}H NMR titration spectra

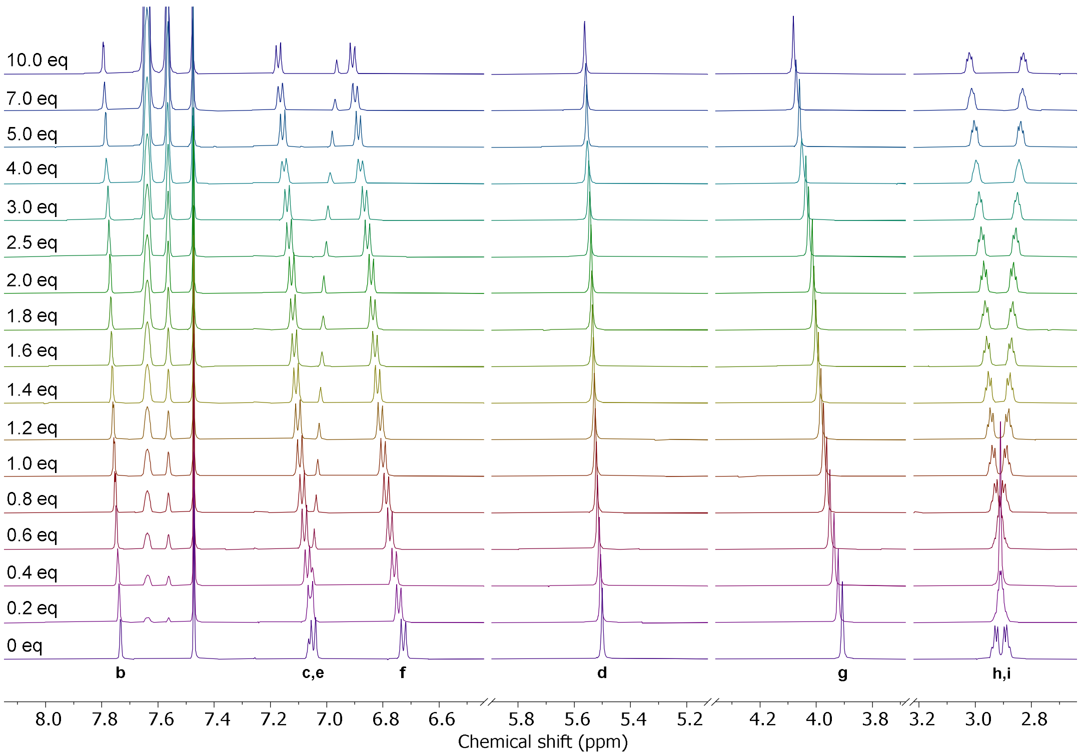

*Figure S30. Truncated ^{1}H NMR titration spectra of [2]catenane **2·XB^{DEG}** upon progressive addition of 10 equivalents NaBAr^F (500 MHz, 298 K, 1:1 $CDCl_3/CD_3CN$, [Receptor] = 1.0 mM).*

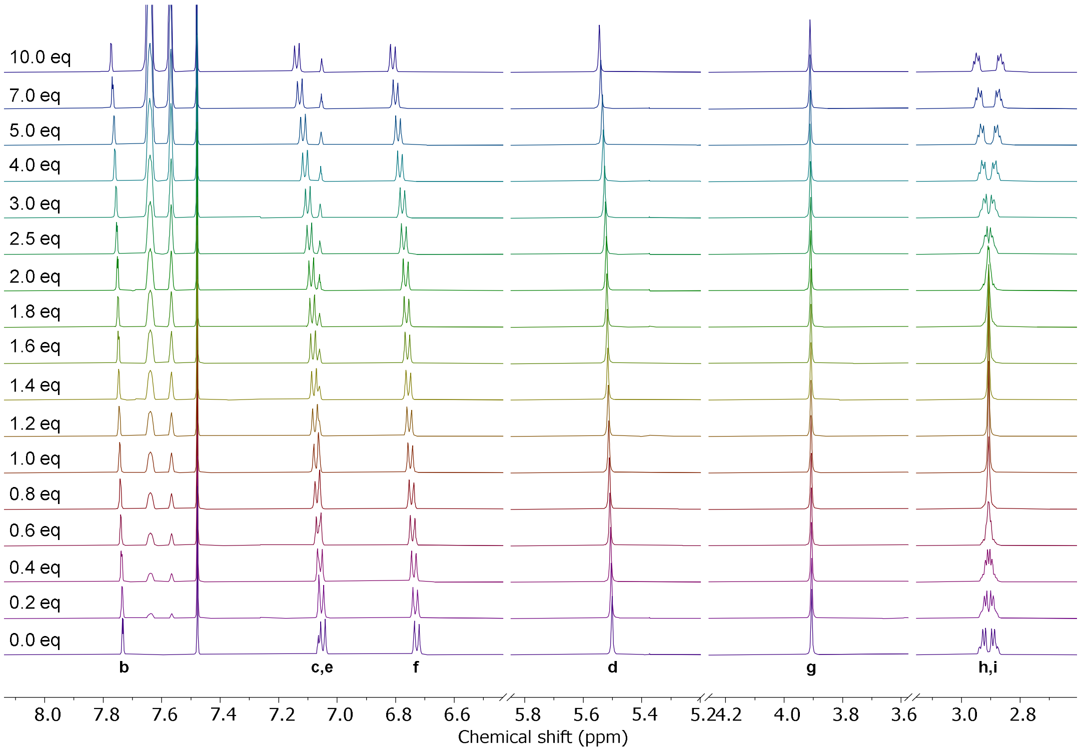

*Figure S31. Truncated ^{1}H NMR titration spectra of [2]catenane **2·XB^{DEG}** upon progressive addition of 10 equivalents KBAr^{F} (500 MHz, 298 K, 1:1 $CDCl_3/CD_3CN$, [Receptor] = 1.0 mM).*

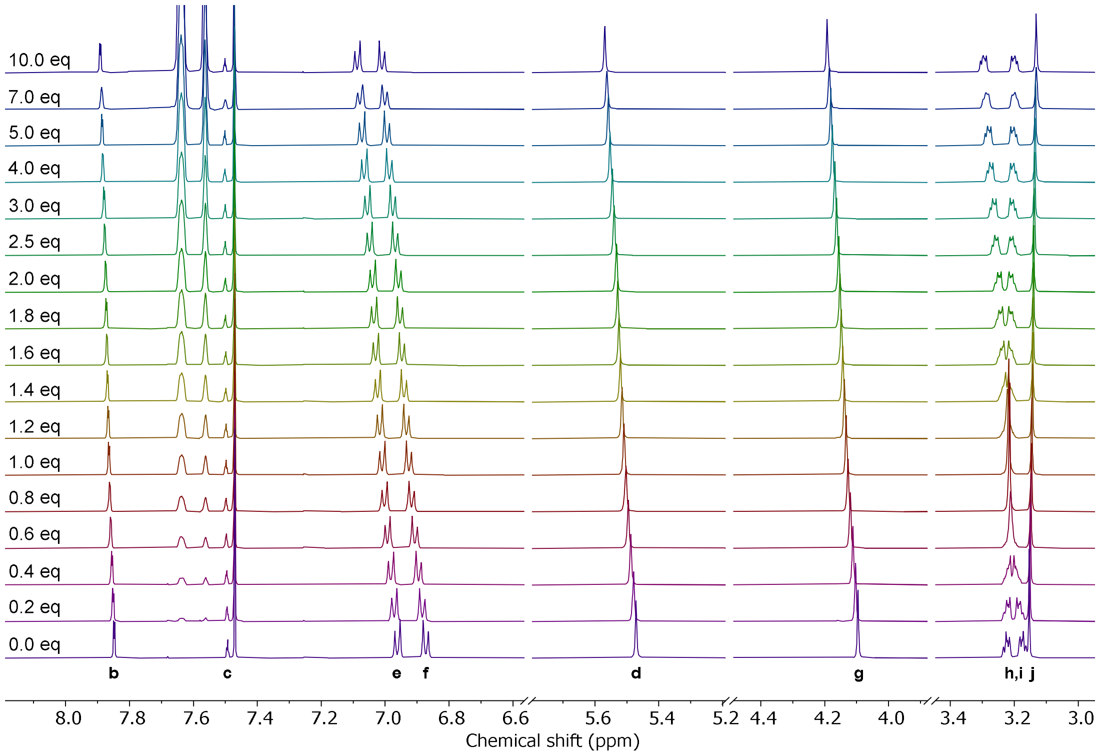

*Figure S32. Truncated ^{1}H NMR titration spectra of [2]catenane **2·XB^{TEG}** upon progressive addition of 10 equivalents NaBAr^{F} (500 MHz, 298 K, 1:1 $CDCl_3/CD_3CN$, [Receptor] = 1.0 mM).*

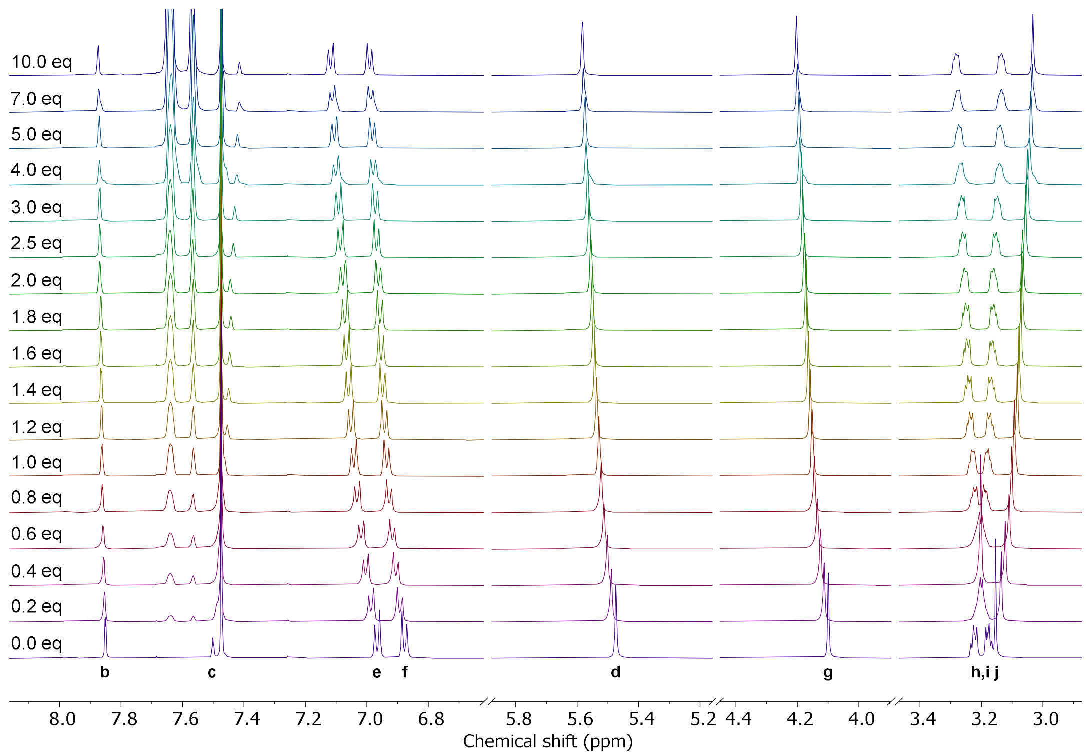

*Figure S33. Truncated ^{1}H NMR titration spectra of [2]catenane **2·XB^{TEG}** upon progressive addition of 10 equivalents KBAr^{F} (500 MHz, 298 K, 1:1 $CDCl_3$/CD_3CN, [Receptor] = 1.0 mM).*

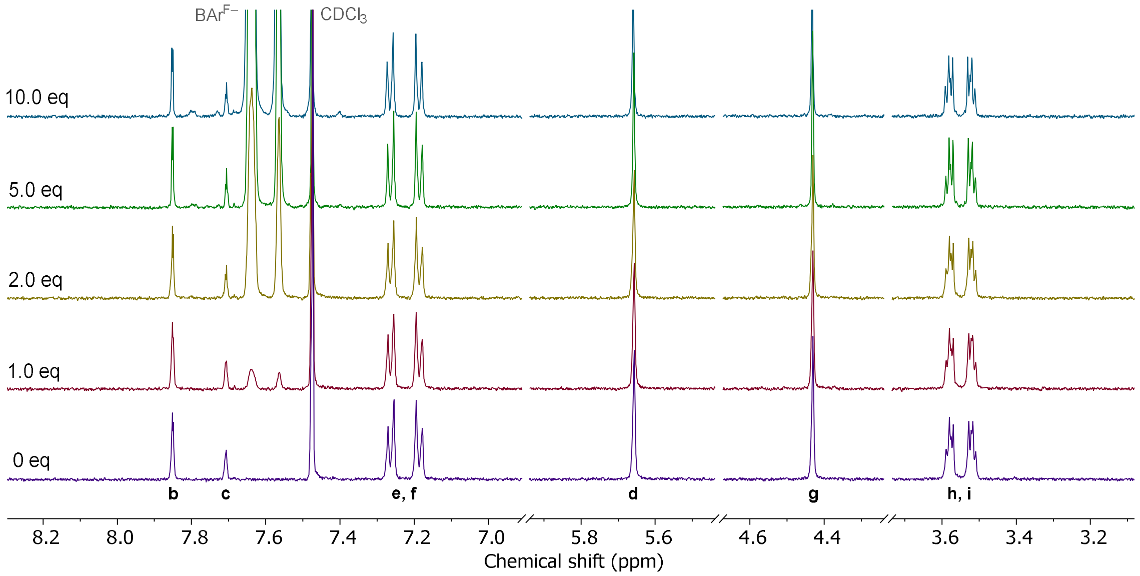

*Figure S 34. Truncated ^{1}H NMR titration spectra of macrocycle **1·XB^{DEG}** upon progressive addition of 10 equivalents NaBAr^{F} (500 MHz, 298 K, 1:1 $CDCl_3$/CD_3CN, [Receptor] = 1.0 mM).*

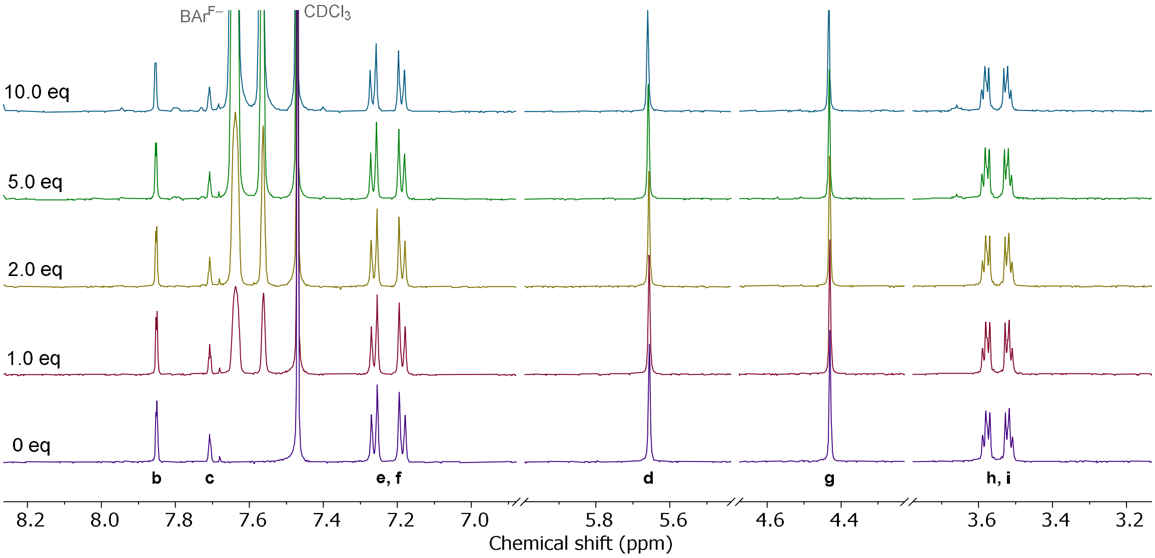

*Figure S35. Truncated ^{1}H NMR titration spectra of macrocycle **1·XB^{DEG}** upon progressive addition of 10 equivalents KBAr^{F} (500 MHz, 298 K, 1:1 $CDCl_3$/CD_3CN, [Receptor] = 1.0 mM).*

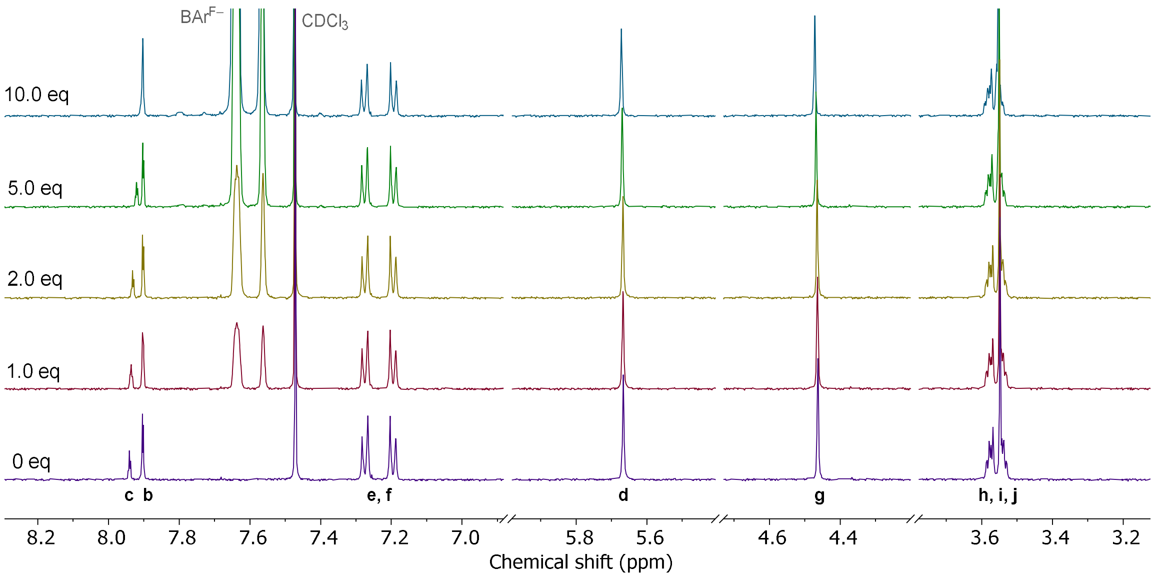

*Figure S 36. Truncated ^{1}H NMR titration spectra of macrocycle **1·XB^{TEG}** upon progressive addition of 10 equivalents NaBAr^{F} (500 MHz, 298 K, 1:1 $CDCl_3$/CD_3CN, [Receptor] = 1.0 mM).*

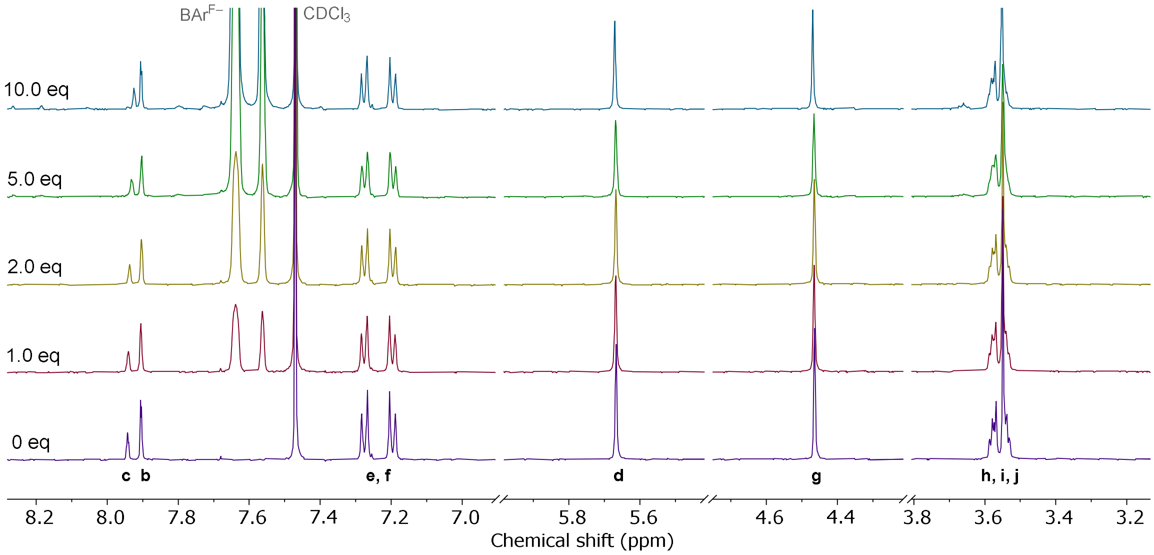

*Figure S37. Truncated ^{1}H NMR titration spectra of macrocycle **1·XB^{TEG}** upon progressive addition of 10 equivalents KBAr^F (500 MHz, 298 K, 1:1 $CDCl_3$/CD_3CN, [Receptor] = 1.0 mM).*

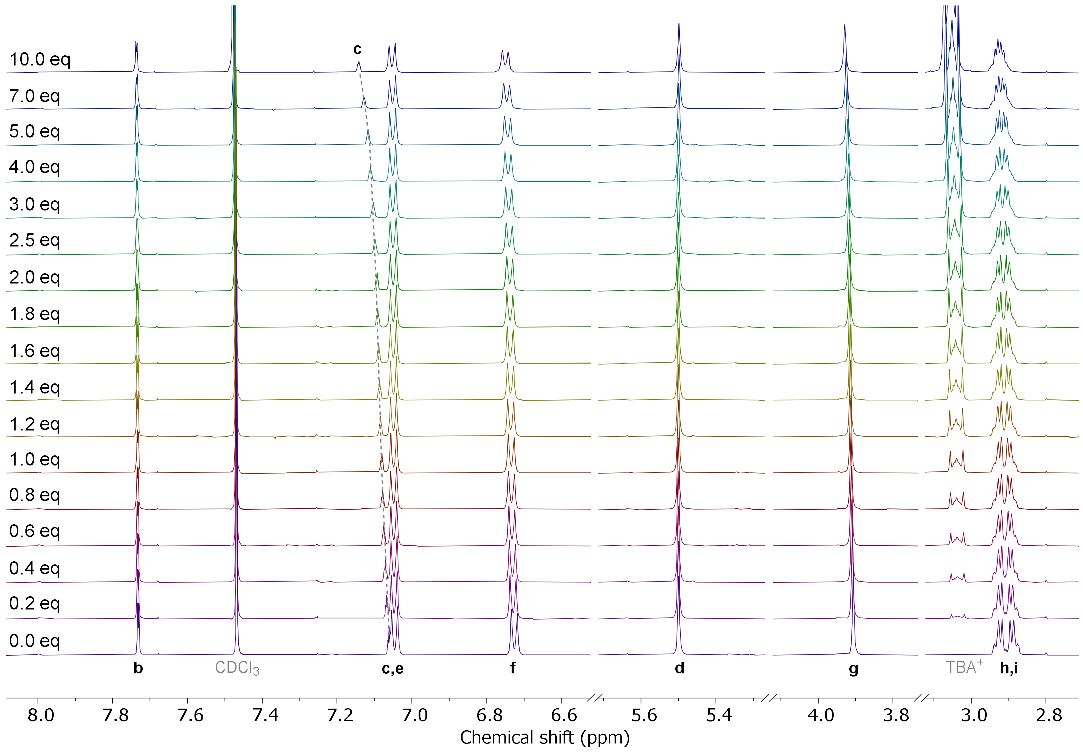

*Figure S38. Truncated ^{1}H NMR titration spectra of [2]catenane **2·XB^{DEG}** upon progressive addition of 10 equivalents TBACl (500 MHz, 298 K, 1:1 $CDCl_3$/CD_3CN, [Receptor] = 1.0 mM).*

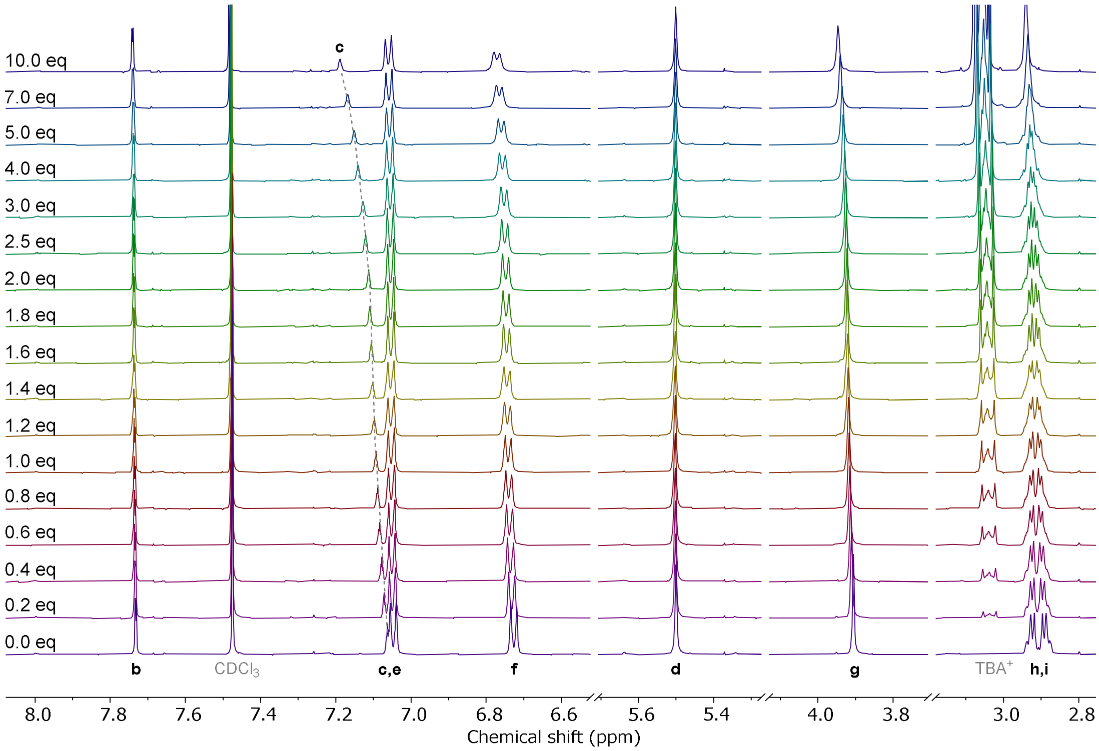

*Figure S39. Truncated ^{1}H NMR titration spectra of [2]catenane **2·XB^{DEG}** upon progressive addition of 10 equivalents TBABr (500 MHz, 298 K, 1:1 $CDCl_3/CD_3CN$, [Receptor] = 1.0 mM).*

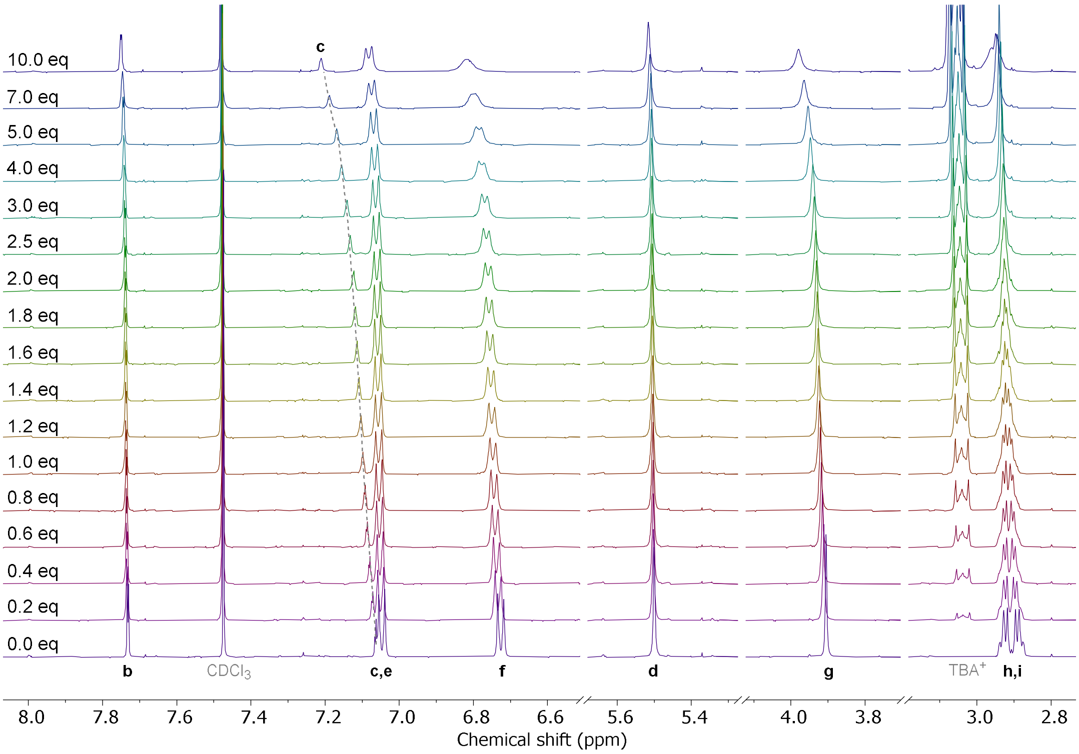

*Figure S40. Truncated ^{1}H NMR titration spectra of [2]catenane **2·XB^{DEG}** upon progressive addition of 10 equivalents TBABr (500 MHz, 298 K, 1:1 $CDCl_3/CD_3CN$, [Receptor] = 1.0 mM).*

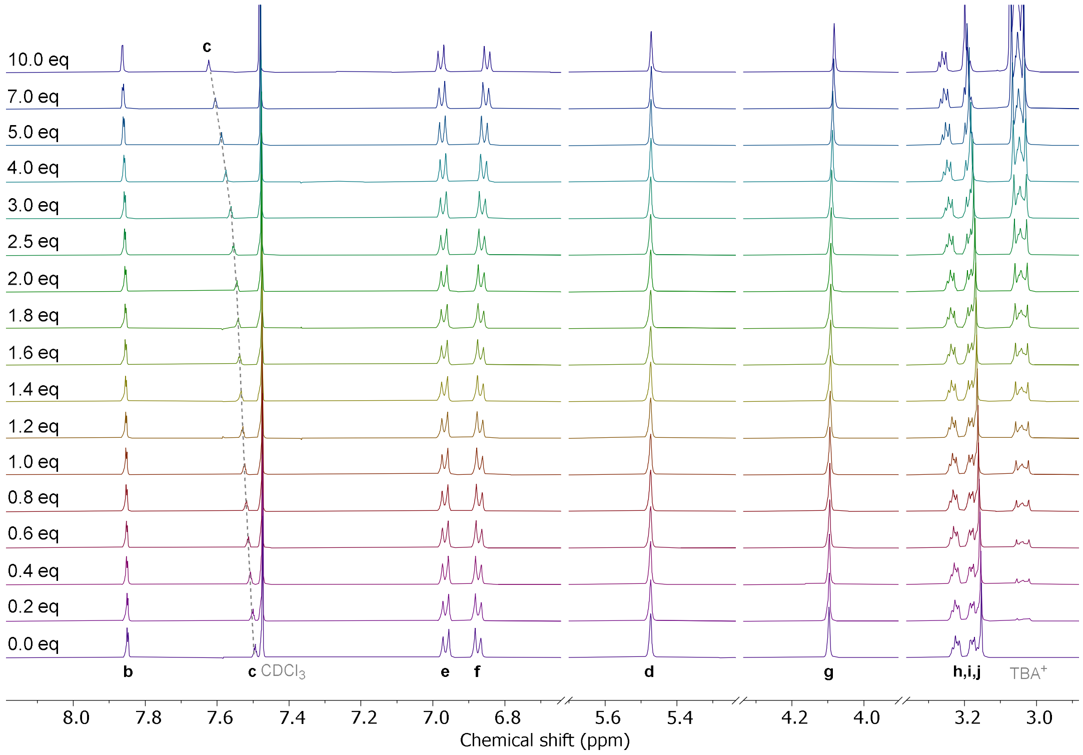

*Figure S41. Truncated ^{1}H NMR titration spectra of [2]catenane **2·XB^{TEG}** upon progressive addition of 10 equivalents TBACl (500 MHz, 298 K, 1:1 $CDCl_3$/CD_3CN, [Receptor] = 1.0 mM).*

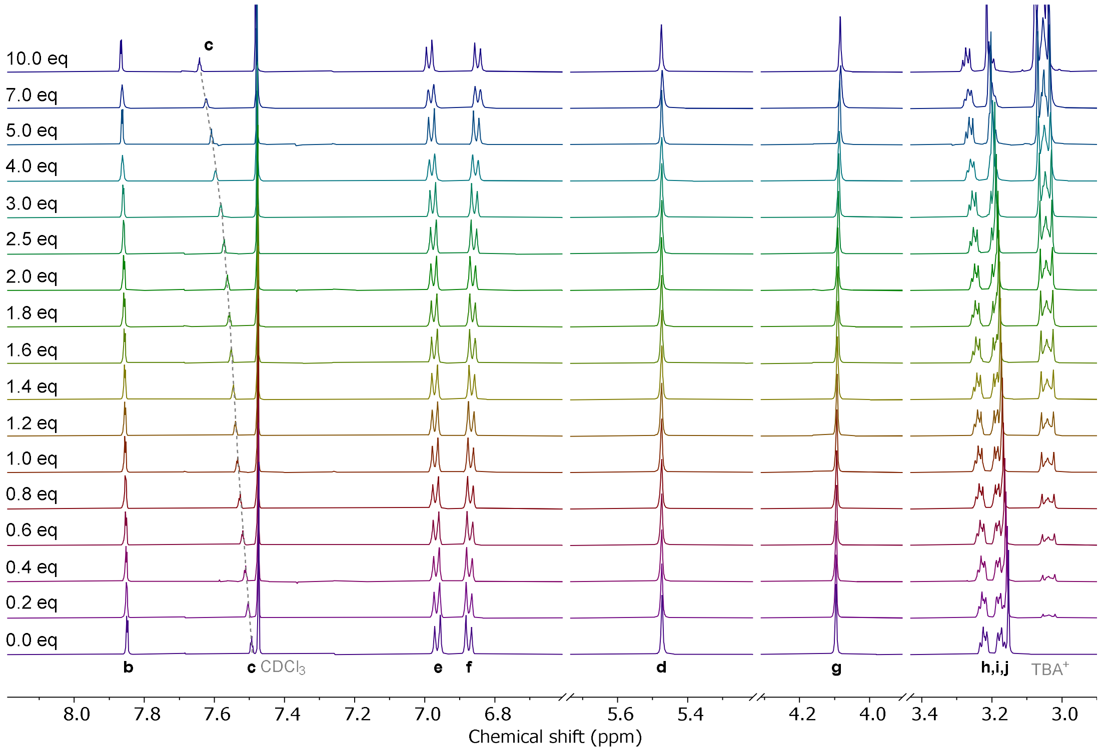

*Figure S42. Truncated ^{1}H NMR titration spectra of [2]catenane **2·XB^{TEG}** upon progressive addition of 10 equivalents TBABr (500 MHz, 298 K, 1:1 $CDCl_3$/CD_3CN, [Receptor] = 1.0 mM).*

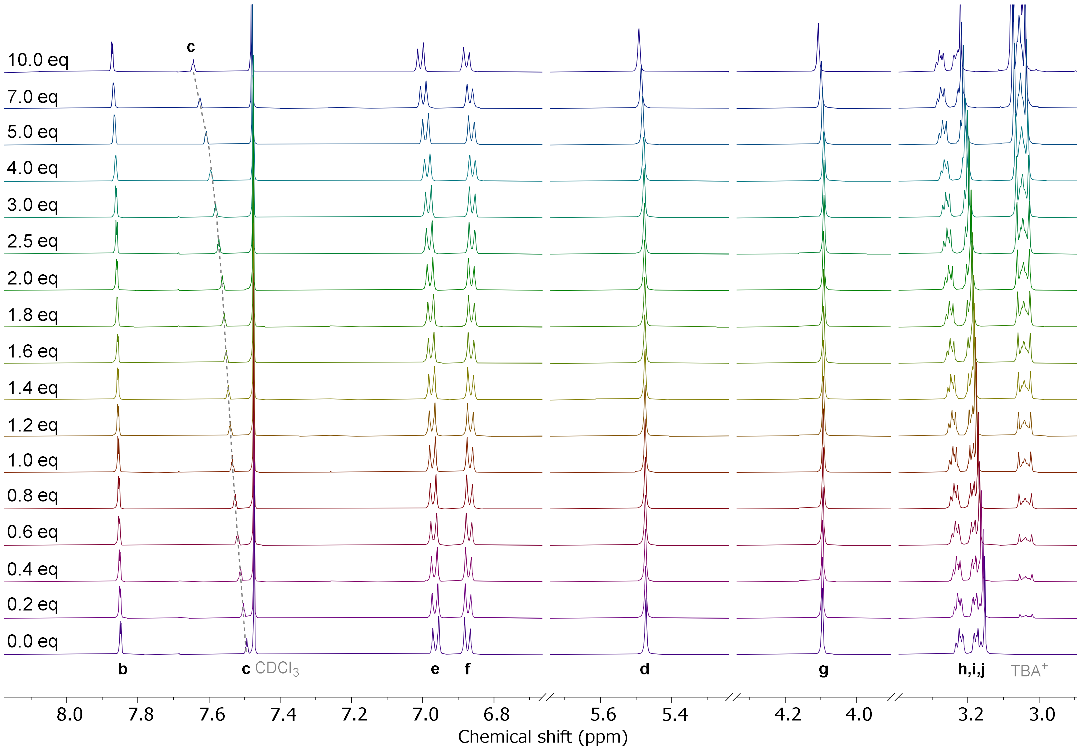

*Figure S43. Truncated ^{1}H NMR titration spectra of [2]catenane **2·XB^{DEG}** upon progressive addition of 10 equivalents TBAI (500 MHz, 298 K, 1:1 $CDCl_3$/CD_3CN, [Receptor] = 1.0 mM).*

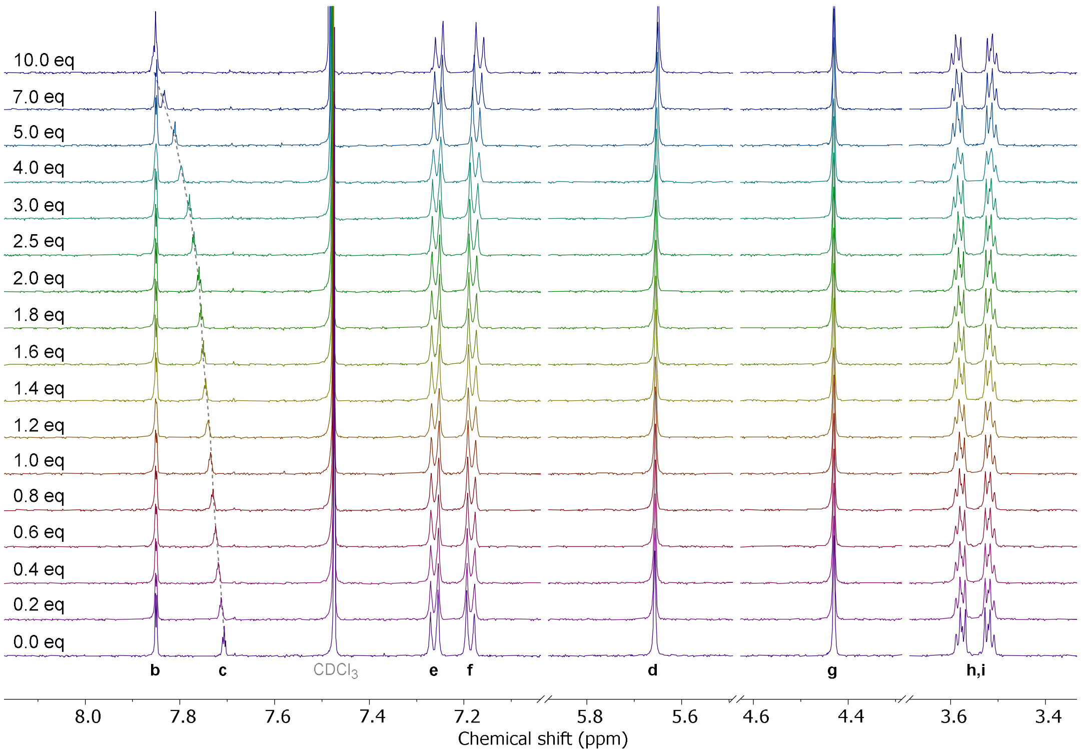

*Figure S44. Truncated ^{1}H NMR titration spectra of macrocycle **1·XB^{DEG}** upon progressive addition of 10 equivalents TBACl (500 MHz, 298 K, 1:1 $CDCl_3$/CD_3CN, [Receptor] = 1.0 mM).*

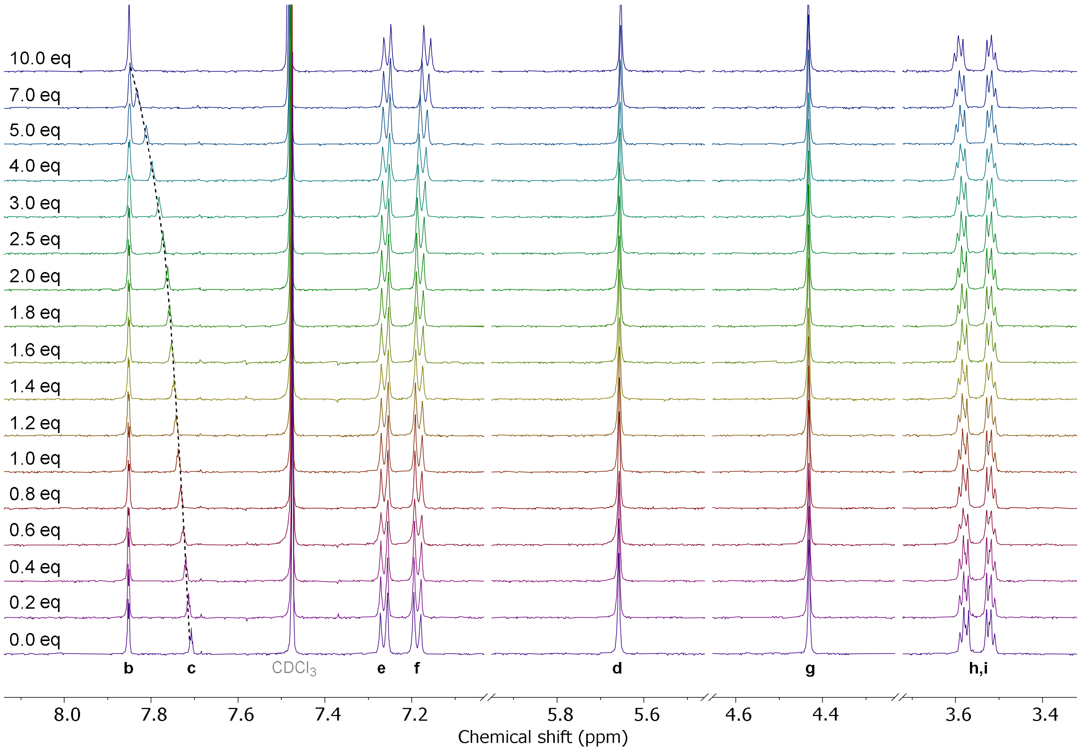

*Figure S45. Truncated ^{1}H NMR titration spectra of macrocycle **1·XB^{DEG}** upon progressive addition of 10 equivalents TBABr (500 MHz, 298 K, 1:1 $CDCl_3$/CD_3CN, [Receptor] = 1.0 mM).*

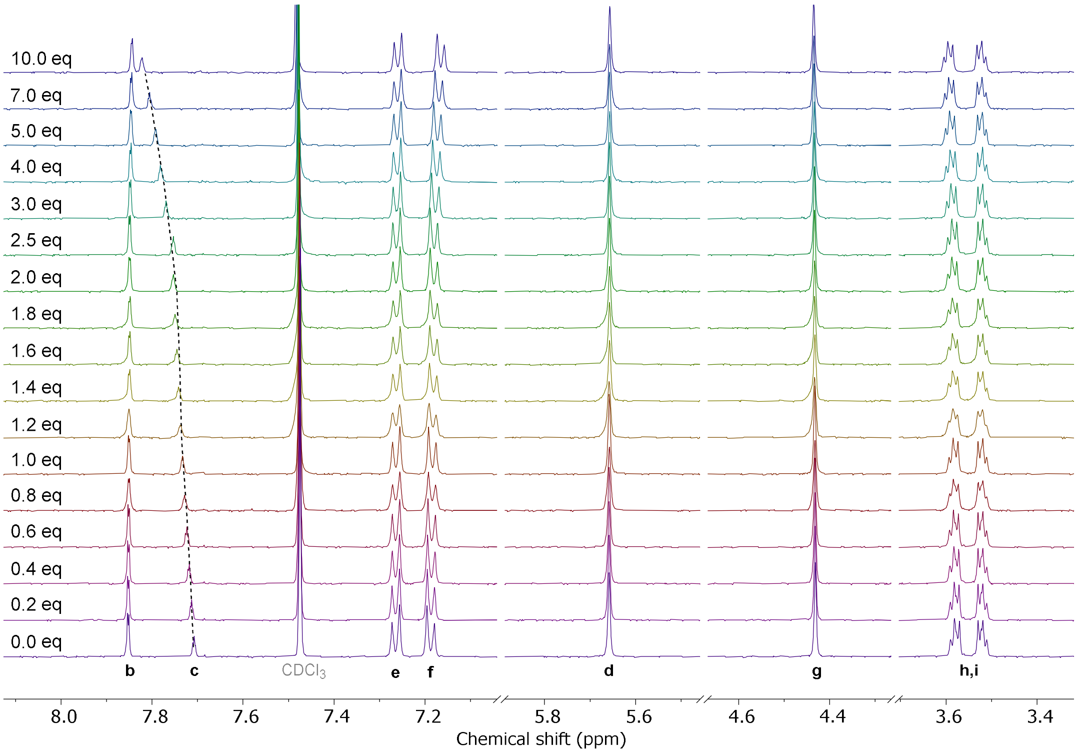

*Figure S46. Truncated ^{1}H NMR titration spectra of macrocycle **1·XB^{DEG}** upon progressive addition of 10 equivalents TBAI (500 MHz, 298 K, 1:1 $CDCl_3$/CD_3CN, [Receptor] = 1.0 mM).*

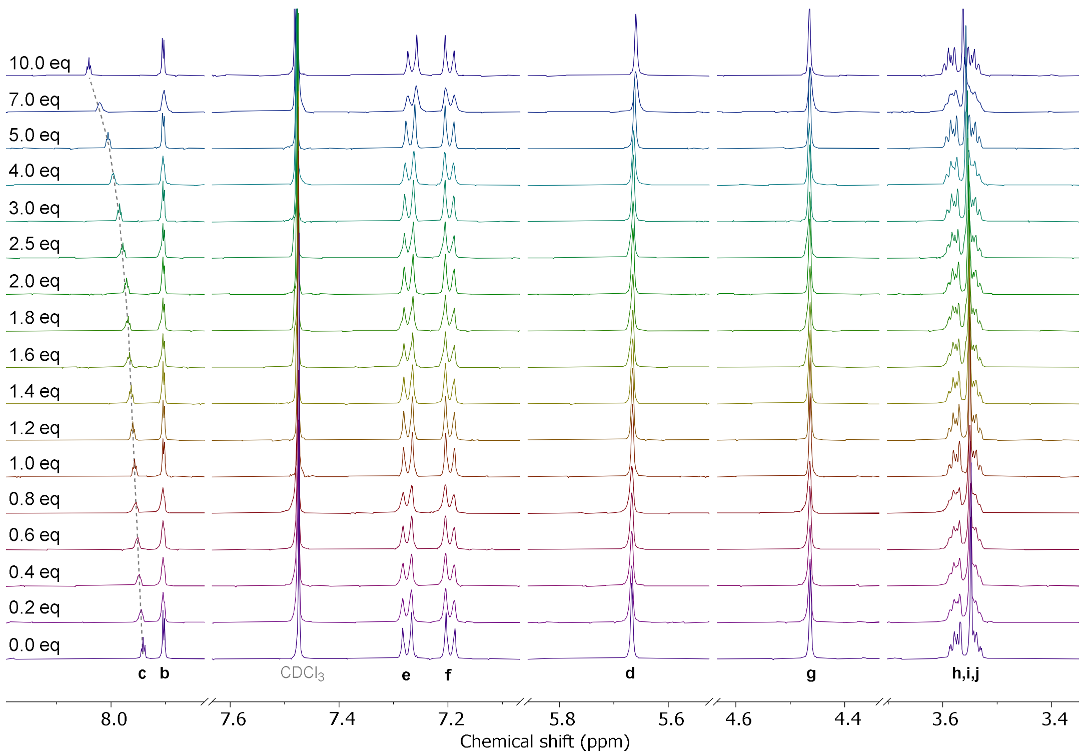

*Figure S47. Truncated ^{1}H NMR titration spectra of macrocycle **1·XB^{TEG}** upon progressive addition of 10 equivalents TBACl (500 MHz, 298 K, 1:1 $CDCl_3$/CD_3CN, [Receptor] = 1.0 mM).*

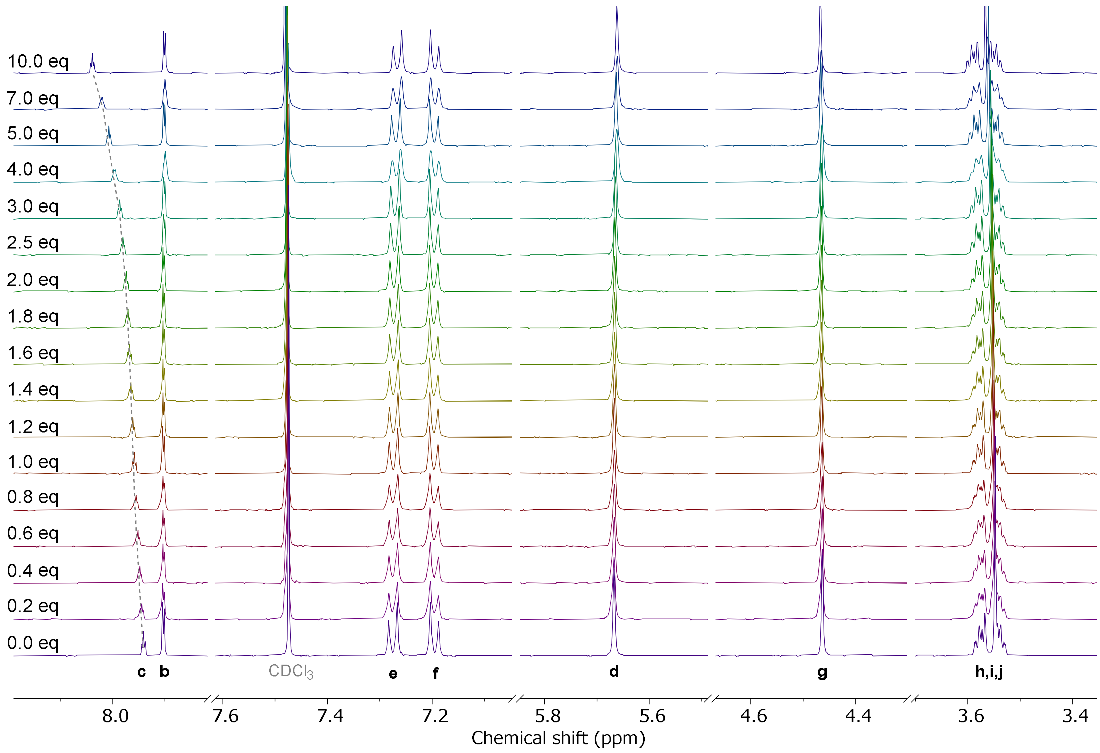

*Figure S48. Truncated ^{1}H NMR titration spectra of macrocycle **1·XB^{TEG}** upon progressive addition of 10 equivalents TBABr (500 MHz, 298 K, 1:1 $CDCl_3$/CD_3CN, [Receptor] = 1.0 mM).*

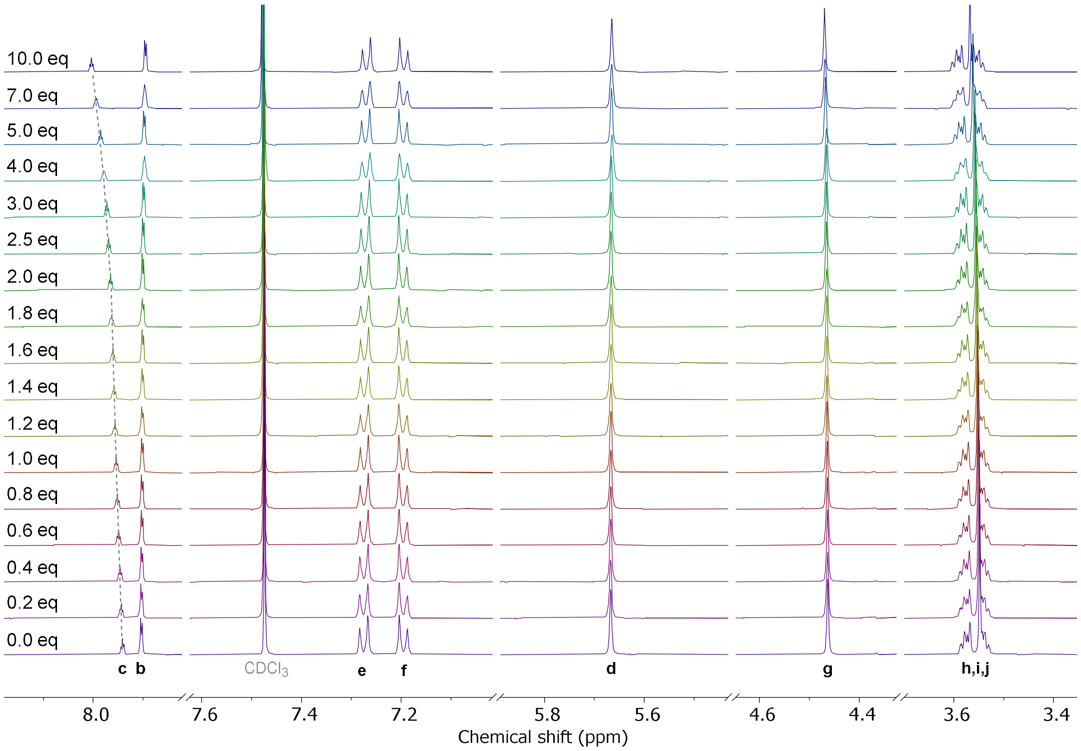

*Figure S49. Truncated ^{1}H NMR titration spectra of macrocycle **1·XB^{TEG}** upon progressive addition of 10 equivalents TBAI (500 MHz, 298 K, 1:1 $CDCl_3/CD_3CN$, [Receptor] = 1.0 mM).*

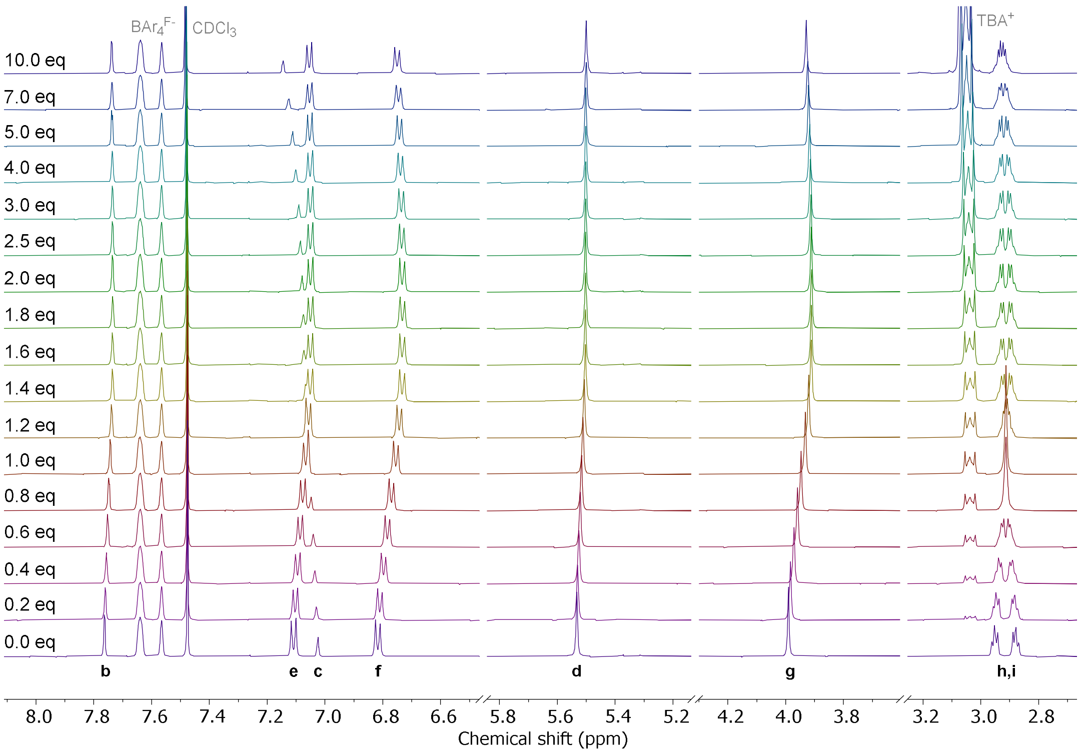

*Figure S50. Truncated ^{1}H NMR titration spectra of [2]catenane **2·XB^{DEG}** upon progressive addition of 10 equivalents TBACl in the presence of 1 equivalent $NaBAr_4^F$ (500 MHz, 298 K, 1:1 $CDCl_3/CD_3CN$, [Receptor] = [$NaBAr_4^F$] = 1.0 mM).*

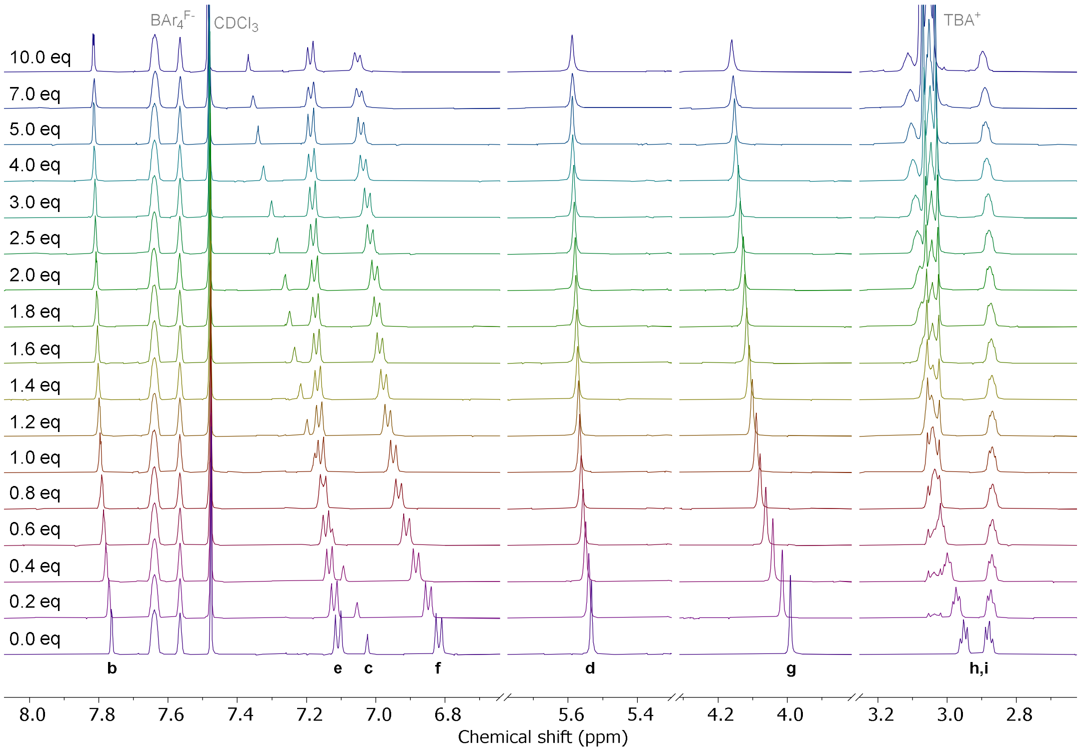

*Figure S51. Truncated ^{1}H NMR titration spectra of [2]catenane **2·XB^{DEG}** upon progressive addition of 10 equivalents TBABr in the presence of 1 equivalent $NaBAr_4^F$ (500 MHz, 298 K, 1:1 $CDCl_3/CD_3CN$, [Receptor] = [$NaBAr_4^F$] = 1.0 mM).*

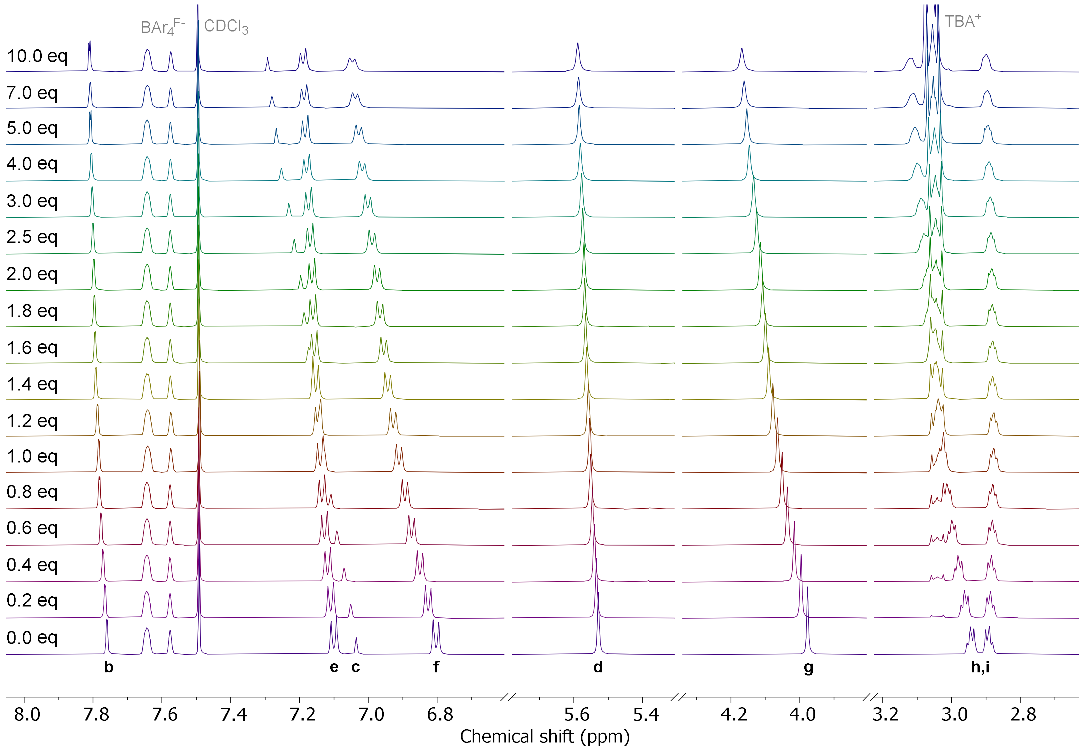

*Figure S52. Truncated ^{1}H NMR titration spectra of [2]catenane **2·XB^{DEG}** upon progressive addition of 10 equivalents TBAI in the presence of 1 equivalent $NaBAr_4^F$ (500 MHz, 298 K, 1:1 $CDCl_3/CD_3CN$, [Receptor] = [$NaBAr_4^F$] = 1.0 mM).*

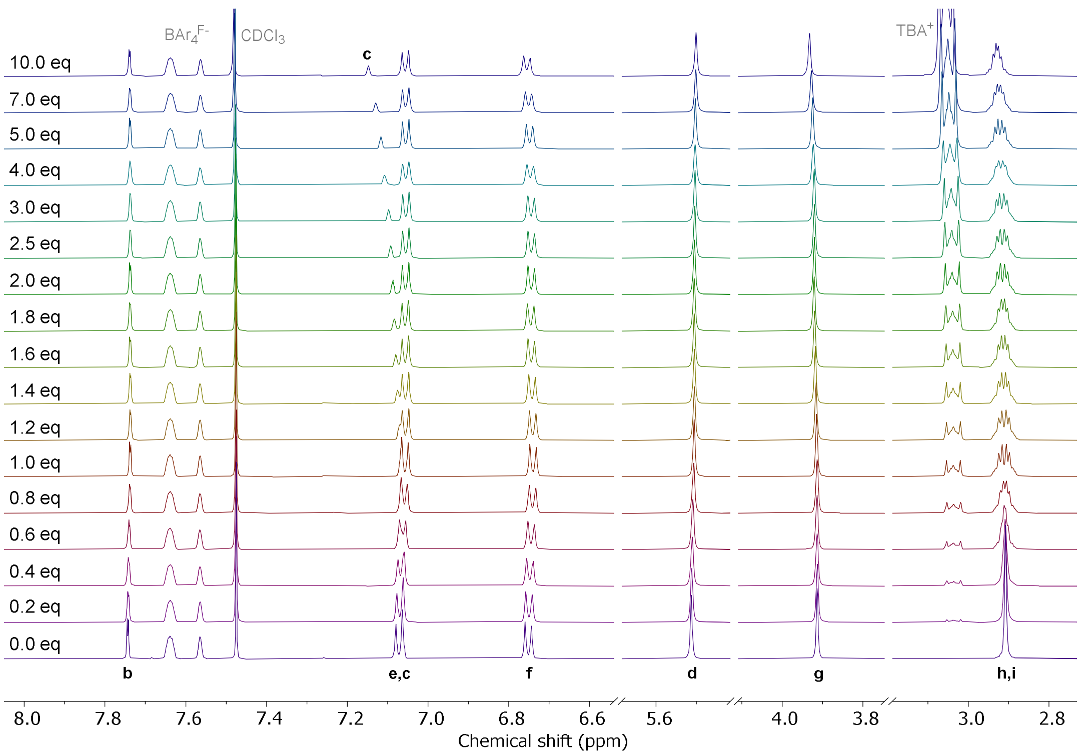

*Figure S 53. Truncated ^{1}H NMR titration spectra of [2]catenane **2·XB^{DEG}** upon progressive addition of 10 equivalents TBACl in the presence of 1 equivalent KBAr$_4^F$ (500 MHz, 298 K, 1:1 $CDCl_3$/CD_3CN, [Receptor] = [KBAr$_4^F$] = 1.0 mM).*

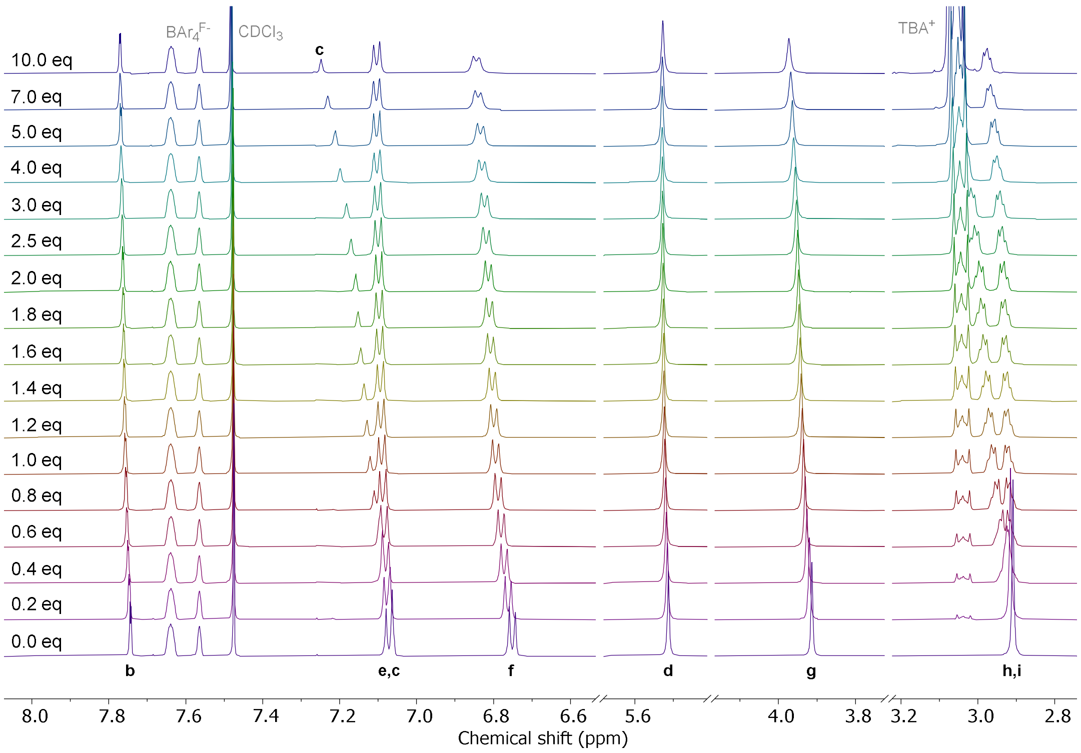

*Figure S54. Truncated ^{1}H NMR titration spectra of [2]catenane **2·XB^{DEG}** upon progressive addition of 10 equivalents TBABr in the presence of 1 equivalent KBAr$_4^F$ (500 MHz, 298 K, 1:1 $CDCl_3$/CD_3CN, [Receptor] = [KBAr$_4^F$] = 1.0 mM).*

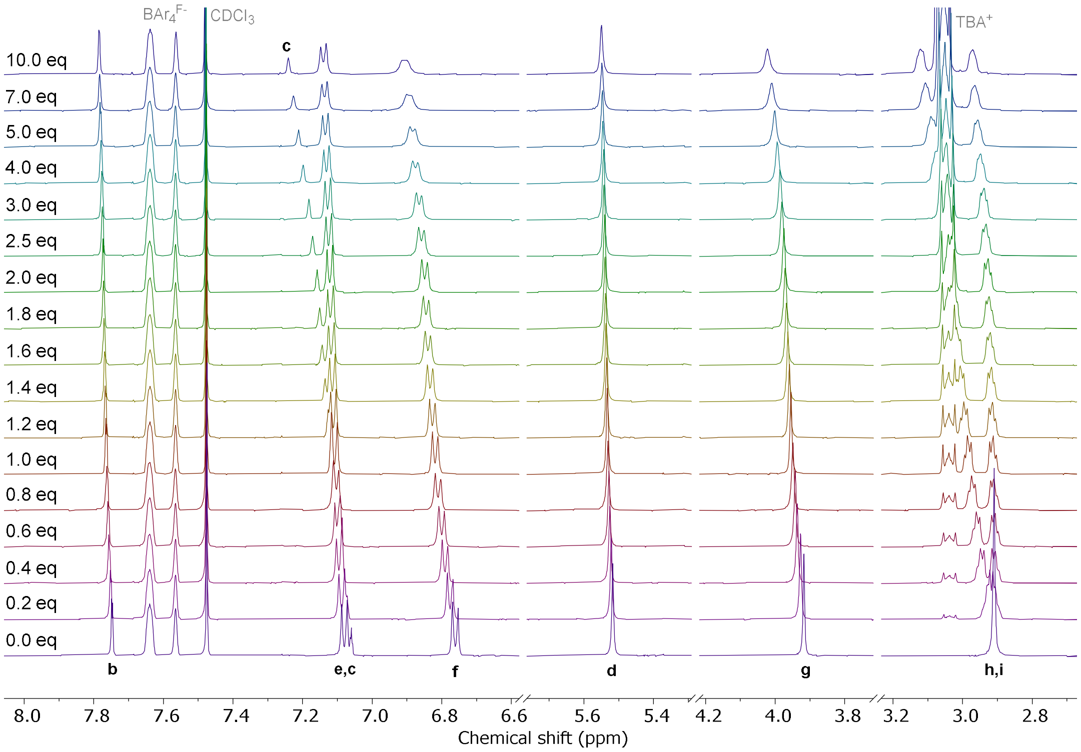

*Figure S55. Truncated ^{1}H NMR titration spectra of [2]catenane **2·XB^{DEG}** upon progressive addition of 10 equivalents TBAI in the presence of 1 equivalent $KBAr_4^F$ (500 MHz, 298 K, 1:1 $CDCl_3/CD_3CN$, [Receptor] = [$KBAr_4^F$] = 1.0 mM).*

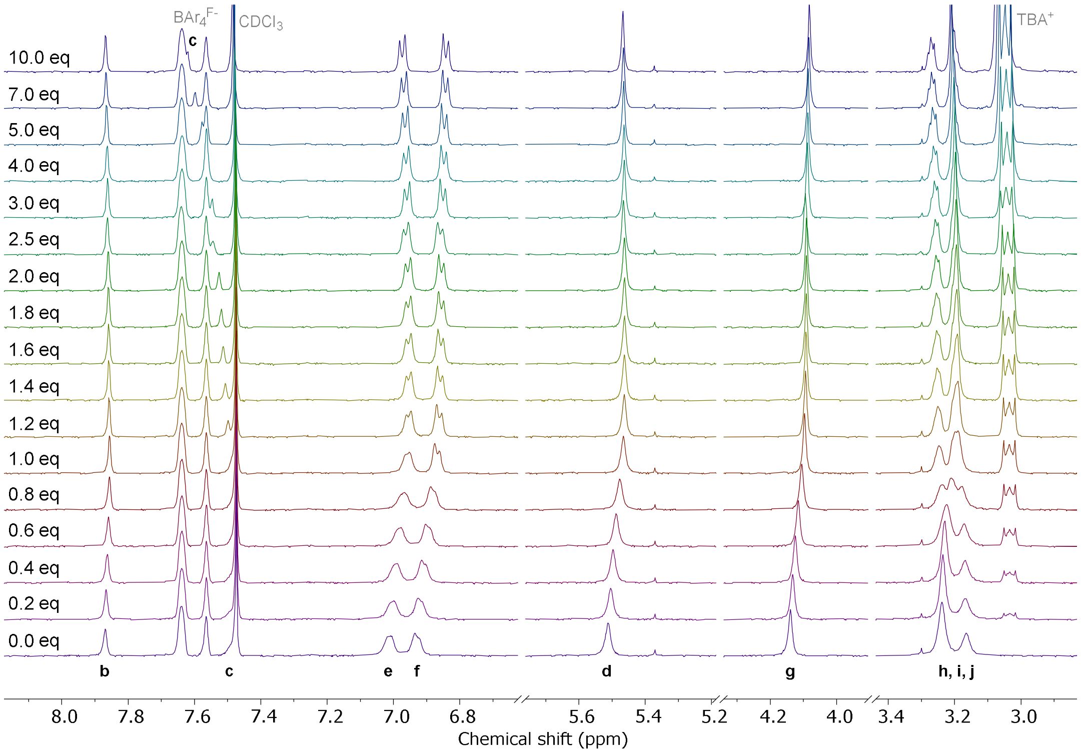

*Figure S56. Truncated ^{1}H NMR titration spectra of [2]catenane **2·XB^{TEG}** upon progressive addition of 10 equivalents TBACl in the presence of 1 equivalent $NaBAr_4^F$ (500 MHz, 298 K, 1:1 $CDCl_3/CD_3CN$, [Receptor] = [$NaBAr_4^F$] = 1.0 mM).*

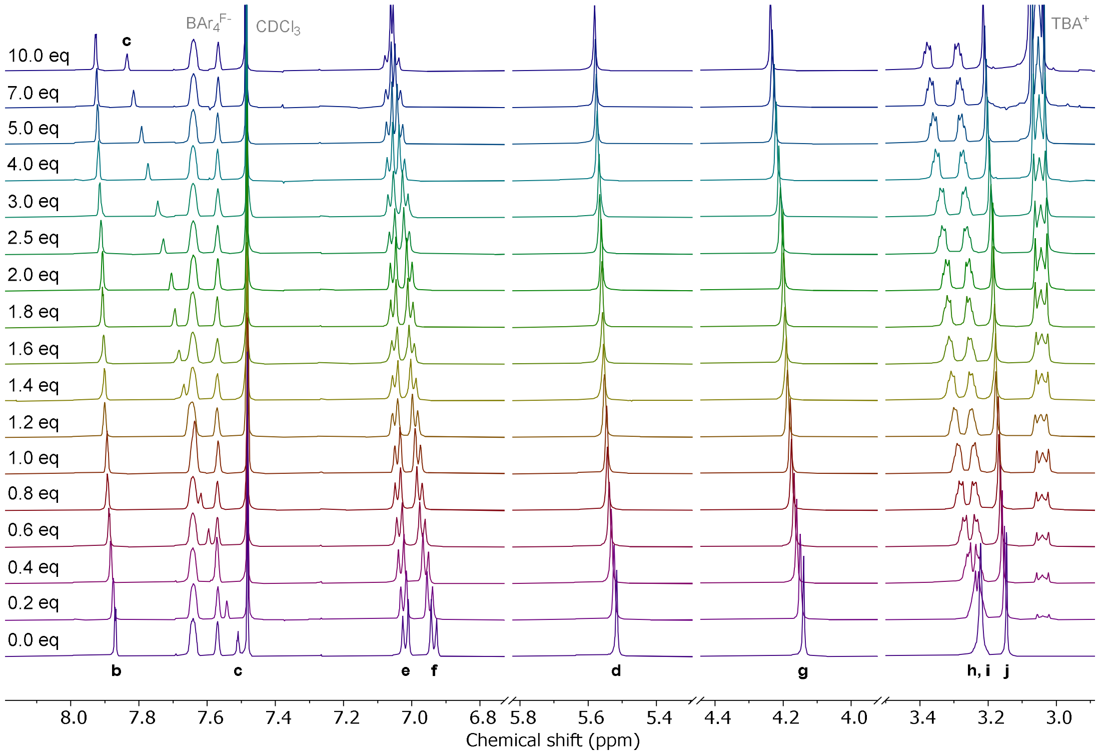

*Figure S57. Truncated ¹H NMR titration spectra of [2]catenane **2·XB^{TEG}** upon progressive addition of 10 equivalents TBABr in the presence of 1 equivalent NaBAr$_4$^{F} (500 MHz, 298 K, 1:1 $CDCl_3$/CD_3CN, [Receptor] = [NaBAr$_4$^{F}] = 1.0 mM).*

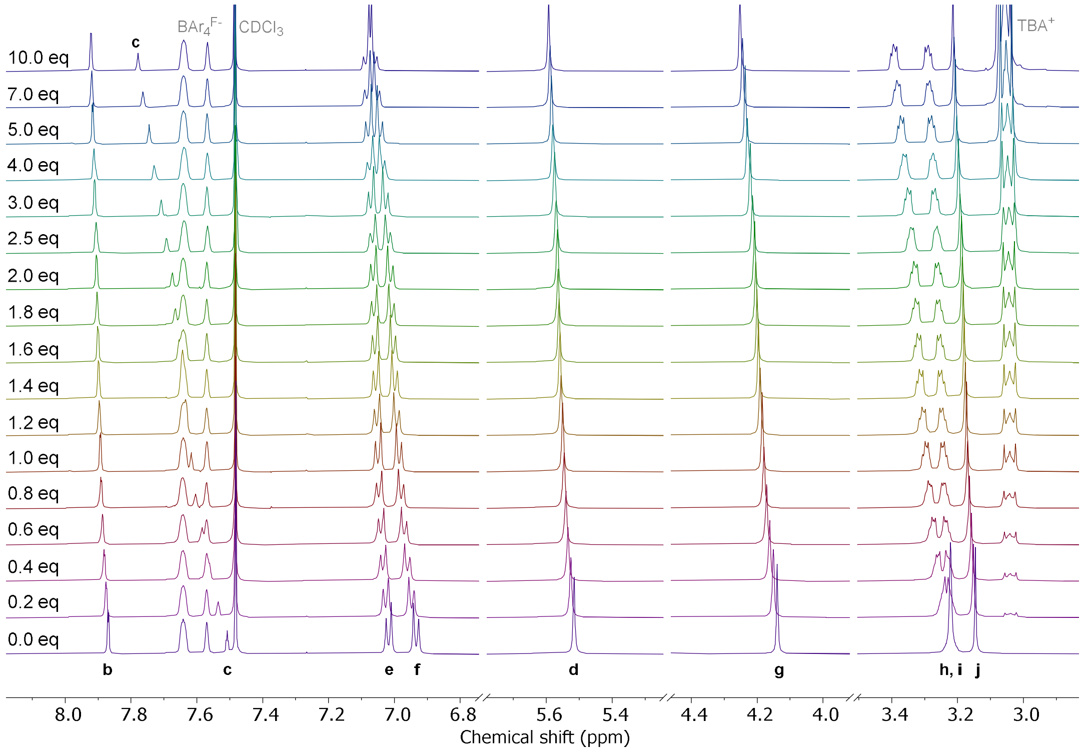

*Figure S58. Truncated ¹H NMR titration spectra of [2]catenane **2·XB^{TEG}** upon progressive addition of 10 equivalents TBAI in the presence of 1 equivalent NaBAr$_4$^{F} (500 MHz, 298 K, 1:1 $CDCl_3$/CD_3CN, [Receptor] = [NaBAr$_4$^{F}] = 1.0 mM).*

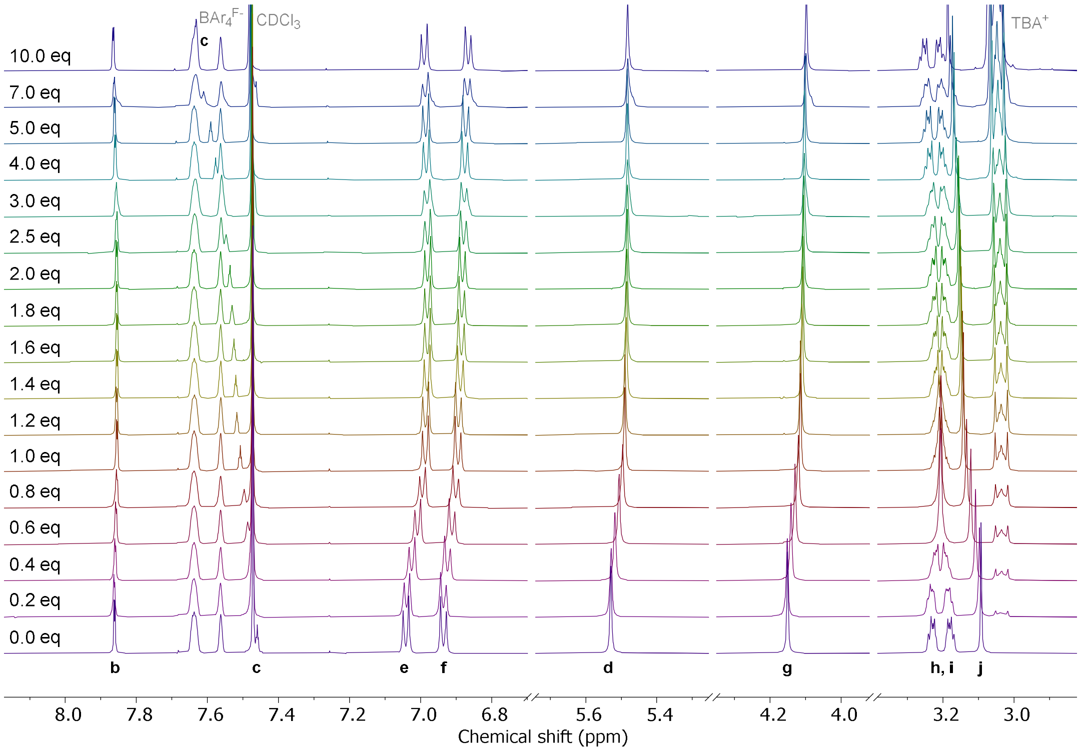

*Figure S59. Truncated ^{1}H NMR titration spectra of [2]catenane **2·XB^{TEG}** upon progressive addition of 10 equivalents TBACl in the presence of 1 equivalent $KBAr_4^F$ (500 MHz, 298 K, 1:1 $CDCl_3/CD_3CN$, [Receptor] = [$KBAr_4^F$] = 1.0 mM).*

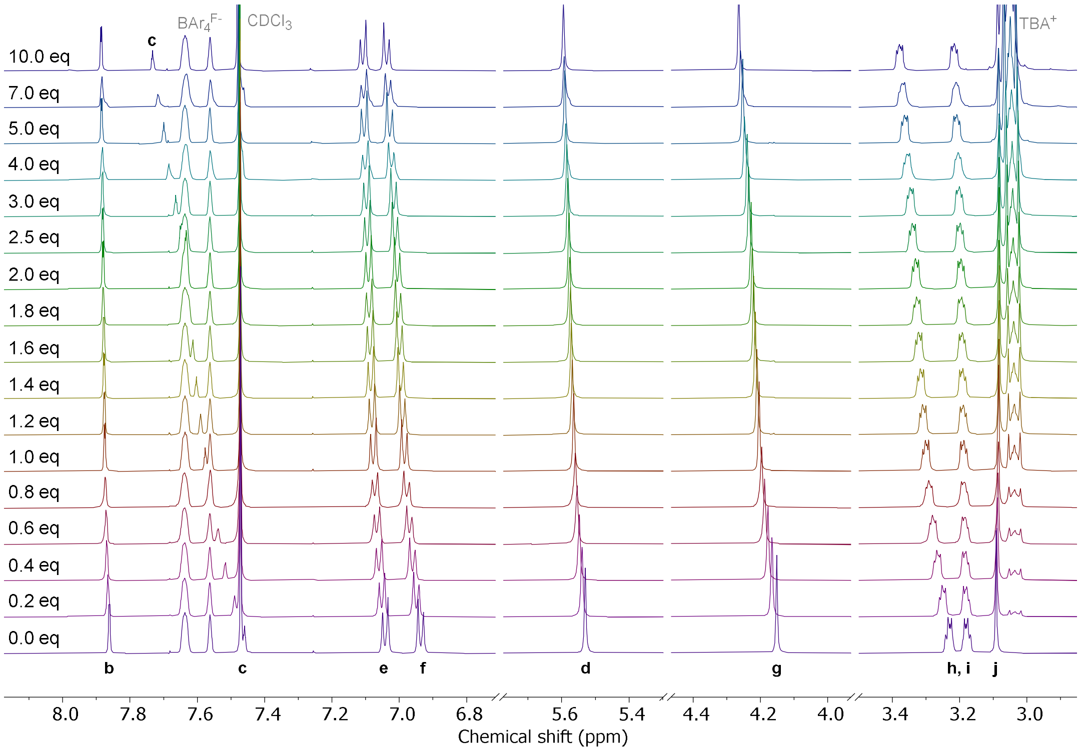

*Figure S60. Truncated ^{1}H NMR titration spectra of [2]catenane **2·XB^{TEG}** upon progressive addition of 10 equivalents TBABr in the presence of 1 equivalent $KBAr_4^F$ (500 MHz, 298 K, 1:1 $CDCl_3/CD_3CN$, [Receptor] = [$KBAr_4^F$] = 1.0 mM).*

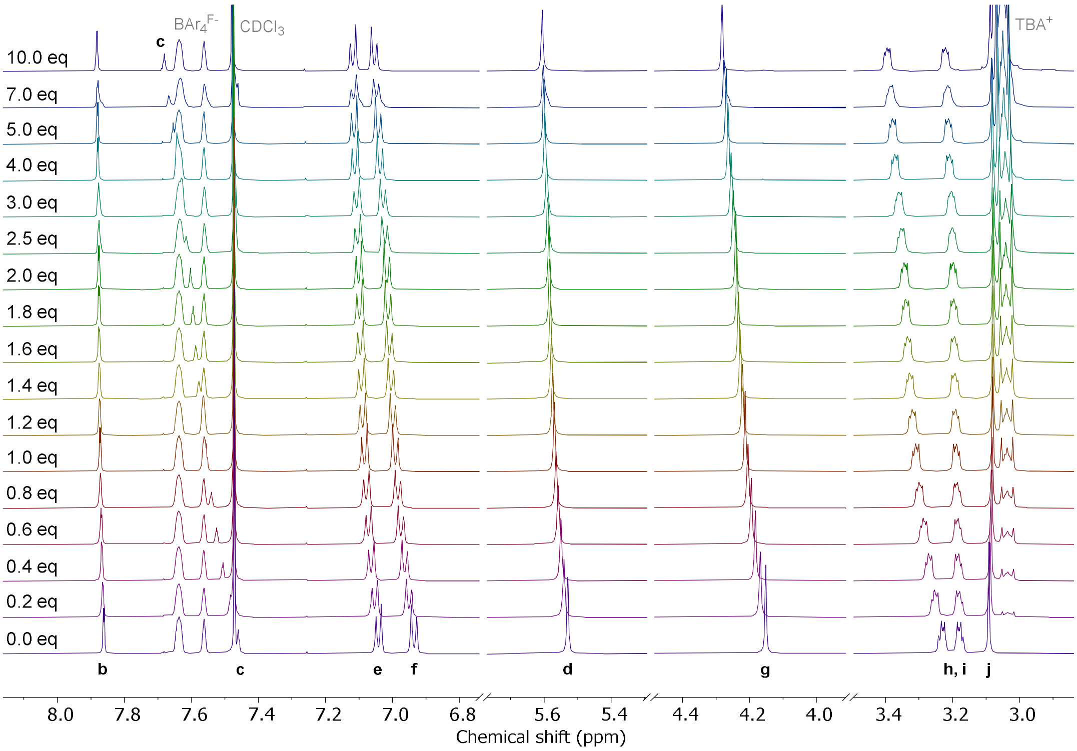

*Figure S61. Truncated ^{1}H NMR titration spectra of [2]catenane **2·XB^{TEG}** upon progressive addition of 10 equivalents TBAI in the presence of 1 equivalent $KBAr_4^F$ (500 MHz, 298 K, 1:1 $CDCl_3/CD_3CN$, [Receptor] = [$KBAr_4^F$] = 1.0 mM).*

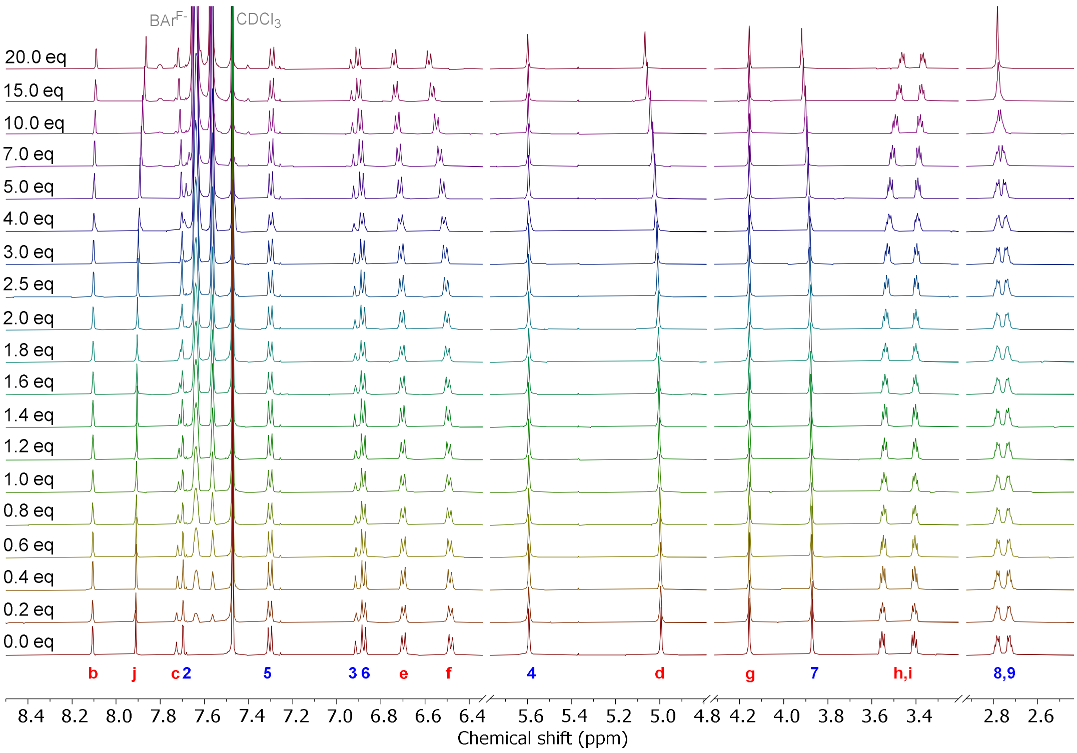

*Figure S62.Truncated ^{1}H NMR titration spectra of [2]catenane **2·HBXB^{DEG}** upon progressive addition of 20 equivalents $NaBAr^F$ (500 MHz, 298 K, 1:1 $CDCl_3/CD_3CN$, [Receptor] = [$KBAr_4^F$] = 1.0 mM).*

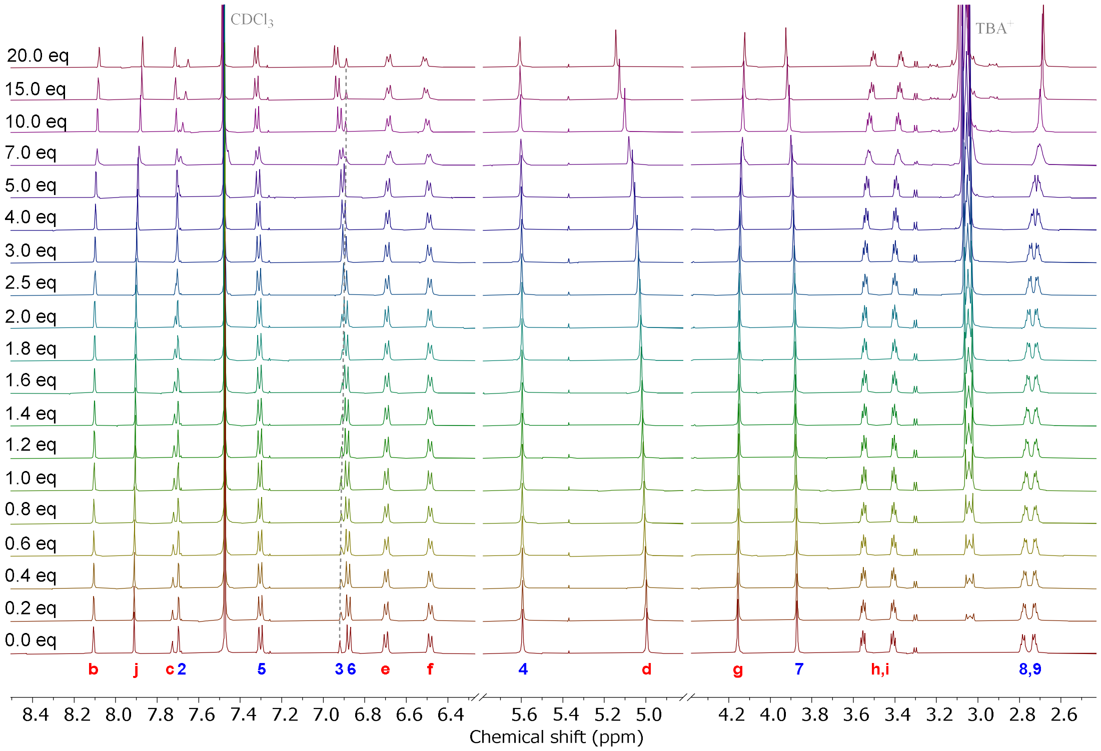

*Figure S63. Truncated ^1H NMR titration spectra of [2]catenane **2·HBXB^{DEG}** upon progressive addition of 20 equivalents TBAI (500 MHz, 298 K, 1:1 $CDCl_3/CD_3CN$, [Receptor] = [$KBAr_4^F$] = 1.0 mM).*

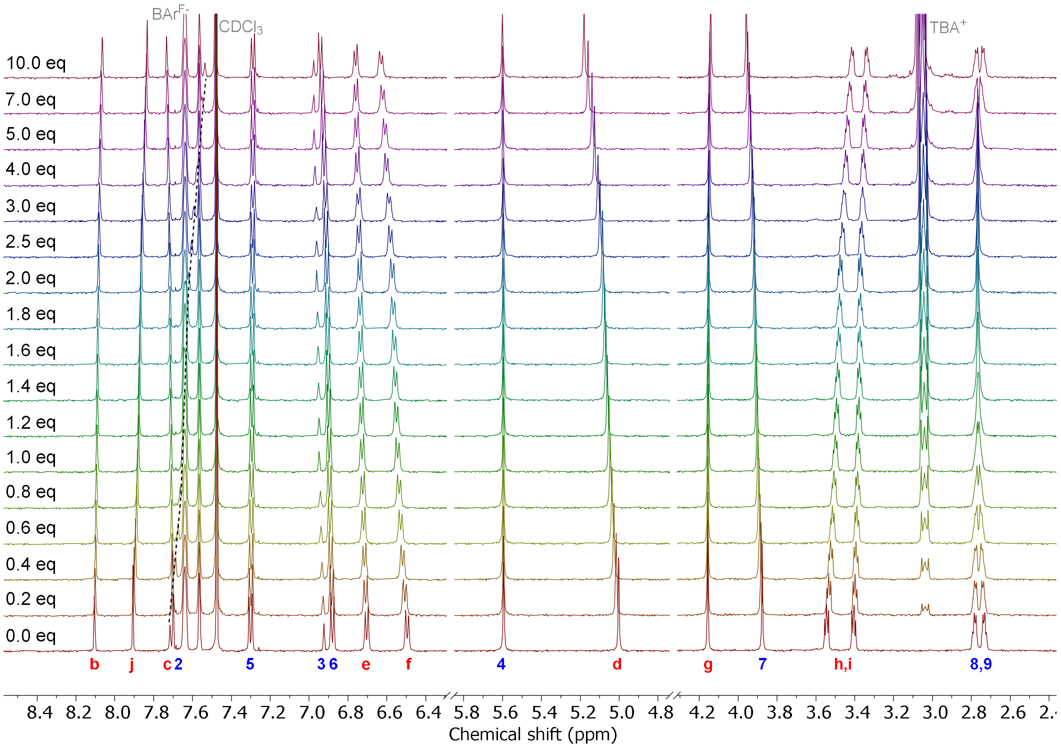

*Figure S64. Truncated ^1H NMR titration spectra of [2]catenane **2·HBXB^{DEG}** upon progressive addition of 10 equivalents TBAI in the presence of 1 equivalent NaBAr^F (500 MHz, 298 K, 1:1 $CDCl_3/CD_3CN$, [Receptor] = [$NaBAr_4^F$] = 1.0 mM).*

Binding isotherms

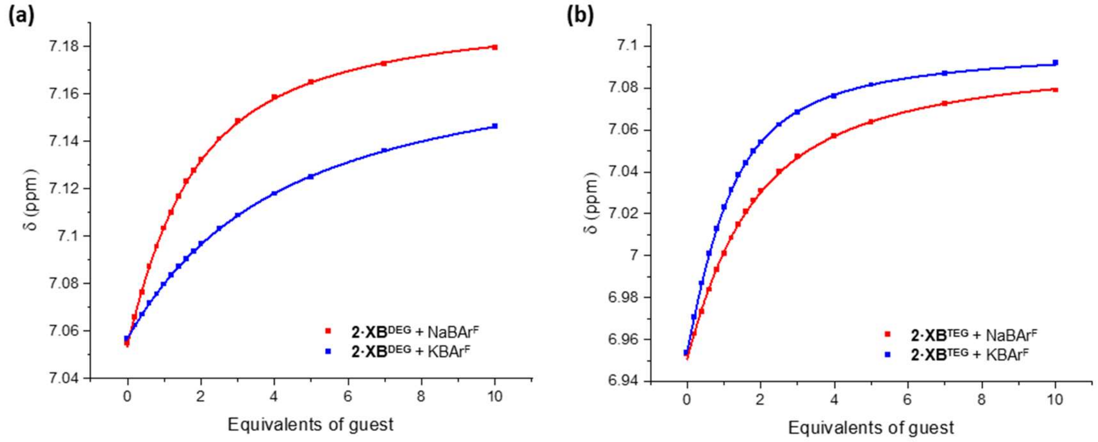

Figure S65. Binding isotherms of [2]catenanes (a) ***2·XB^{DEG}*** *and (b)* ***2·XB^{TEG}****, showing changes in chemical shift of H_e with increasing equivalents of M^IBAr^F salts (M^I = Na^+, K^+). ([Receptor] = 1.0 mM, 500 MHz, 298 K, 1:1 $CDCl_3$:CD_3CN)*

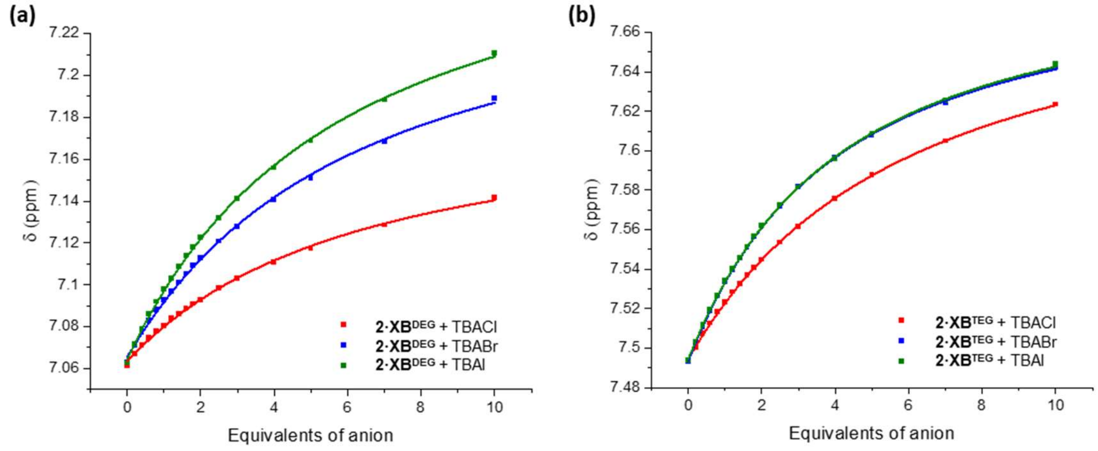

Figure S66. Binding isotherms of [2]catenanes (a) ***2·XB^{DEG}*** *and (b)* ***2·XB^{TEG}****, showing changes in chemical shift of internal benzene proton H_c with increasing equivalents of TBAX salts (X = Cl^-, Br^-, I^-). ([Receptor] = 1.0 mM, 500 MHz, 298 K, 1:1 $CDCl_3$:CD_3CN)*

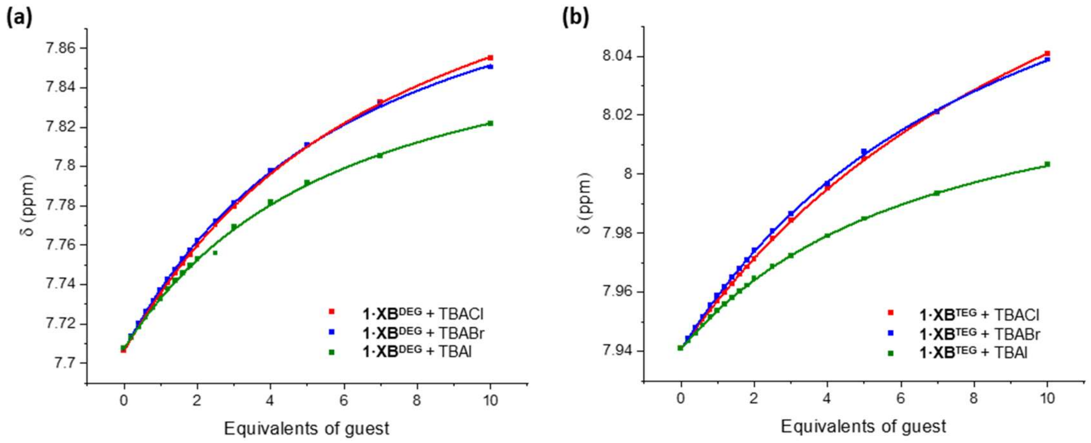

Figure S67. Binding isotherms of macrocycles (a) ***1·XB****^{DEG} and (b)* ***1·XB****^{TEG}, showing changes in chemical shift of internal benzene proton H_c with increasing equivalents of TBAX salts (X = Cl^-, Br^-, I^-). ([Receptor] = 1.0 mM, 500 MHz, 298 K, 1:1 $CDCl_3$:CD_3CN)*

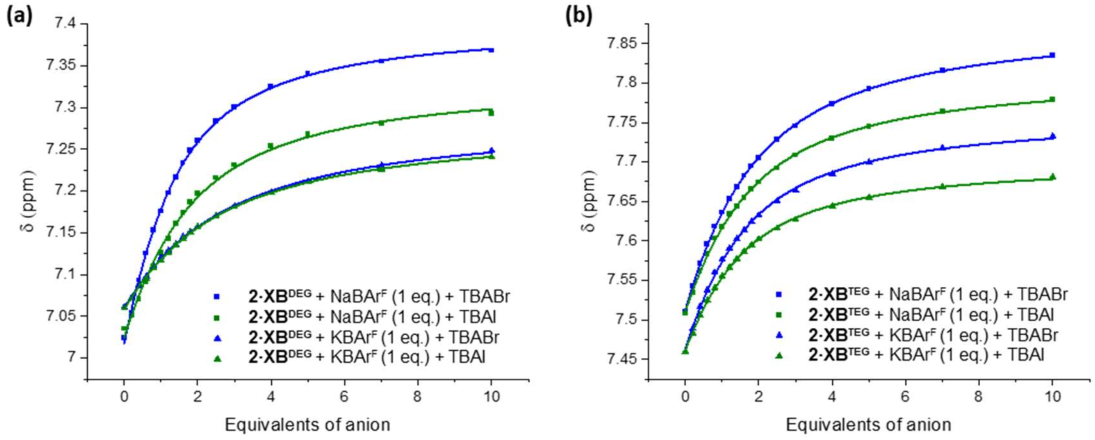

Figure S68. Binding isotherms of [2]catenanes (a) ***2·XB****^{DEG} and (b)* ***2·XB****^{TEG}, showing changes in chemical shift of internal benzene proton H_c with increasing equivalents of TBAX salts (X = Br^-, I^-) in the presence of 1 eq. M^IBAr^F (M^I = Na^+, K^+). ([Receptor] = 1.0 mM, 500 MHz, 298 K, 1:1 $CDCl_3$:CD_3CN)*

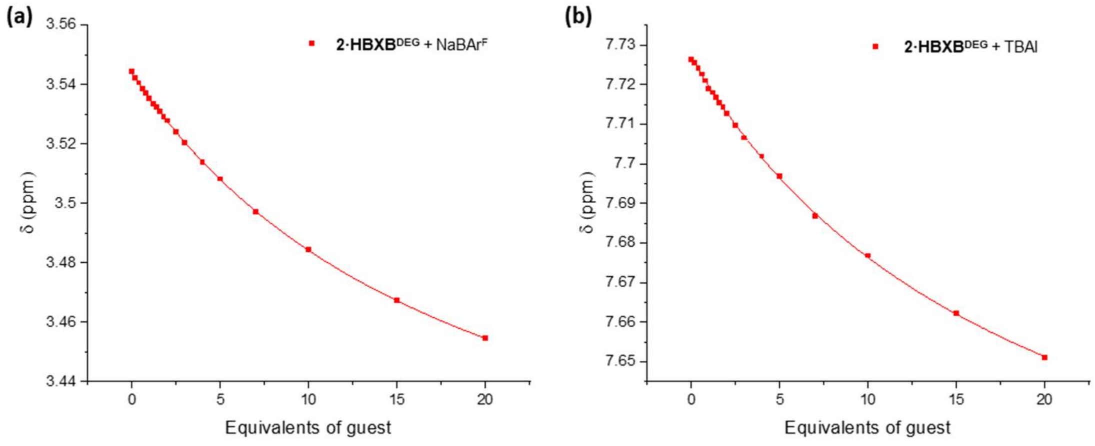

*Figure S69. Binding isotherms of **2·HBXB^{DEG}** showing changes in chemical shift of a) ethylene glycol proton $H_{h/l}$ with increasing equivalents of NaBAr^F; b) internal benzene proton H_3 with increasing equivalents of TBAI. ([Receptor] = 1.0 mM, 500 MHz, 298 K, 1:1 $CDCl_3$:CD_3CN)*

Determination of binding model in ion-pair titrations

Since metal complexation to the [2]catenane produces a monocationic species, there is a possibility of 1:2 host-guest binding that utilises multiple anion binding sites in the [2]catenanes. As such, all binding isotherms were fitted to both the 1:1 host-guest binding model, as well as four variants of the 1:2 host-guest binding model. As shown in **Table S1**, these four variants of 1:2 models differ in the relationships between K_1 and K_2 (step-wise binding constants), and between δ_1 and δ_2 (chemical shifts induced by formation of 1:1 and 1:2 complexes respectively).[4]

Table S1. Key features of the possible binding models involved in the ion-pair titrations of [2]catenanes **2·XB^{DEG}** and **2·XB^{TEG}**[a]

Binding models (Host:Guest)	Relationship between K_1 and K_2	Relationship between δ_1 and δ_2
1:1	Not applicable	Not applicable
1:2 (Full)	$K_1 \neq 4K_2$	$\delta_1 \neq 2\delta_2$
1:2 (Non-cooperative)	$K_1 = 4K_2$	$\delta_1 \neq 2\delta_2$
1:2 (Additive)	$K_1 \neq 4K_2$	$\delta_1 = 2\delta_2$
1:2 (Statistical)	$K_1 = 4K_2$	$\delta_1 = 2\delta_2$

[a]K_1 and K_2 represent the step-wise binding constants. δ_1 and δ_2 represent the chemical shift induced by the formation of 1:1 and 1:2 complexes respectively. [b]Only K_1 and δ_1 are involved in 1:1 binding.

To determine which model best applies to the data, the covariance values of the various fits were compared. Following an approach used by Thordarson and co-workers,[5] a parameter known as the cov_{fit} factor was introduced, which defined as the ratio of cov_{fit} for the simple 1:1 model to cov_{fit} for each of the 1:2 models. In general, the better a model fits the data, the lower the value of cov_{fit} and hence the higher the value of the cov_{fit} factor. However, the number of fitted parameters should also be considered, as complex binding models with a larger number of fitted parameters, such as the 1:2 (Full) model, may appear to fit better to the data and yield an apparently lower cov_{fit}. Therefore, the more complex 1:2 binding models were only considered applicable if they possess a significantly lower cov_{fit} than the simple 1:1 model – that is, cov_{fit} factor > 10. The binding models and corresponding K_1 and K_2 values obtained from this analysis are shown in **Table S2**.

Table S2. Apparent anion association constants (K_a/M^{-1}) for [2]catenanes **2·XB^{DEG}** and **2·XB^{TEG}** in the presence of 1 equiv. M^IBAr^F (M^I = Na, K) determined by ^1H NMR titrations.[a]

Host	Cation	% cat·M^I	Anion	Binding model	K_1	K_2	Cov_{fit} ($\times 10^{-4}$)	cov_{fit} factor
2·XB^{DEG}	Na^+	33	Br^-	1:1	724	-	5.1	1.000
				**1:2 (Full)[b]**	**1900**	**435**	**0.43**	**11.988**
				1:2 (Non-cooperative)	1670	418	0.44	11.801
				1:2 (Additive)	543	< 0	1.3	3.947
				1:2 Statistical)	5.81	1.45	2030	< 0.01
			I^-	1:1	919	-	18	1.000
				**1:2 (Full)[b]**	**3080**	**787**	**1.1**	**15.869**
				1:2 (Non-cooperative)	616	154	3.8	4.750
				1:2 (Additive)	573	< 0	5.0	3.604
				1:2 Statistical)	4.86	1.22	2450	0.007
	K^+	17	Br^-	**1:1[b]**	**432**	**-**	**5.3**	**1.000**
				1:2 (Full)	10600	245	1.8	3.047
				1:2 (Non-cooperative)	692	173	9.3	0.574
				1:2 (Additive)	592	0.462	2.7	1.953
				1:2 Statistical)	3.09	0.772	1280	0.004
			I^-	**1:1[b]**	**487**	**-**	**3.4**	**1.000**
				1:2 (Full)	5190	350	1.6	2.197
				1:2 (Non-cooperative)	984	246	2.5	1.384
				1:2 (Additive)	435	< 0	3.0	1.133
				1:2 Statistical)	3.02	0.754	1470	0.002
2·XB^{TEG}	Na^+	37	Br^-	**1:1[b]**	**745**	**-**	**1.9**	**1.000**
				1:2 (Full)	936	47.0	1.6	1.198
				1:2 (Non-cooperative)	1040	261	7.1	0.266
				1:2 (Additive)	808	0.186	1.6	1.180
				1:2 Statistical)	4.99	1.25	2000	0.001
			I^-	**1:1[b]**	**817**	**-**	**5.3**	**1.000**
				1:2 (Full)	1280	82.5	4.1	1.307
				1:2 (Non-cooperative)	1120	279	13	0.395
				1:2 (Additive)	953	0.284	4.2	1.261
				1:2 Statistical)	4.12	1.03	2130	0.002
	K^+	48	Br^-	**1:1[b]**	**983**	**-**	**5.2**	**1.000**
				1:2 (Full)	2010	132	2.4	2.191
				1:2 (Non-cooperative)	1337	334	17	0.306
				1:2 (Additive)	1223	0.374	2.7	1.936
				1:2 Statistical)	4.01	1.00	2390	0.002
			I^-	**1:1[b]**	**1050**	**-**	**3.0**	**1.000**
				1:2 (Full)	1680	87.4	0.80	3.769
				1:2 (Non-cooperative)	1450	362	14	0.212
				1:2 (Additive)	1270	0.271	0.88	3.415
				1:2 Statistical)	3.23	0.808	2490	0.001

[a]*Ka* values calculated using Bindfit. Errors (±) are all < 5% unless otherwise stated. All anions added as TBA^+ salts. Solvent = 1:1 CDCl3/CD3CN. *T* = 298 K. [Receptor] = 1.0 mM. [b]Most appropriate binding model determined from cov_{fit} analysis.

Solid-liquid extraction experiments

General Procedure

The capability of the receptors to extract solid alkali metal salts into organic solvent was investigated through a series of solid-liquid extraction (SLE) experiments. In a typical experiment, an excess of a solid alkali metal salt (MCl, MBr or MI, where M = Na, K) was added to 1.0 mM a solution of the receptor in $CDCl_3$ (700 µL) and the mixture was vigorously sonicated for 20 min. The excess salt was subsequently removed by filtration through a syringe filter. A ^{1}H NMR spectrum of the filtrate was collected using a Bruker AVIII 500 MHz spectrometer at 298 K.

^{1}H NMR spectra of the receptors before and after treatment with alkali halide salts

When [2]catenane **2·XB^{DEG}** was treated with NaX (X = Cl$^-$, Br$^-$, I$^-$, ClO_4^-), no extraction of NaCl was observed; NaBr was partially extracted and in slow exchange between the ion-pair bound and uncomplexed receptors; NaI was fully extracted with the receptor in solution existing exclusively as the ion-pair bound complex (see **Figure 6** in main text). To ascertain that the NaI-bound [2]catenane was fully complexed and in slow exchange, 0.25 equivalents of **10** was added to the post-extraction NMR sample and the ^{1}H NMR spectrum was re-collected. Pleasingly, a second set of peaks that approximately corresponds to the unbound [2]catenane and integrates to 25% of the NaI-bound [2]catenane peaks was observed (**Figure S64**).

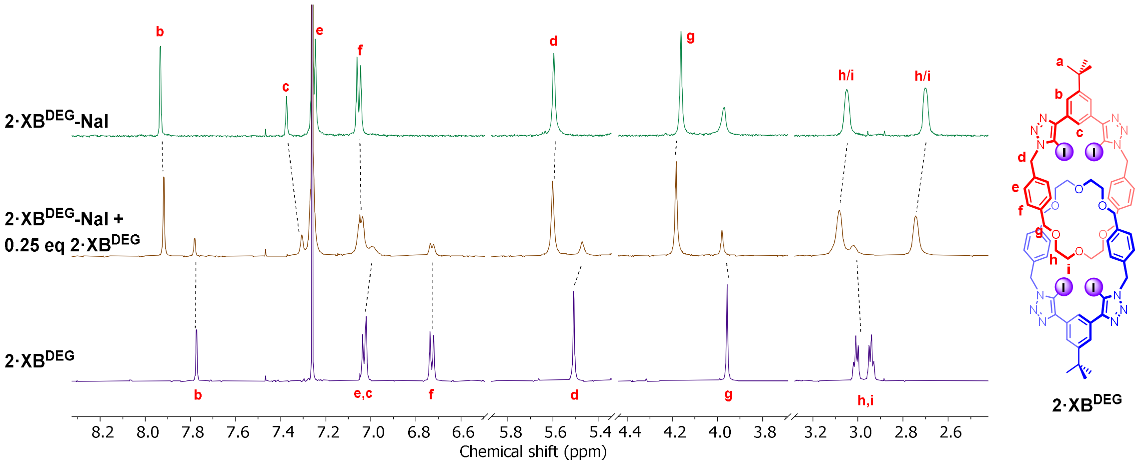

*Figure S70. Overlaid ^{1}H NMR spectra of free **2·XB^{DEG}** (bottom), the fully bound **2·XB^{DEG}**-NaI complex formed post-SLE with NaI (top) and a 4:1 mixture of **2·XB^{DEG}**-NaI and free **2·XB^{DEG}** (middle) (500 MHz, 298 K, CDCl3).*

When **2·XB^{DEG}** was treated with KX (X = Cl$^-$, Br$^-$, I$^-$), no extraction of KCl or KBr was observed. This was attributed to the weaker affinity of **2·XB^{DEG}** for the larger potassium cation. A poorly soluble complex was formed upon treatment of **2·XB^{DEG}** with KI, as seen by the severe broadening of peaks and reduction in intensity. Nonetheless, splitting of the ethylene glycol protons H_h and H_i, as well as downfield shifts of the resonances near the anion binding site, indicates the formation of the KI-bound [2]catenane complex (**Figure S65**).

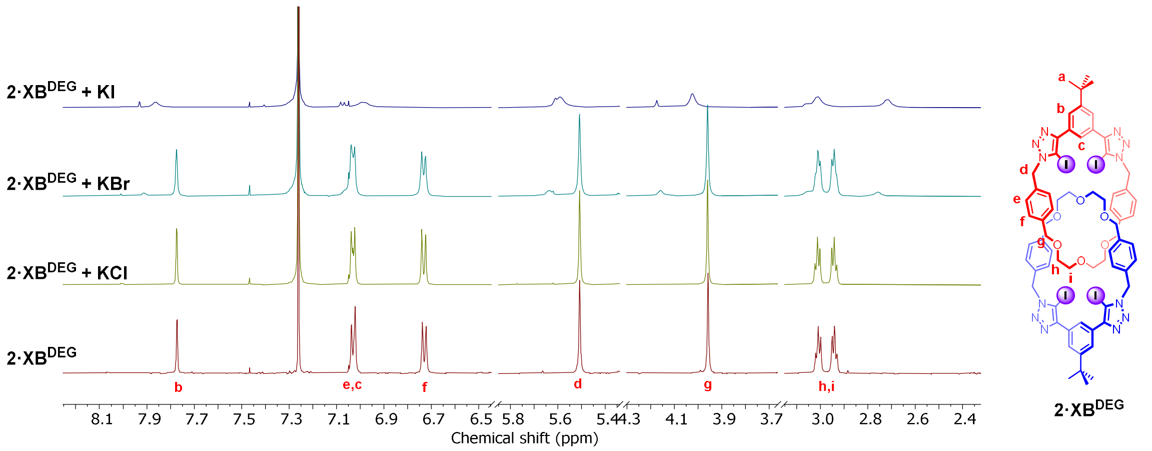

*Figure S71. Pre- and post-extraction ^{1}H NMR spectra of **2·XB^{DEG}** with excess solid KCl, KBr and KI (500 MHz, 298 K, CDCl3).*

Analogous SLE experiments of **2·XB^{TEG}** with NaX (X = Cl$^-$, Br$^-$, I$^-$) resulted in no extraction of NaCl. Severe peak broadening and small chemical shift perturbations were observed upon treatment of **2·XB^{TEG}** with NaBr, suggesting partial extraction. The reduced peak intensities may be due to the poor solubility of the NaBr-bound [2]catenane complex. No [2]catenane peaks were seen in the post-extraction spectrum of NaI, suggesting complete precipitation of the [2]catenane-NaI complex (**Figure S66**). The residue from the syringe filter was washed with DMSO-d_6 and the resulting ^{1}H NMR spectrum confirmed the presence of **2·XB^{TEG}** in the precipitate (**Figure S67**).

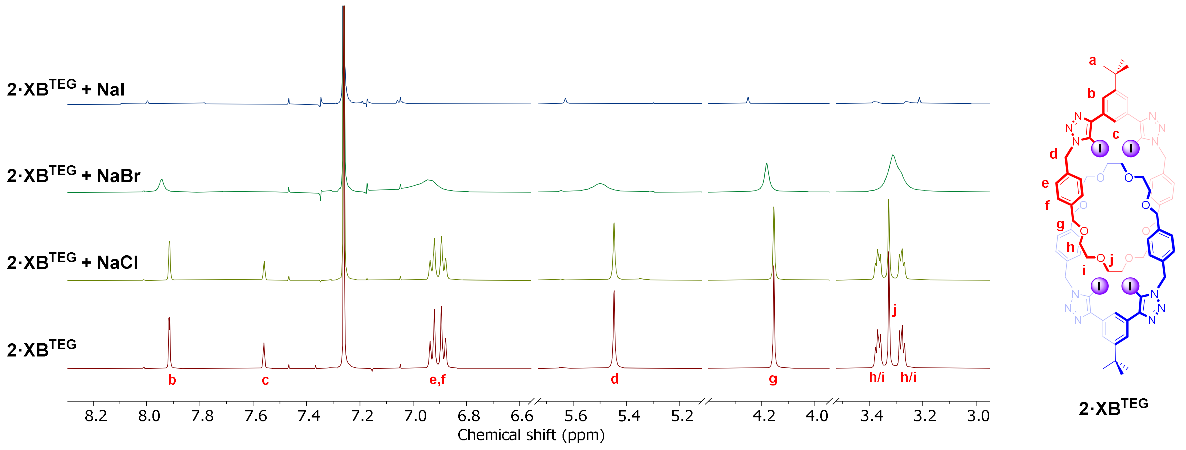

*Figure S72. Pre- and post-extraction ^{1}H NMR spectra of **2·XB^{TEG}** with excess solid NaCl, NaBr and NaI (500 MHz, 298 K, CDCl3).*

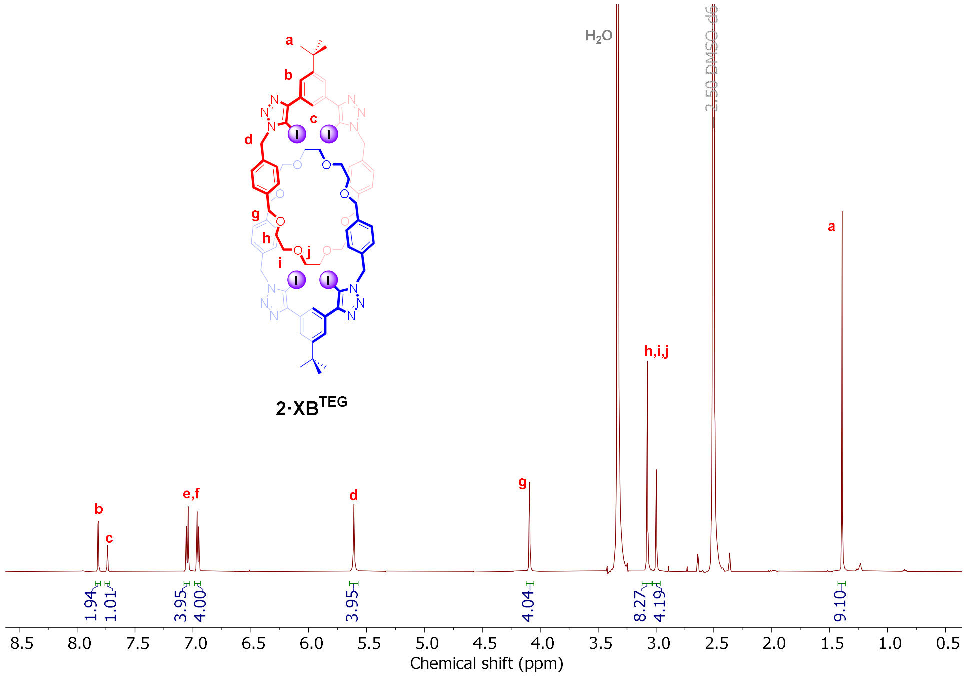

*Figure S73. ^{1}H NMR spectrum of **2·XB^{TEG}** recovered from residue of NaI SLE experiment (500 MHz, 298 K, DMSO-d$_6$).*

Upon treatment of TEG-XB-cat **2·XB^{TEG}** with KX (X = Cl^-, Br^-, I^-), no extraction of KCl was observed. Peak broadening and small chemical shift perturbations were observed with KBr, indicative of partial extraction. A higher degree of KI extraction was seen, evidenced by the significant splitting of the ethylene glycol peaks H_h and H_i, upfield shift of H_j and downfield shifts of all other peaks, including the internal benzene H_c (**Figure S68**).

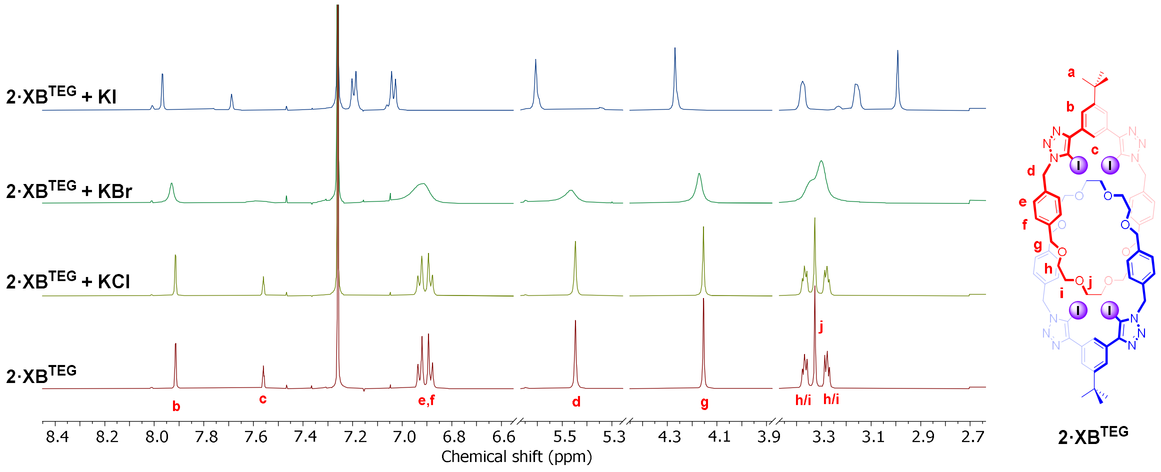

*Figure S74. Pre- and post-extraction ^{1}H NMR spectra of **2·XB^{TEG}** with excess solid KCl, KBr and KI (500 MHz, 298 K, CDCl3).*

As a control, the parent macrocycles **1·XB^{DEG}** and **1·XB^{TEG}** were respectively treated with NaX ($X = Cl^-, Br^-, I^-$) and KX ($X = Cl^-, Br^-, I^-$). No changes in the pre- and post-extraction spectra were observed, indicating no extraction of any of the salts (**Figures S69-70**).

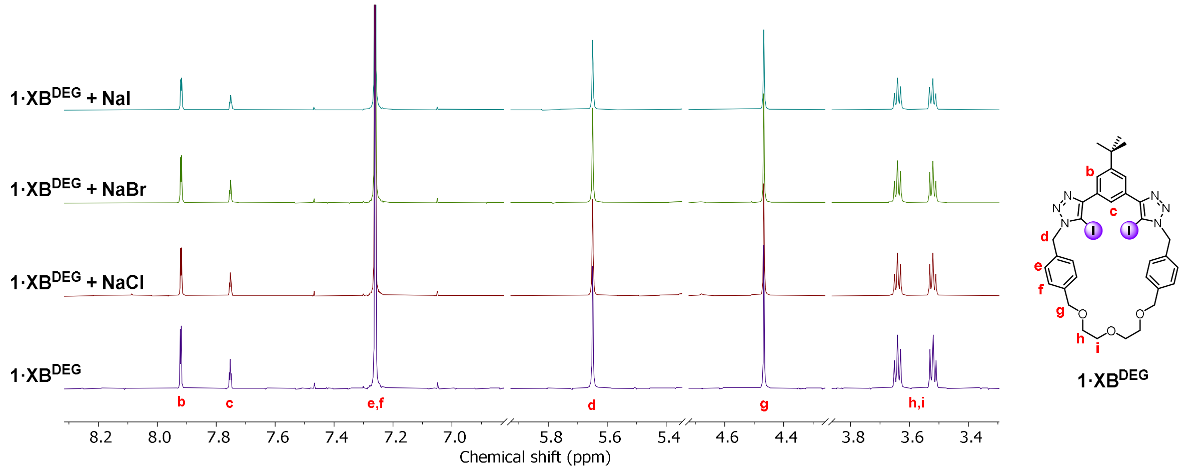

*Figure S75. Pre- and post-extraction ^{1}H NMR spectra of **1·XB^{DEG}** with excess solid NaCl, NaBr and NaI (500 MHz, 298 K, CDCl3).*

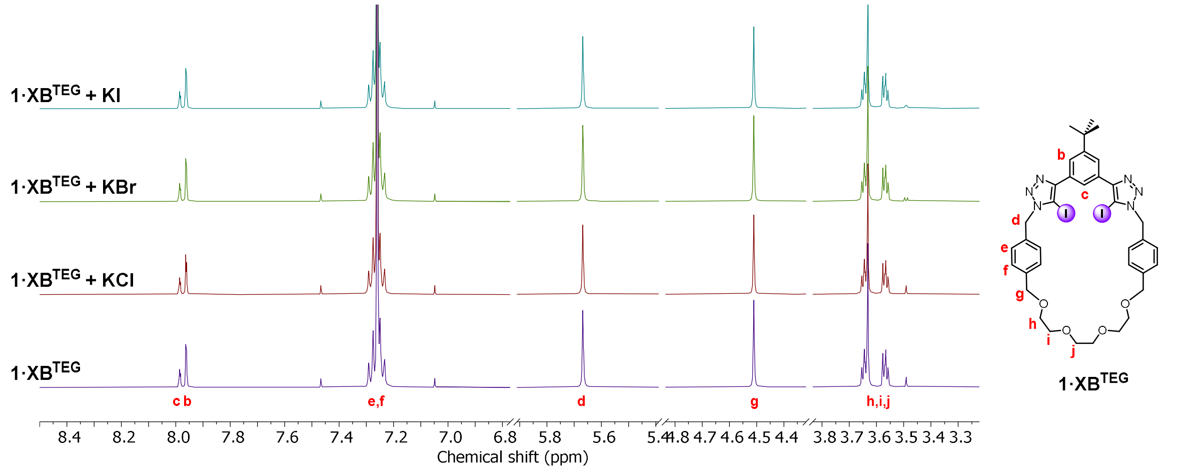

*Figure S76. Pre- and post-extraction ^{1}H NMR spectra of **1·XB^{TEG}** with excess solid KCl, KBr and KI (500 MHz, 298 K, CDCl3).*

Crystal structure determination

General Procedure

Single crystals suitable for X-ray analysis were coated with perfluoropolyether oil, mounted on a 200 µm MiTeGen loop and placed in a cold nitrogen stream (150 K)[6] on an Oxford Diffraction Supernova X-ray diffractometer. Diffraction intensities were measured using monochromated Cu K_α diffraction. Data collection, indexing, initial cell refinements, frame integration, final cell refinements and absorption corrections were performed using CrysAlisPro. Crystal structures were solved by SuperFlip[7] or SHELXS[8] and refined using full matrix least-squares on F^2 with CRYSTALS.[9] Hydrogen atoms were included into the model at geometrically calculated positions and refined using a riding model.[10]

More details are included below and in the accompanying CIF which is part of the supplementary data for this manuscript. These data are also provided free of charge by the joint Cambridge Crystallographic Data Centre and Fachinformationszentrum Karlsruhe Access Structures service www.ccdc.cam.ac.uk/structures with deposition numbers 2211706 (**2·XB^{TEG}**), 2211716 (**2·XB^{DEG}·TBACl**), 2211724 (**2·XB^{DEG}·NaI·H_2O**), 2211718 (**2·XB^{TEG}·NaI**) and 2211717 (**2·HBXB^{DEG}**).

Details for 2·XB^{DEG}·TBACl *Deposition Number 2211716*

The data were extremely noisy and, although they indexed well as tetragonal, internal (intensity) agreement factors were high. Lower symmetry did not improve this significantly, nor did any of the applied twin laws.

The structure solved and refined in the space group P4/n, though it is possible there is a slight distortion away from tetragonal, possibly caused by the behaviour of the disordered solvent sphere at low temperature and partial solvent loss. Careful examination of the raw data supports this thesis, though the data are not of sufficient quality to be able to distinguish between the poor tetragonal result and the similarly poor, but more complex, alternative monoclinic and orthorhombic models.

For this reason, the tetragonal result was chosen as the best fit to the data, but the refinement was poor and it was necessary to apply restraints throughout to maintain sensible geometries and displacement parameters. Predominantly, this was same distance, thermal similarity and vibrational restraints, however, in some cases (notably the polyether chain) it was necessary to apply more specific restraints (as given in the CIF).

The diffuse electron density caused by the disordered solvent was treated by taking the discrete Fourier transform of the void region as contributions to the calculated structure factors using PLATON/SQUEEZE.[11, 12]

Although the quality of the data and therefore the end result is poor, there is no doubt about the connectivity, the interlocked nature of the structure, and the location of the chloride situated in the tetra-iodo pocket.

Details for 2·XB^{DEG}·NaI·H_2O *Deposition Number 2211724*

The data were weak and although they indexed reasonably well, the crystal quality and diffraction were also visibly poor, thought to be caused in part by solvent loss. Despite several attempts to obtain better data or to improve the integration of these data, the results were still poor.

The structure solved well, but on refinement many of the displacement ellipsoids were poorly shaped. Careful examination of the structure suggested that the presence of disorder similar to that seen in Compound 3 (reported herein), but the poor quality and weakness of the data, together with the extent of the disorder and its low occupancy, meant it was not possible to model this effectively. Competitive refinement of the catenane iodine atoms gave a slight reduction in the agreement factors and suggested 6% occupancy for the minor component of the disorder.

As a result, the best model was obtained by the application of thermal similarity and vibrational restraints throughout the structure to maintain sensible displacement parameters together with a small number of same distance restraints where necessary.

In addition to the catenane and the sodium iodide, there was an additional atom coordinated to sodium. Refinement of the occupancy suggested it was an adventitious coordinated water. One of the hydrogen atoms was clearly visible in the difference Fourier map forming a hydrogen bond with a nitrogen atom in a triazole in the catenane. The second hydrogen was not as convincingly visible in the difference Fourier map and neither refined well. So both were positioned at geometric positions and refined using a riding model.

The diffuse electron density caused by disordered solvent was treated by taking the discrete Fourier transform of the void region as contributions to the calculated structure factors using PLATON/SQUEEZE.[11, 12]

Although the data quality and therefore the end result are poor, there is little doubt about the connectivity, the interlocked nature of the structure, the binding of the sodium ion by the polyether, the further coordination by water, and the location of the iodide subject to the four halogen-halogen (iodo-iodo) interactions.

Details for 2·XB^{TEG}·NaI *Deposition Number 2211718*

On inspection of the raw frame data, the crystal was found to twinned by a 180° rotation around the [0 0 1] reciprocal lattice direction (c-axis). The data were processed taking this into account, but the best refinement came from including data from only one component and omitting the small number of poorly fitting low angle reflections.

In addition, the structure exhibited very poorly shaped displacement ellipsoids in one component of the catenane believed to be caused by disorder. A split-site model was used (60:40 ratio), but due to the close proximity of the two components same distance, planarity, thermal similarity and vibrational restraints were required to maintain a sensible geometry and reasonable displacement parameters. This improved the agreement factors, but although it couldn't reasonably be modelled, the shape of the displacement ellipsoids suggested that the disorder stretched beyond region modelled.

In addition to the catenane and the sodium iodide, there was also diffuse electron density thought to be partially occupied, disordered solvent. This was treated by taking the discrete Fourier transform of the void region as contributions to the calculated structure factors using PLATON/SQUEEZE.[11, 12]

The resultant structure is of low quality, but supports the conclusions reported in the manuscript.

Details for 2·HBXB^{DEG} *Deposition Number 2211717*

The data, though a little weak and indicative of a poor mosaicity, indexed, solved and refined well. Enlarged and prolate displacement ellipsoids suggested the presence of disorder in one of the ^t^Bu groups, which was modelled using two components, which required same distance, thermal similarity and vibrational restraints to maintain sensible geometries and displacements.

A small amount of diffuse electron density thought to be partially occupied, disordered solvent was also found. This was treated by taking the discrete Fourier transform of the void region as contributions to the calculated structure factors using PLATON/SQUEEZE.[11, 12]

Details for 2·XB^{TEG} *Deposition Number 2211706*

The data indexed well as monoclinic and initially solved in the space group $P2_1/c$, however, the refinement exhibited signs of extensive disorder. On closer examination, although the structure closely approximates to P21/c, with the systematic absences clearly indicating the presence of a c-glide and the absence of a 2_1 screw axis:

	2_1	a	c	n
**N**	37	885	874	893
**N I>3σ(I)**	23	336	2	336
**<I>**	9.9	33.4	0.2	33.2
**<I/σ(I)>**	3.5	4.8	0.1	4.8

Examination of the structure suggest there are differences in the orientation of the phenyl rings and the conformation of the polyethyl chain between molecules that would otherwise be related by symmetry.

Because of the pseudosymmetry, same distance, thermal similarity and vibrational restraints were necessary to ensure the geometry and displacement parameters remained sensible.

Table S3. Selected crystallographic data for reported structures

**Compound**	**2·XB^{DEG}·TBACl**	**2·XB^{DEG}·NaI·H$_2$O**	**2·XB^{TEG}·NaI**	**2·HBXB^{DEG}**	**2·XB^{TEG}**
**Formula**	$C_{84}H_{108}Cl\ I_4N_{13}O_6$	$C_{68}H_{74}I_5N_{12}NaO_7$	$C_{72}H_{80}I_5N_{12}NaO_8$	$C_{71}H_{80}I_2N_{12}O_7$	$C_{36}H_{40}I_2N_6O_4$
**Formula Weight**	1938.93	1828.92	1899.01	1467.27	874.56
**Temp (K)**	150(2)	150(2)	150(2)	150(2)	150(2)
**Crystal system**	tetragonal	monoclinic	monoclinic	triclinic	monoclinic
**Space Group**	P4/n	$P2_1/n$	$P2_1/c$	P-1	Pc
a **(Å)**	36.7661(10)	18.8455(5)	12.8306(2)	13.7114(3)	14.3321(2)
b **(Å)**	36.7661(10)	18.1755(5)	26.6737(4)	16.6936(6)	34.2258(6)
c **(Å)**	13.8468(8)	24.7474(9)	26.0769(4)	17.3863(4)	14.8140(2)
**α (°)**	90	90	90	68.872(3)	90
**β (°)**	90	95.831(3)	96.7556(15)	84.057(2)	98.7161(14)
**γ (°)**	90	90	90	68.699(3)	90
**Cell Volume (Å^3)**	18717.4(15)	8432.8(4)	8862.6(2)	3456.77(19)	7182.76(19)
Z	8	4	4	2	8
**Reflections collected (all)**	99032	60082	85459	64271	87363
**Reflections (unique)**	18458	16973	18071	14222	17373
R_{int}	0.122	0.101	0.094	0.086	0.096
R_1 **($I > 2\sigma(I)$)**	0.1177	0.1474	0.0917	0.0527	0.0743
**wR_2 (all data)**	0.2679	0.3874	0.2520	0.1447	0.1802

*Crystal structure of **2·XB^{TEG}***

Crystals of **2·XB^{TEG}** suitable for analysis by single crystal X-ray diffraction were obtained by slow evaporation of a solution of **2·XB^{TEG}** in 1:1 $CDCl_3/CD_3CN$. The crystal structure was solved in the monoclinic space group *P*c and was found to consist of [2]catenane units, confirming the interlocked topology of the compound (**Figure S77a**). An examination of the crystal packing revealed the presence of intermolecular halogen bonding interactions between the iodine donor atoms and triazole nitrogen atoms of neighbouring [2]catenanes, giving rise to polymeric XB-stabilised chains running parallel to crystallographic *c* axis (**Figure S77b**).

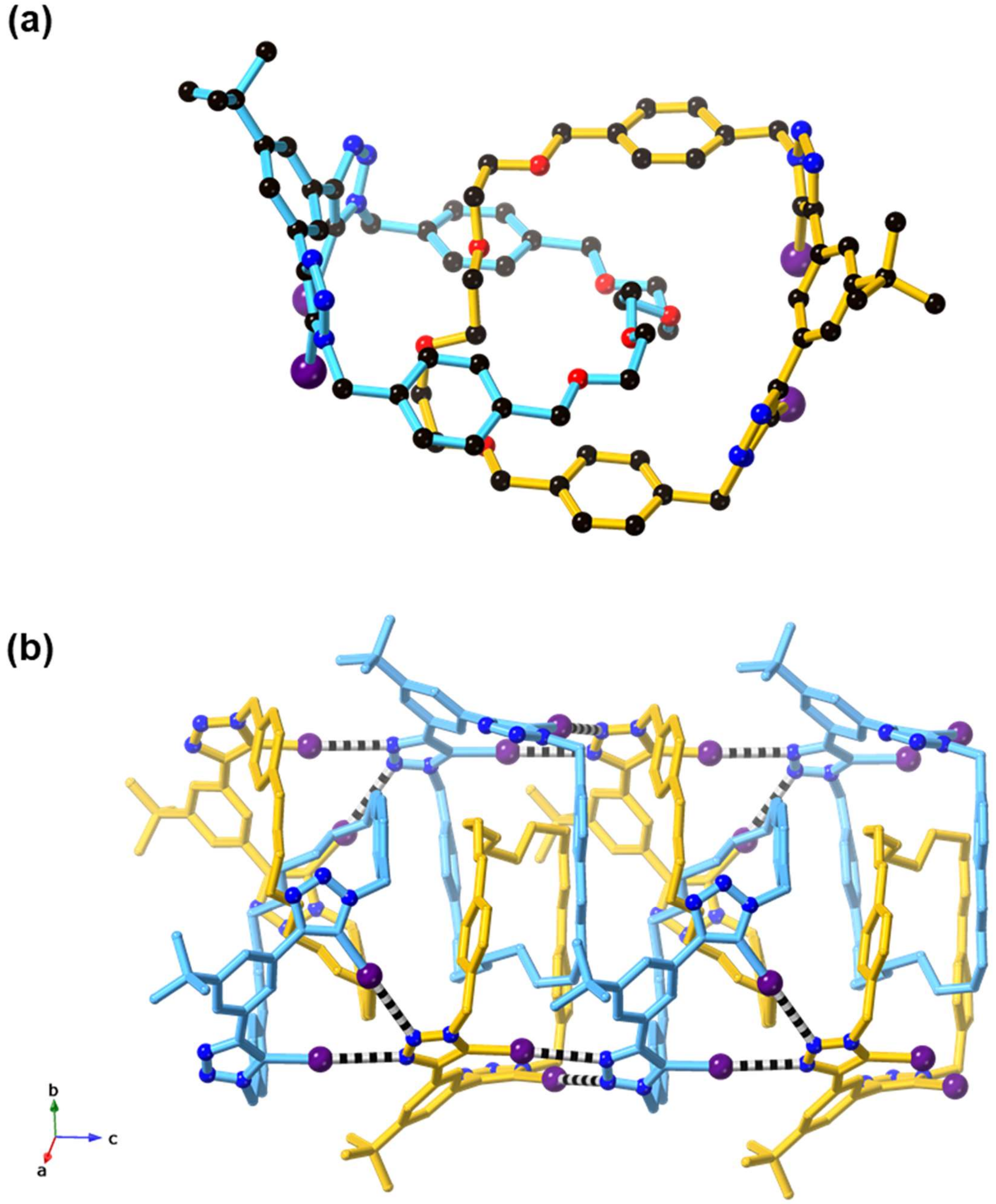

*Figure S77. Crystal structure of **2·XB^{TEG}**, showing: (a) a single [2]catenane unit, with atom colours are as follows: carbon (black), nitrogen (dark blue), oxygen (red), iodine (purple). (b) A stick model of the crystal packing, showing the network of intermolecular halogen bonding interactions between neighbouring [2]catenanes. Iodine and nitrogen atoms are highlighted in using purple and blue spheres respectively. Hydrogen atoms have been omitted for clarity.*

Crystal packing of ***2·XB^{DEG}·NaI·H$_2$O*** *and* ***2·XB^{TEG}·NaI***

The solid-state crystal packing of both structures consists of 1D polymeric chains stabilised by intercomponent XB interactions between the iodotriazole XB donors and iodide guests as shown in **Figures S78** and **S79**.

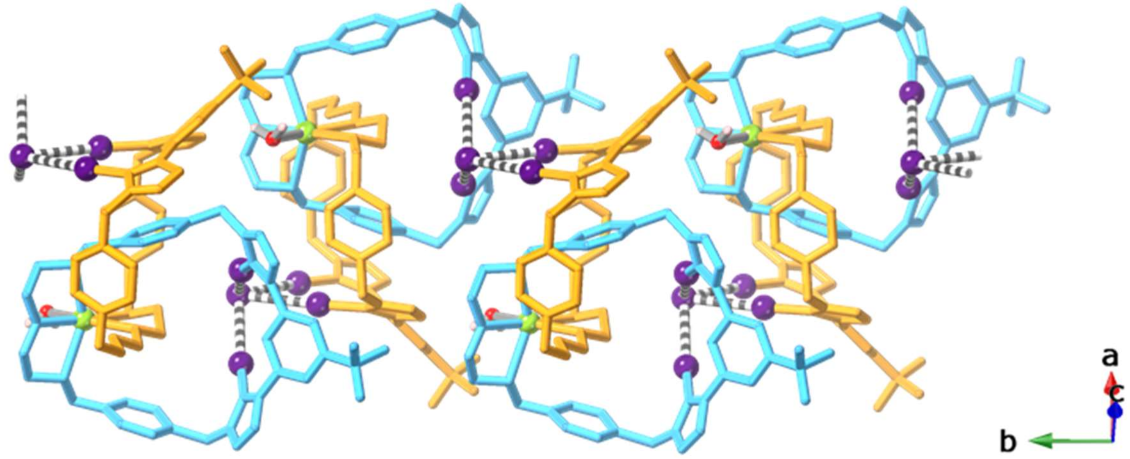

*Figure S78. Crystal packing of **10·NaI·H$_2$O**, showing a stick representation of a halogen-bonded supramolecular chain comprising **10·Na·H$_2$O** units linked by XB interactions to iodide ions (shown in purple).*

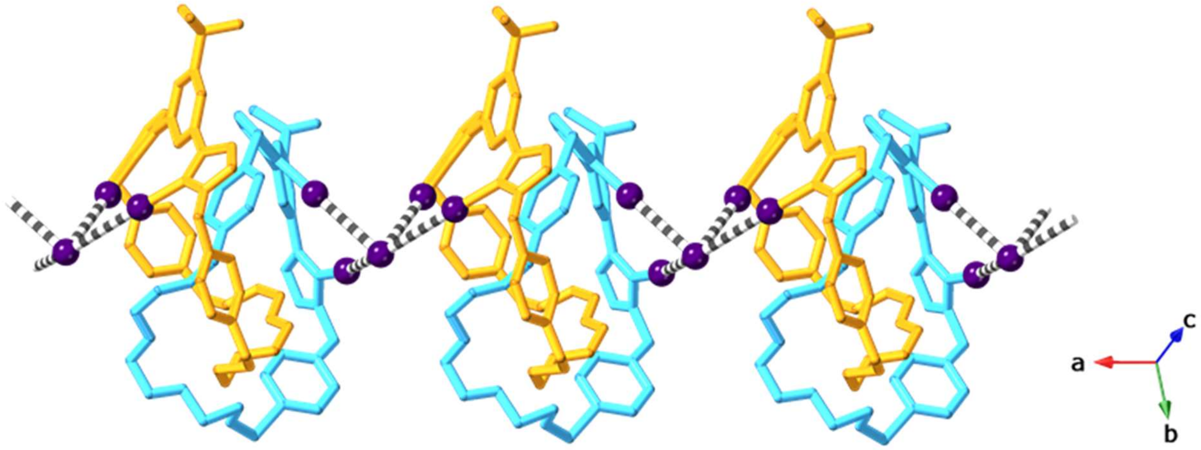

*Figure S79. Crystal packing of **11·NaI**, showing a stick representation of a halogen-bonded supramolecular chain comprising **11·Na** units linked by XB interactions to iodide ions (shown in purple).*

References

1. T. R. Chan, R. Hilgraf, K. B. Sharpless and V. V. Fokin, *Org. Lett.*, 2004, **6**, 2853-2855.
2. Y.-H. Lin, C.-C. Lai and S.-H. Chiu, *Org. Biomol. Chem.*, 2014, **12**, 2907-2917.
3. Y. C. Tse, A. Docker, Z. Zhang and P. D. Beer, *Chem. Commun.*, 2021, **57**, 4950-4953.
4. P. Thordarson, *Chem. Soc. Rev.*, 2011, **40**, 1305-1323.
5. E. N. W. Howe, M. Bhadbhade and P. Thordarson, *J. Am. Chem. Soc.*, 2014, **136**, 7505-7516.
6. J. Cosier and A. M. Glazer, *J. Appl. Crystallogr.*, 1986, **19**, 105-107.
7. L. Palatinus and G. Chapuis, *J. Appl. Crystallogr.*, 2007, **40**.
8. G. Sheldrick, *Acta Cryst. A*, 2015, **71**, 3-8.
9. R. Cooper, P. Betteridge, D. Watkin, K. Prout and J. Carruthers, *J. Appl. Crystallogr.*, 2003, **36**, 1487.
10. R. Cooper, A. Thompson and D. Watkin, *J. Appl. Crystallogr.*, 2010, **43**.
11. A. Spek, *J. Appl. Cryst*, 2003, **36**, 7-13.
12. P. Van Der Sluis and A. L. Spek, *Acta Cryst. A*, 1990, **46**, 194-201.
