# Supplementary material for: Halogen‐Bonding Heteroditopic [2]Catenanes for Recognition of Alkali Metal/Halide Ion Pairs
Source: Angew Chem Int Ed Engl. 2022 Dec 20;62(5):e202214785. doi: 10.1002/anie.202214785 (PMC10108176; doi:10.1002/anie.202214785)

checkCIF/PLATON report

Structure factors have been supplied for datablock(s) 2.HBXB-DEG, 2.XB-DEG.NaI.H2O, 2.XB-DEG.TBACl, 2.XB-TEG, 2.XB-TEG.NaI

THIS REPORT IS FOR GUIDANCE ONLY. IF USED AS PART OF A REVIEW PROCEDURE FOR PUBLICATION, IT SHOULD NOT REPLACE THE EXPERTISE OF AN EXPERIENCED CRYSTALLOGRAPHIC REFEREE.

No syntax errors found. CIF dictionary Interpreting this report

Datablock: 2.XB-DEG.TBACl

Bond precision:	C-C = 0.0151 A	Wavelength=1.54184	
Cell:	a=36.7661(10)	b=36.7661(10)	c=13.8468(8)
	alpha=90	beta=90	gamma=90
Temperature:	150 K		

	Calculated	Reported
Volume	18717.4(15)	18717.4(15)
Space group	P 4/n	P 4/n
Hall group	-P 4a	-P 4a
Moiety formula	2(C34 H36 I2 N6 O3), C16 H36 N, Cl [+ solvent]	2(C34 H36 I2 N6 O3), C16 H36 N, Cl
Sum formula	C84 H108 Cl I4 N13 O6 [+ solvent]	C84 H108 Cl I4 N13 O6
Mr	1938.88	1938.93
Dx,g cm-3	1.376	1.376
Z	8	8
Mu (mm-1)	11.144	11.144
F000	7840.0	7840.0
F000'	7852.59	
h,k,lmax	46,46,17	32,46,17
Nref	20129	19311
Tmin,Tmax	0.146,0.640	0.070,0.640
Tmin'	0.041	

Correction method= # Reported T Limits: Tmin=0.070 Tmax=0.640
AbsCorr = GAUSSIAN

Data completeness= 0.959 Theta(max)= 78.386

```
R(reflections)= 0.1177( 10995)                          wR2(reflections)=
                                                        0.3046( 18458)
S = 0.994                  Npar= 1029
```

The following ALERTS were generated. Each ALERT has the format
test-name_ALERT_alert-type_alert-level.
Click on the hyperlinks for more details of the test.

Alert level A

```
PLAT410_ALERT_2_A Short Intra H...H Contact  H742    ..H761    .      1.76 Ang.
                                                    x,y,z  =      1_555 Check
```

Author Response: H742, H761, H772, H791, and H822 are all part of the polyethyl chain in one component of the DEG-catenane, while H242 and H262 are part of the polyethyl chain in the other. Although the displacement parameters are large suggesting a high degree of thermal and/or positional disorder, the general location of the chain is constrained by the interlocked nature of the structure. While the central location of the displacement parameters leads to these close contacts, allowing for movement within the large displacements accounts for the short distances. Efforts were made to model this as disordered, but it was not possible to resolve separate components and the refinement became unstable as a result. Although this model is less than ideal, there is little doubt about the gross connectivity, the interlocked nature of the structure, and the location of the chloride situated in the tetra-iodo pocket.

```
PLAT410_ALERT_2_A Short Intra H...H Contact  H772    ..H791    .      1.73 Ang.
                                                    x,y,z  =      1_555 Check
```

Author Response: H742, H761, H772, H791, and H822 are all part of the polyethyl chain in one component of the DEG-catenane, while H242 and H262 are part of the polyethyl chain in the other. Although the displacement parameters are large suggesting a high degree of thermal and/or positional disorder, the general location of the chain is constrained by the interlocked nature of the structure. While the central location of the displacement parameters leads to these close contacts, allowing for movement within the large displacements accounts for the short distances. Efforts were made to model this as disordered, but it was not possible to resolve separate components and the refinement became unstable as a result. Although this model is less than ideal, there is little doubt about the gross connectivity, the interlocked nature of the structure, and the location of the chloride situated in the tetra-iodo pocket.

```
PLAT410_ALERT_2_A Short Intra H...H Contact  H791    ..H822    .      1.59 Ang.
                                                    x,y,z  =      1_555 Check
```

Author Response: H742, H761, H772, H791, and H822 are all part of the polyethyl chain in one component of the DEG-catenane, while H242 and H262 are part of the polyethyl chain in the other. Although the displacement parameters are large suggesting a high degree of thermal and/or positional disorder, the general location of the chain is constrained by the interlocked nature of the structure. While the central location of the displacement parameters leads to these close contacts, allowing for movement within the large displacements accounts for the short distances. Efforts were made to model this as disordered, but it was not possible to resolve separate components and the refinement became unstable as a result. Although this model is less than ideal, there is little doubt about the gross connectivity, the interlocked nature of the structure, and the location of the chloride situated in the tetra-iodo pocket.

Alert level C

```
RINTA01_ALERT_3_C  The value of Rint is greater than 0.12
            Rint given   0.122
PLAT020_ALERT_3_C The Value of Rint is Greater Than 0.12 .........      0.122 Report
PLAT027_ALERT_3_C _diffrn_reflns_theta_full value (too) Low ......      66.00 Degree
PLAT029_ALERT_3_C _diffrn_measured_fraction_theta_full value Low .      0.963 Why?
PLAT082_ALERT_2_C High R1 Value ..................................       0.12 Report
PLAT084_ALERT_3_C High wR2 Value (i.e. > 0.25) ...................       0.30 Report
PLAT220_ALERT_2_C NonSolvent   Resd 1  C   Ueq(max)/Ueq(min) Range        3.2 Ratio
PLAT241_ALERT_2_C High   'MainMol' Ueq as Compared to Neighbors of        C83 Check
PLAT260_ALERT_2_C Large Average Ueq of Residue Including       I11      0.113 Check
PLAT260_ALERT_2_C Large Average Ueq of Residue Including       I61      0.111 Check
PLAT260_ALERT_2_C Large Average Ueq of Residue Including      N101      0.107 Check
PLAT334_ALERT_2_C Small <C-C> Benzene Dist.   C18      -C23      .       1.37 Ang.
PLAT334_ALERT_2_C Small <C-C> Benzene Dist.   C33      -C38      .       1.37 Ang.
PLAT334_ALERT_2_C Small <C-C> Benzene Dist.   C68      -C73      .       1.37 Ang.
PLAT334_ALERT_2_C Small <C-C> Benzene Dist.   C83      -C88      .       1.37 Ang.
PLAT342_ALERT_3_C Low Bond Precision on  C-C Bonds ..............     0.0151 Ang.
PLAT410_ALERT_2_C Short Intra H...H Contact  H242     ..H262     .       1.90 Ang.
                                                     x,y,z  =      1_555 Check
```

Author Response: H742, H761, H772, H791, and H822 are all part of the polyethyl chain in one component of the DEG-catenane, while H242 and H262 are part of the polyethyl chain in the other. Although the displacement parameters are large suggesting a high degree of thermal and/or positional disorder, the general location of the chain is constrained by the interlocked nature of the structure. While the central location of the displacement parameters leads to these close contacts, allowing for movement within the large displacements accounts for the short distances. Efforts were made to model this as disordered, but it was not possible to resolve separate components and the refinement became unstable as a result. Although this model is less than ideal, there is little doubt about the gross connectivity, the interlocked nature of the structure, and the location of the chloride situated in the tetra-iodo pocket.

```
PLAT910_ALERT_3_C Missing # of FCF Reflection(s) Below Theta(Min).          5 Note
PLAT911_ALERT_3_C Missing FCF Refl Between Thmin & STh/L=    0.600        655 Report
```

Alert level G

```
PLAT002_ALERT_2_G Number of Distance or Angle Restraints on AtSite         93 Note
PLAT003_ALERT_2_G Number of Uiso or Uij Restrained non-H Atoms ...        113 Report
PLAT093_ALERT_1_G No s.u.'s on H-positions, Refinement Reported as      mixed Check
PLAT175_ALERT_4_G The CIF-Embedded .res File Contains SAME Records          5 Report
PLAT178_ALERT_4_G The CIF-Embedded .res File Contains SIMU Records          2 Report
PLAT301_ALERT_3_G Main Residue  Disorder .............(Resd  1  )          7% Note
PLAT302_ALERT_4_G Anion/Solvent/Minor-Residue Disorder (Resd  2  )         7% Note
PLAT398_ALERT_2_G Deviating  C-O-C   Angle From 120 for O25      .       99.9 Degree
PLAT398_ALERT_2_G Deviating  C-O-C   Angle From 120 for O28      .      100.0 Degree
PLAT398_ALERT_2_G Deviating  C-O-C   Angle From 120 for O31      .       99.9 Degree
PLAT398_ALERT_2_G Deviating  C-O-C   Angle From 120 for O75      .      100.0 Degree
PLAT398_ALERT_2_G Deviating  C-O-C   Angle From 120 for O78      .      100.0 Degree
PLAT398_ALERT_2_G Deviating  C-O-C   Angle From 120 for O81      .       99.9 Degree
PLAT412_ALERT_2_G Short Intra XH3 .. XHn     H31      ..H2091    .       1.98 Ang.
                                                      x,y,z  =      1_555 Check
PLAT412_ALERT_2_G Short Intra XH3 .. XHn     H51      ..H2101    .       2.08 Ang.
                                                      x,y,z  =      1_555 Check
PLAT412_ALERT_2_G Short Intra XH3 .. XHn     H551     ..H2603    .       2.04 Ang.
                                                      x,y,z  =      1_555 Check
PLAT413_ALERT_2_G Short Inter XH3 .. XHn     H1132    ..H2102    .       2.12 Ang.
                                             -1/2+y,1-x,1-z  =      8_466 Check
PLAT432_ALERT_2_G Short Inter X...Y Contact  C58      ..C84      .       3.18 Ang.
                                                    x,y,1+z  =      1_556 Check
PLAT434_ALERT_2_G Short Inter HL..HL Contact I11      ..Cl100    .       3.34 Ang.
                                                      x,y,z  =      1_555 Check
PLAT434_ALERT_2_G Short Inter HL..HL Contact I45      ..Cl100    .       3.16 Ang.
                                                      x,y,z  =      1_555 Check
PLAT434_ALERT_2_G Short Inter HL..HL Contact I61      ..Cl100    .       3.33 Ang.
                                                  y,3/2-x,z  =      3_565 Check
PLAT434_ALERT_2_G Short Inter HL..HL Contact I95      ..Cl100    .       3.18 Ang.
                                                  y,3/2-x,z  =      3_565 Check
PLAT606_ALERT_4_G Solvent Accessible VOID(S) in Structure ........          ! Info
PLAT720_ALERT_4_G Number of Unusual/Non-Standard Labels ..........         55 Note
PLAT721_ALERT_1_G Bond    Calc     0.78000, Rep     0.79500 Dev...       0.02 Ang.
              C209     -H2091           1_555   1_555 ........     #  236 Check
PLAT769_ALERT_4_G CIF Embedded explicitly supplied scattering data     Please Note
PLAT809_ALERT_1_G Can not Parse the SHELXL Weighting Scheme String     Please Check
PLAT860_ALERT_3_G Number of Least-Squares Restraints ............        2058 Note
PLAT869_ALERT_4_G ALERTS Related to the Use of SQUEEZE  Suppressed          ! Info
PLAT912_ALERT_4_G Missing # of FCF Reflections Above STh/L=  0.600       1012 Note
PLAT913_ALERT_3_G Missing # of Very Strong Reflections in FCF ....          1 Note
PLAT929_ALERT_5_G No Weight Pars,Obs and Calc R1,wR2,S not Checked          ! Info
PLAT960_ALERT_3_G Number of Intensities with I  <   - 2*sig(I) ...          2 Check
```

---

3 **ALERT level A** = Most likely a serious problem - resolve or explain
0 **ALERT level B** = A potentially serious problem, consider carefully
19 **ALERT level C** = Check. Ensure it is not caused by an omission or oversight
33 **ALERT level G** = General information/check it is not something unexpected

3 ALERT type 1 CIF construction/syntax error, inconsistent or missing data

```
 31 ALERT type 2 Indicator that the structure model may be wrong or deficient
 12 ALERT type 3 Indicator that the structure quality may be low
  8 ALERT type 4 Improvement, methodology, query or suggestion
  1 ALERT type 5 Informative message, check
```

Datablock: 2.XB-DEG.NaI.H2O

Bond precision: C-C = 0.0279 A Wavelength=1.54184

Cell:	a=18.8455(5)	b=18.1755(5)	c=24.7474(9)
	alpha=90	beta=95.831(3)	gamma=90
Temperature:	150 K		

	Calculated	Reported
Volume	8432.8(4)	8432.8(4)
Space group	P 21/n	P 21/n
Hall group	-P 2yn	-P2yn
Moiety formula	C68 H74 I5 N12 Na O7 [+ solvent]	C68 H74 I5 N12 Na O7
Sum formula	C68 H74 I5 N12 Na O7 [+ solvent]	C68 H74 I5 N12 Na O7
Mr	1828.89	1828.92
Dx,g cm-3	1.441	1.440
Z	4	4
Mu (mm-1)	14.942	14.942
F000	3592.0	3592.0
F000'	3596.08	
h,k,lmax	23,22,31	23,22,30
Nref	17849	17339
Tmin,Tmax	0.392,0.550	0.020,0.550
Tmin'	0.000	

Correction method= # Reported T Limits: Tmin=0.020 Tmax=0.550
AbsCorr = GAUSSIAN

Data completeness= 0.971 Theta(max)= 77.152

R(reflections)= 0.1474(11825) wR2(reflections)= 0.4105(16973)

S = 0.967 Npar= 838

```
The following ALERTS were generated. Each ALERT has the format
       test-name_ALERT_alert-type_alert-level.
Click on the hyperlinks for more details of the test.
```

Alert level B

```
PLAT084_ALERT_3_B High wR2 Value (i.e. > 0.25) ..................       0.41 Report
```

Author Response: The data quality is poor and careful examination of the refeinment suggests the presence of disorder similar to that seen in Compound 3 (repoorted herein), that it was not possible to resolve. Although the data and therefore the result are less than ideal, there is little doubt about the gross connectivity, the interlocked nature of the structure, the binding of the sodium ion by the polyether, the further coordination by water, and the location of the iodide subject to the four halogen-halogen (iodo-iodo) interactions.

```
PLAT097_ALERT_2_B Large Reported Max.  (Positive) Residual Density       5.79 eA-3
```

Author Response: The large amount of residual electron density is believed to result from the unresolved disorder described in more detail elsewhere. Competitive refinement of just the two largest residual peaks as iodine atoms lead to a slight reduction in the agreement factors and suggested 6% occupancy for the minor component of the disorder.

```
PLAT342_ALERT_3_B Low Bond Precision on  C-C Bonds ..............    0.02789 Ang.
```

Author Response: The poor quality and weakness of the data, together with the unresolved disorder result in a poor quality result, with a low bond precision. Despite the poor qulity of the data, there is little doubt about the connectivity, the interlocked nature of the structure, the binding of the sodium ion by the polyethers, the coordination of the sodium by the water, and the location of the iodide subject to the four halogen-halogen (iodo-iodo) interactions.

```
PLAT420_ALERT_2_B D-H Bond Without Acceptor  O50      --H502     .     Please Check
```

Author Response: H502 is part of a water molecule coordinated to the encapsulated sodium ion. The other hydrogen atom was visible in the difference Fourier map forming a strong hydrogen bond. Although H502 was not as visible in the difference Fourier map there are few other possibilities.

Alert level C

```
DIFMX02_ALERT_1_C  The maximum difference density is > 0.1*ZMAX*0.75
            The relevant atom site should be identified.
PLAT029_ALERT_3_C _diffrn_measured_fraction_theta_full value Low .      0.967 Why?
PLAT082_ALERT_2_C High R1 Value ................................       0.15 Report
```

```
PLAT334_ALERT_2_C Small <C-C> Benzene Dist.   C18      -C23      .       1.37 Ang.
PLAT334_ALERT_2_C Small <C-C> Benzene Dist.   C68      -C73      .       1.36 Ang.
PLAT334_ALERT_2_C Small <C-C> Benzene Dist.   C83      -C88      .       1.37 Ang.
PLAT363_ALERT_2_C Long   C(sp3)-C(sp2) Bond  C67      - C68      .       1.63 Ang.
PLAT911_ALERT_3_C Missing FCF Refl Between Thmin & STh/L=    0.600        312 Report
```

Alert level G

```
PLAT002_ALERT_2_G Number of Distance or Angle Restraints on AtSite         45 Note
PLAT003_ALERT_2_G Number of Uiso or Uij Restrained non-H Atoms ...         92 Report
PLAT004_ALERT_5_G Polymeric Structure Found with Maximum Dimension          1 Info
PLAT007_ALERT_5_G Number of Unrefined Donor-H Atoms .............           2 Report
PLAT072_ALERT_2_G SHELXL First  Parameter in WGHT  Unusually Large       0.17 Report
PLAT175_ALERT_4_G The CIF-Embedded .res File Contains SAME Records          3 Report
PLAT335_ALERT_2_G Check Large C6 Ring C-C Range C68      -C73            0.24 Ang.
PLAT606_ALERT_4_G Solvent Accessible VOID(S) in Structure ........          ! Info
PLAT769_ALERT_4_G CIF Embedded explicitly supplied scattering data     Please Note
PLAT809_ALERT_1_G Can not Parse the SHELXL Weighting Scheme String     Please Check
PLAT860_ALERT_3_G Number of Least-Squares Restraints ............        1149 Note
PLAT869_ALERT_4_G ALERTS Related to the Use of SQUEEZE  Suppressed          ! Info
PLAT910_ALERT_3_G Missing # of FCF Reflection(s) Below Theta(Min).          2 Note
PLAT912_ALERT_4_G Missing # of FCF Reflections Above STh/L=  0.600        546 Note
PLAT913_ALERT_3_G Missing # of Very Strong Reflections in FCF ....          1 Note
PLAT929_ALERT_5_G No Weight Pars,Obs and Calc R1,wR2,S not Checked          ! Info
PLAT960_ALERT_3_G Number of Intensities with I  <   - 2*sig(I) ...         24 Check
```

---

0 **ALERT level A** = Most likely a serious problem - resolve or explain
4 **ALERT level B** = A potentially serious problem, consider carefully
8 **ALERT level C** = Check. Ensure it is not caused by an omission or oversight
17 **ALERT level G** = General information/check it is not something unexpected

2 ALERT type 1 CIF construction/syntax error, inconsistent or missing data
11 ALERT type 2 Indicator that the structure model may be wrong or deficient
8 ALERT type 3 Indicator that the structure quality may be low
5 ALERT type 4 Improvement, methodology, query or suggestion
3 ALERT type 5 Informative message, check

Datablock: 2.XB-TEG.NaI

Bond precision:	C-C = 0.0157 A		Wavelength=1.54184
Cell:	a=12.8306(2)	b=26.6737(4)	c=26.0769(4)
	alpha=90	beta=96.7556(15)	gamma=90
Temperature:	150 K		

	Calculated	Reported
Volume	8862.6(2)	8862.6(2)
Space group	P 21/c	P 1 21/c 1
Hall group	-P 2ybc	-P 2ybc
Moiety formula	C72 H80 I5 N12 Na O8 [+ solvent]	C72 H80 I5 N12 Na O8
Sum formula	C72 H80 I5 N12 Na O8 [+ solvent]	C72 H80 I5 N12 Na O8
Mr	1898.98	1899.01
Dx,g cm-3	1.423	1.423
Z	4	4
Mu (mm-1)	14.247	14.247
F000	3744.0	3744.0
F000'	3748.45	
h,k,lmax	16,33,32	16,33,32
Nref	18646	18077
Tmin,Tmax	0.226,0.425	0.010,0.430
Tmin'	0.000	

Correction method= # Reported T Limits: Tmin=0.010 Tmax=0.430
AbsCorr = GAUSSIAN

Data completeness= 0.969 Theta(max)= 76.630

R(reflections)= 0.0917(14474) wR2(reflections)= 0.2671(18071)

S = 1.011 Npar= 1172

The following ALERTS were generated. Each ALERT has the format
test-name_ALERT_alert-type_alert-level.
Click on the hyperlinks for more details of the test.

Alert level C

```
PLAT084_ALERT_3_C High wR2 Value (i.e. > 0.25) ...................       0.27 Report
PLAT241_ALERT_2_C High   'MainMol' Ueq as Compared to Neighbors of        C20 Check
PLAT242_ALERT_2_C Low    'MainMol' Ueq as Compared to Neighbors of        Na1 Check
PLAT242_ALERT_2_C Low    'MainMol' Ueq as Compared to Neighbors of        C18 Check
PLAT334_ALERT_2_C Small <C-C> Benzene Dist.   C68      -C73      .       1.36 Ang.
PLAT334_ALERT_2_C Small <C-C> Benzene Dist.   C168     -C173     .       1.37 Ang.
PLAT342_ALERT_3_C Low Bond Precision on  C-C Bonds ...............    0.01574 Ang.
PLAT413_ALERT_2_C Short Inter XH3 .. XHn     H92      ..H241     .       2.12 Ang.
                                                 -x,1-y,2-z  =      2_567 Check
PLAT906_ALERT_3_C Large K Value in the Analysis of Variance ......      5.717 Check
PLAT906_ALERT_3_C Large K Value in the Analysis of Variance ......      2.099 Check
PLAT911_ALERT_3_C Missing FCF Refl Between Thmin & STh/L=    0.600        304 Report
```

Alert level G

```
PLAT002_ALERT_2_G Number of Distance or Angle Restraints on AtSite        102 Note
PLAT003_ALERT_2_G Number of Uiso or Uij Restrained non-H Atoms ...          2 Report
PLAT004_ALERT_5_G Polymeric Structure Found with Maximum Dimension          1 Info
PLAT063_ALERT_4_G Crystal Size Possibly too Large for Beam Size ..       0.63 mm
PLAT072_ALERT_2_G SHELXL First  Parameter in WGHT  Unusually Large       0.14 Report
PLAT083_ALERT_2_G SHELXL Second Parameter in WGHT  Unusually Large      63.04 Why ?
PLAT175_ALERT_4_G The CIF-Embedded .res File Contains SAME Records          5 Report
PLAT177_ALERT_4_G The CIF-Embedded .res File Contains DELU Records          6 Report
PLAT178_ALERT_4_G The CIF-Embedded .res File Contains SIMU Records          8 Report
PLAT301_ALERT_3_G Main Residue  Disorder ..............(Resd  1  )        33% Note
PLAT335_ALERT_2_G Check Large C6 Ring C-C Range C68      -C73            0.21 Ang.
PLAT335_ALERT_2_G Check Large C6 Ring C-C Range C168     -C173           0.21 Ang.
PLAT410_ALERT_2_G Short Intra H...H Contact  H792     ..H1771     .      2.01 Ang.
                                                    x,y,z  =      1_555 Check
PLAT413_ALERT_2_G Short Inter XH3 .. XHn     H102     ..H742      .      2.02 Ang.
                                           -x,1/2+y,3/2-z  =      3_556 Check
PLAT606_ALERT_4_G Solvent Accessible VOID(S) in Structure ........          ! Info
PLAT720_ALERT_4_G Number of Unusual/Non-Standard Labels ..........         24 Note
PLAT769_ALERT_4_G CIF Embedded explicitly supplied scattering data     Please Note
PLAT811_ALERT_5_G No ADDSYM Analysis: Too Many Excluded Atoms ....          ! Info
PLAT860_ALERT_3_G Number of Least-Squares Restraints .............       2063 Note
PLAT869_ALERT_4_G ALERTS Related to the Use of SQUEEZE  Suppressed          ! Info
PLAT910_ALERT_3_G Missing # of FCF Reflection(s) Below Theta(Min).          3 Note
PLAT912_ALERT_4_G Missing # of FCF Reflections Above STh/L=  0.600        268 Note
PLAT913_ALERT_3_G Missing # of Very Strong Reflections in FCF ....          1 Note
PLAT960_ALERT_3_G Number of Intensities with I  <   - 2*sig(I) ...         21 Check
PLAT992_ALERT_5_G Repd & Actual _reflns_number_gt Values Differ by          2 Check
```

```
   0 ALERT level A = Most likely a serious problem - resolve or explain
   0 ALERT level B = A potentially serious problem, consider carefully
  11 ALERT level C = Check. Ensure it is not caused by an omission or oversight
  25 ALERT level G = General information/check it is not something unexpected

   0 ALERT type 1 CIF construction/syntax error, inconsistent or missing data
  14 ALERT type 2 Indicator that the structure model may be wrong or deficient
  10 ALERT type 3 Indicator that the structure quality may be low
   9 ALERT type 4 Improvement, methodology, query or suggestion
   3 ALERT type 5 Informative message, check
```

Datablock: 2.HBXB-DEG

Bond precision:	C-C = 0.0076 A		Wavelength=1.54184
Cell:	a=13.7114(3)	b=16.6936(6)	c=17.3863(4)
	alpha=68.872(3)	beta=84.057(2)	gamma=68.699(3)
Temperature:	150 K		

	Calculated	Reported
Volume	3456.8(2)	3456.77(19)
Space group	P -1	P -1
Hall group	-P 1	-P 1
Moiety formula	C34 H38 N6 O3, C34 H36 I2 N6 O3, C3 H6 O [+ solvent]	C71 H80 I2 N12 O7
Sum formula	C71 H80 I2 N12 O7 [+ solvent]	C71 H80 I2 N12 O7
Mr	1467.27	1467.30
Dx,g cm-3	1.410	1.410
Z	2	2
Mu (mm-1)	7.639	7.639
F000	1504.0	1504.0
F000'	1506.73	
h,k,lmax	17,21,21	17,20,21
Nref	14521	14273
Tmin,Tmax	0.728,0.795	0.200,0.830
Tmin'	0.131	

Correction method= # Reported T Limits: Tmin=0.200 Tmax=0.830
AbsCorr = GAUSSIAN

Data completeness= 0.983 Theta(max)= 76.541

R(reflections)= 0.0527(11002) wR2(reflections)= 0.1610(14222)

S = 0.979 Npar= 857

The following ALERTS were generated. Each ALERT has the format
test-name_ALERT_alert-type_alert-level.
Click on the hyperlinks for more details of the test.

Alert level C

```
PLAT244_ALERT_4_C Low    'Solvent' Ueq as Compared to Neighbors of       C102 Check
PLAT911_ALERT_3_C Missing FCF Refl Between Thmin & STh/L=    0.600         46 Report
```

Alert level G

```
PLAT002_ALERT_2_G Number of Distance or Angle Restraints on AtSite          8 Note
PLAT003_ALERT_2_G Number of Uiso or Uij Restrained non-H Atoms ...          2 Report
PLAT042_ALERT_1_G Calc. and Reported MoietyFormula Strings  Differ     Please Check
PLAT175_ALERT_4_G The CIF-Embedded .res File Contains SAME Records          1 Report
PLAT177_ALERT_4_G The CIF-Embedded .res File Contains DELU Records          1 Report
PLAT178_ALERT_4_G The CIF-Embedded .res File Contains SIMU Records          1 Report
PLAT301_ALERT_3_G Main Residue  Disorder .............(Resd  1  )         7% Note
PLAT412_ALERT_2_G Short Intra XH3 .. XHn     H531      ..H1582    .       1.98 Ang.
                                                         x,y,z  =      1_555 Check
```

```
PLAT431_ALERT_2_G Short Inter HL..A Contact  I11      ..N63      .       3.14 Ang.
                                               1-x,1-y,1-z  =      2_666 Check
PLAT431_ALERT_2_G Short Inter HL..A Contact  I51      ..N63      .       3.10 Ang.
                                               1-x,1-y,1-z  =      2_666 Check
PLAT605_ALERT_4_G Largest Solvent Accessible VOID in the Structure         56 A**3
PLAT720_ALERT_4_G Number of Unusual/Non-Standard Labels ..........         15 Note
PLAT769_ALERT_4_G CIF Embedded explicitly supplied scattering data     Please Note
PLAT860_ALERT_3_G Number of Least-Squares Restraints .............        235 Note
PLAT869_ALERT_4_G ALERTS Related to the Use of SQUEEZE  Suppressed          ! Info
PLAT910_ALERT_3_G Missing # of FCF Reflection(s) Below Theta(Min).          1 Note
PLAT912_ALERT_4_G Missing # of FCF Reflections Above STh/L=  0.600        247 Note
PLAT960_ALERT_3_G Number of Intensities with I  <   - 2*sig(I) ...          3 Check
```

```
   0 ALERT level A = Most likely a serious problem - resolve or explain
   0 ALERT level B = A potentially serious problem, consider carefully
   2 ALERT level C = Check. Ensure it is not caused by an omission or oversight
  18 ALERT level G = General information/check it is not something unexpected

   1 ALERT type 1 CIF construction/syntax error, inconsistent or missing data
   5 ALERT type 2 Indicator that the structure model may be wrong or deficient
   5 ALERT type 3 Indicator that the structure quality may be low
   9 ALERT type 4 Improvement, methodology, query or suggestion
   0 ALERT type 5 Informative message, check
```

Datablock: 2.XB-TEG

```
Bond precision:   C-C = 0.0204 A          Wavelength=1.54184

Cell:             a=14.3321(2)     b=34.2258(6)        c=14.8140(2)
                  alpha=90         beta=98.7161(14)    gamma=90
Temperature:      150 K
```

	Calculated	Reported
Volume	7182.76(19)	7182.76(19)
Space group	P c	P 1 c 1
Hall group	P -2yc	P -2yc
Moiety formula	C36 H40 I2 N6 O4	C36 H40 I2 N6 O4
Sum formula	C36 H40 I2 N6 O4	C36 H40 I2 N6 O4
Mr	874.54	874.56
Dx,g cm-3	1.617	1.617
Z	8	8
Mu (mm-1)	14.136	14.136
F000	3488.0	3488.0
F000'	3492.41	
h,k,lmax	18,43,18	17,42,18
Nref	30157[15100]	17374
Tmin,Tmax	0.533,0.568	0.030,0.570
Tmin'	0.001	

Correction method= # Reported T Limits: Tmin=0.030 Tmax=0.570
AbsCorr = GAUSSIAN

Data completeness= 1.15/0.58 Theta(max)= 76.532

R(reflections)= 0.0612(14389) wR2(reflections)= 0.1803(17374)

S = 1.002 Npar= 1758

The following ALERTS were generated. Each ALERT has the format
test-name_ALERT_alert-type_alert-level.
Click on the hyperlinks for more details of the test.

Alert level B

PLAT220_ALERT_2_B NonSolvent Resd 4 C Ueq(max)/Ueq(min) Range 6.3 Ratio

Author Response: The pseudosymmetry makes the refinement unstable, and inspite of thermal similarity and vibrational restraints, residue 4 (ansd to a lesser extent residue 1) still shows some anisotropy in the displacements.

PLAT342_ALERT_3_B Low Bond Precision on C-C Bonds .... 0.02036 Ang.

Author Response: The pseudosymmetry leads to a high degree of corelation reducing the precision of the end result. Despite this, the resultant structure supports the conclusions reported in the manuscript.

Alert level C

```
PLAT213_ALERT_2_C Atom C110           has ADP max/min Ratio .....        3.1 oblate
PLAT213_ALERT_2_C Atom C59            has ADP max/min Ratio .....        3.5 prolat
PLAT220_ALERT_2_C NonSolvent   Resd 1  C   Ueq(max)/Ueq(min) Range        5.1 Ratio
```

Author Response: The pseudosymmetry makes the refinement unstable, and inspite of thermal similarity and vibrational restraints, residue 4 (ansd to a lesser extent residue 1) still shows some anisotropy in the displacements.

```
PLAT220_ALERT_2_C NonSolvent   Resd 2  C   Ueq(max)/Ueq(min) Range        3.2 Ratio
```

Author Response: The pseudosymmetry makes the refinement unstable, and inspite of thermal similarity and vibrational restraints, residue 4 (ansd to a lesser extent residue 1) still shows some anisotropy in the displacements.

```
PLAT220_ALERT_2_C NonSolvent   Resd 3  C   Ueq(max)/Ueq(min) Range        3.4 Ratio
```

Author Response: The pseudosymmetry makes the refinement unstable, and inspite of thermal similarity and vibrational restraints, residue 4 (ansd to a lesser extent residue 1) still shows some anisotropy in the displacements.

```
PLAT222_ALERT_3_C NonSolvent Resd 1  H   Uiso(max)/Uiso(min) Range        5.1 Ratio
PLAT222_ALERT_3_C NonSolvent Resd 4  H   Uiso(max)/Uiso(min) Range        5.3 Ratio
PLAT234_ALERT_4_C Large Hirshfeld Difference C119     --C120     .       0.19 Ang.
PLAT234_ALERT_4_C Large Hirshfeld Difference C121     --C122     .       0.21 Ang.
PLAT234_ALERT_4_C Large Hirshfeld Difference C137     --C138     .       0.19 Ang.
PLAT234_ALERT_4_C Large Hirshfeld Difference C36      --C41      .       0.18 Ang.
PLAT234_ALERT_4_C Large Hirshfeld Difference C187     --C188     .       0.17 Ang.
PLAT241_ALERT_2_C High   'MainMol' Ueq as Compared to Neighbors of       O128 Check
PLAT241_ALERT_2_C High   'MainMol' Ueq as Compared to Neighbors of       C190 Check
PLAT242_ALERT_2_C Low    'MainMol' Ueq as Compared to Neighbors of         C7 Check
PLAT242_ALERT_2_C Low    'MainMol' Ueq as Compared to Neighbors of        C57 Check
PLAT334_ALERT_2_C Small <C-C> Benzene Dist.   C68       -C73       .       1.36 Ang.
PLAT411_ALERT_2_C Short Inter H...H Contact  H1272    ..H1821    .       2.14 Ang.
                                                      x,y,z  =      1_555 Check
PLAT412_ALERT_2_C Short Intra XH3 .. XHn     H31      ..H82      .       1.80 Ang.
                                                      x,y,z  =      1_555 Check
PLAT413_ALERT_2_C Short Inter XH3 .. XHn     H1422    ..H1602    .       2.14 Ang.
                                                      x,y,z  =      1_555 Check
```

Alert level G

```
PLAT002_ALERT_2_G Number of Distance or Angle Restraints on AtSite        120 Note
PLAT003_ALERT_2_G Number of Uiso or Uij Restrained non-H Atoms ...          2 Report
PLAT072_ALERT_2_G SHELXL First  Parameter in WGHT  Unusually Large       0.11 Report
PLAT083_ALERT_2_G SHELXL Second Parameter in WGHT  Unusually Large      17.01 Why ?
PLAT175_ALERT_4_G The CIF-Embedded .res File Contains SAME Records          4 Report
PLAT177_ALERT_4_G The CIF-Embedded .res File Contains DELU Records         21 Report
PLAT178_ALERT_4_G The CIF-Embedded .res File Contains SIMU Records          5 Report
```

```
PLAT301_ALERT_3_G Main Residue  Disorder .............(Resd  1  )         6% Note
PLAT412_ALERT_2_G Short Intra XH3 .. XHn     H1031    ..H2091    .       2.11 Ang.
                                                      x,y,z  =      1_555 Check
PLAT412_ALERT_2_G Short Intra XH3 .. XHn     H1051    ..H1102    .       1.82 Ang.
                                                      x,y,z  =      1_555 Check
PLAT431_ALERT_2_G Short Inter HL..A Contact  I11      ..N64      .       3.12 Ang.
                                                      x,y,z  =      1_555 Check
PLAT431_ALERT_2_G Short Inter HL..A Contact  I48      ..N65      .       3.24 Ang.
                                                      x,y,z  =      1_555 Check
PLAT431_ALERT_2_G Short Inter HL..A Contact  I61      ..N14      .       3.17 Ang.
                                                   x,y,-1+z  =      1_554 Check
PLAT431_ALERT_2_G Short Inter HL..A Contact  I98      ..N15      .       3.02 Ang.
                                                   x,y,-1+z  =      1_554 Check
PLAT431_ALERT_2_G Short Inter HL..A Contact  I111     ..N194     .       3.24 Ang.
                                                      x,y,z  =      1_555 Check
PLAT431_ALERT_2_G Short Inter HL..A Contact  I148     ..N195     .       3.13 Ang.
                                                      x,y,z  =      1_555 Check
PLAT431_ALERT_2_G Short Inter HL..A Contact  I161     ..N144     .       2.94 Ang.
                                                    x,y,1+z  =      1_556 Check
PLAT431_ALERT_2_G Short Inter HL..A Contact  I198     ..N145     .       3.15 Ang.
                                                    x,y,1+z  =      1_556 Check
PLAT720_ALERT_4_G Number of Unusual/Non-Standard Labels ..........         89 Note
PLAT769_ALERT_4_G CIF Embedded explicitly supplied scattering data     Please Note
PLAT790_ALERT_4_G Centre of Gravity not Within Unit Cell: Resd.  #          4 Note
              C36 H40 I2 N6 O4
PLAT860_ALERT_3_G Number of Least-Squares Restraints .............       2285 Note
PLAT910_ALERT_3_G Missing # of FCF Reflection(s) Below Theta(Min).          2 Note
PLAT912_ALERT_4_G Missing # of FCF Reflections Above STh/L=  0.600        163 Note
PLAT915_ALERT_3_G No Flack x Check Done: Low Friedel Pair Coverage         16 %
PLAT960_ALERT_3_G Number of Intensities with I  <   - 2*sig(I) ...         13 Check
```

---

  0 **ALERT level A** = Most likely a serious problem - resolve or explain
  2 **ALERT level B** = A potentially serious problem, consider carefully
 20 **ALERT level C** = Check. Ensure it is not caused by an omission or oversight
 26 **ALERT level G** = General information/check it is not something unexpected

  0 ALERT type 1 CIF construction/syntax error, inconsistent or missing data
 28 ALERT type 2 Indicator that the structure model may be wrong or deficient
  8 ALERT type 3 Indicator that the structure quality may be low
 12 ALERT type 4 Improvement, methodology, query or suggestion
  0 ALERT type 5 Informative message, check

Publication of your CIF

You should attempt to resolve as many as possible of the alerts in all categories. Often the minor alerts point to easily fixed oversights, errors and omissions in your CIF or refinement strategy, so attention to these fine details can be worthwhile. In order to resolve some of the more serious problems it may be necessary to carry out additional measurements or structure refinements. However, the nature of your study may justify the reported deviations from journal submission requirements and the more serious of these should be commented upon in the discussion or experimental section of a paper or in the "special_details" fields of the CIF. *checkCIF* was carefully designed to identify outliers and unusual parameters, but every test has its limitations and alerts that are not important in a particular case may appear. Conversely, the absence of alerts does not guarantee there are no aspects of the results needing attention. It is up to the individual to critically assess their own results and, if necessary, seek expert advice.

If you wish to submit your CIF for publication in Acta Crystallographica Section C or E, you should upload your CIF via the web. If you wish to submit your CIF for publication in IUCrData, you should upload your CIF via the web. If your CIF is to form part of a submission to another IUCr journal, you will be asked, either during electronic submission or by the Co-editor handling your paper, to upload your CIF via our web site.

PLATON version of 12/09/2022; check.def file version of 09/08/2022

Datablock 2.XB-DEG.TBACl - ellipsoid plot

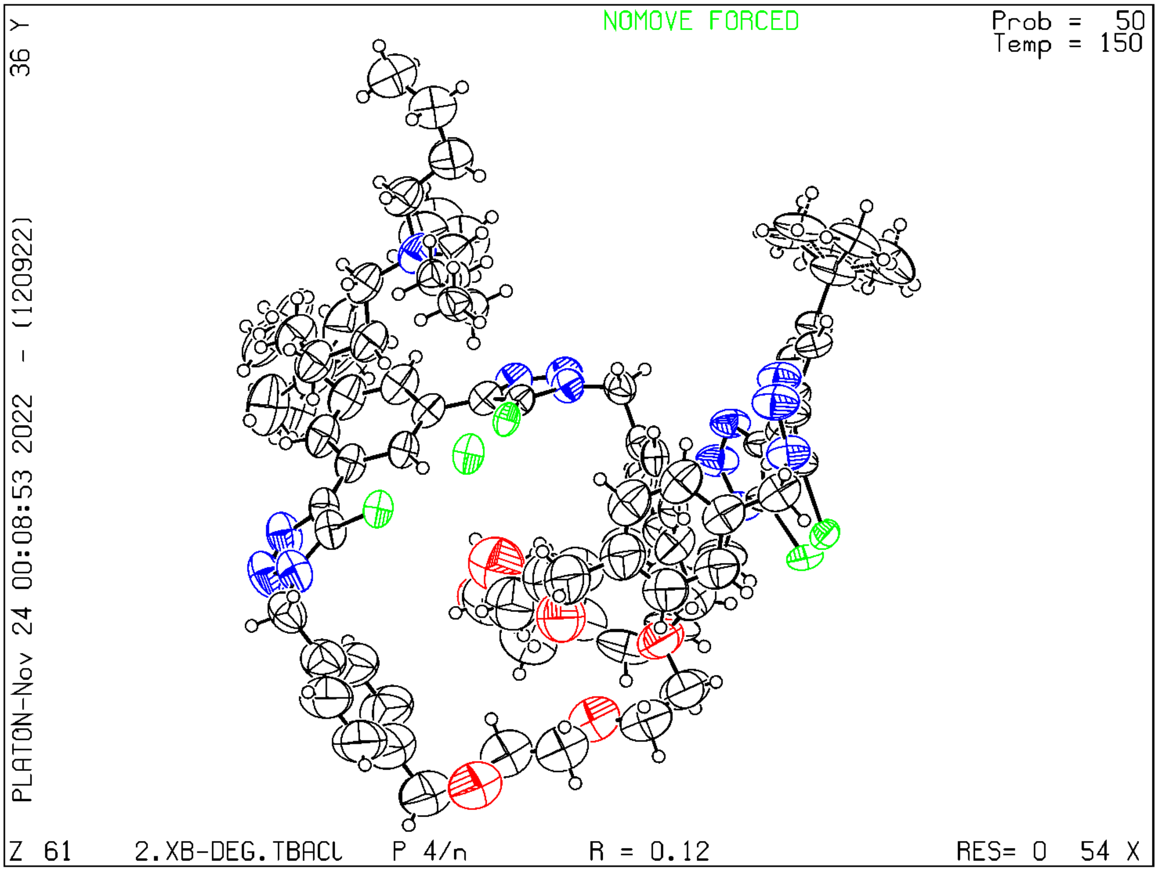

Datablock 2.XB-DEG.NaI.H2O - ellipsoid plot

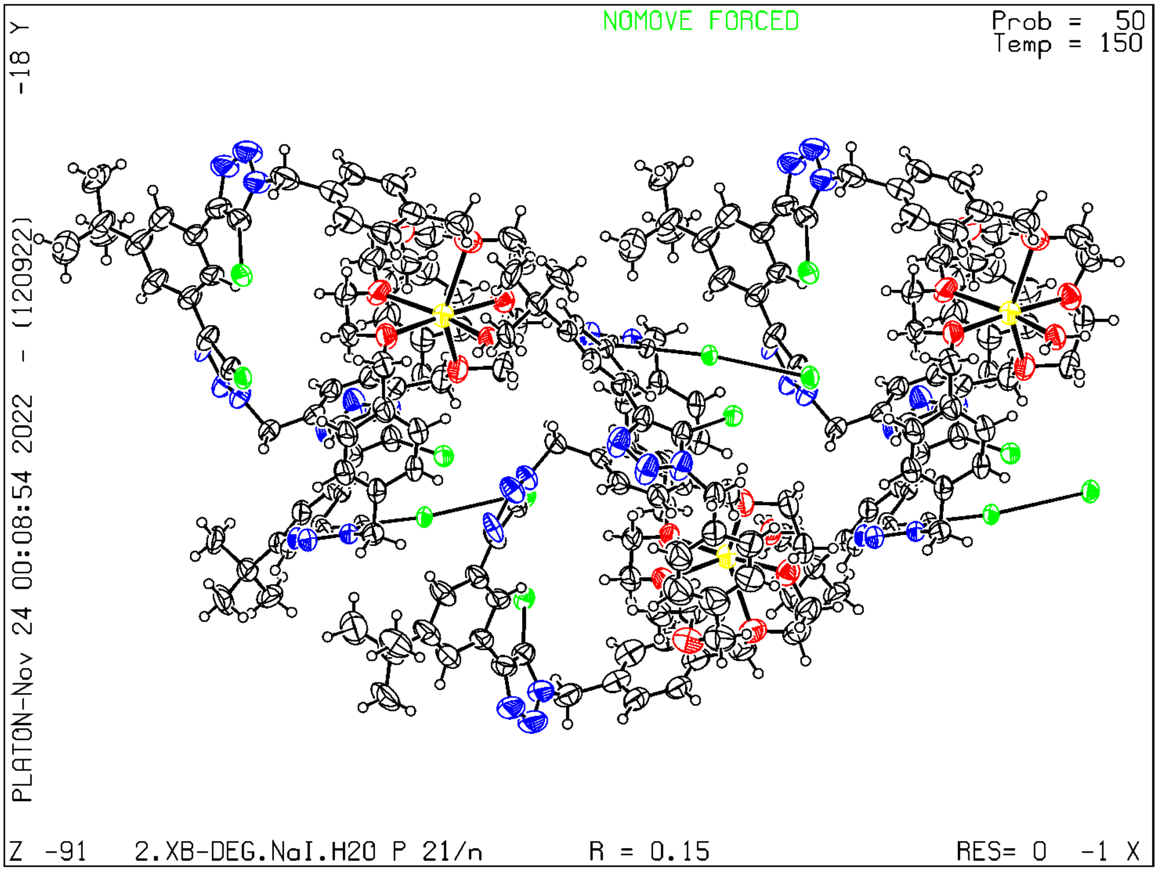

Datablock 2.XB-TEG.NaI - ellipsoid plot

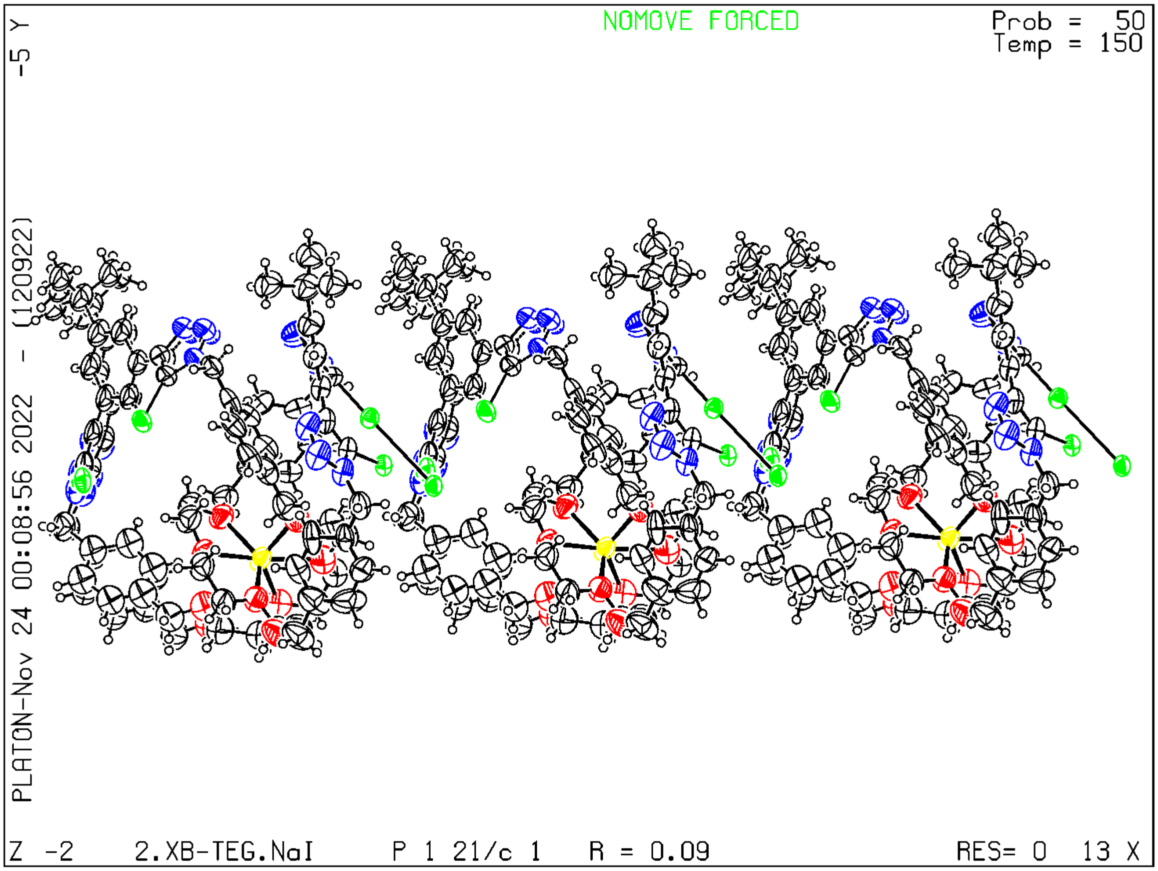

Datablock 2.HBXB-DEG - ellipsoid plot

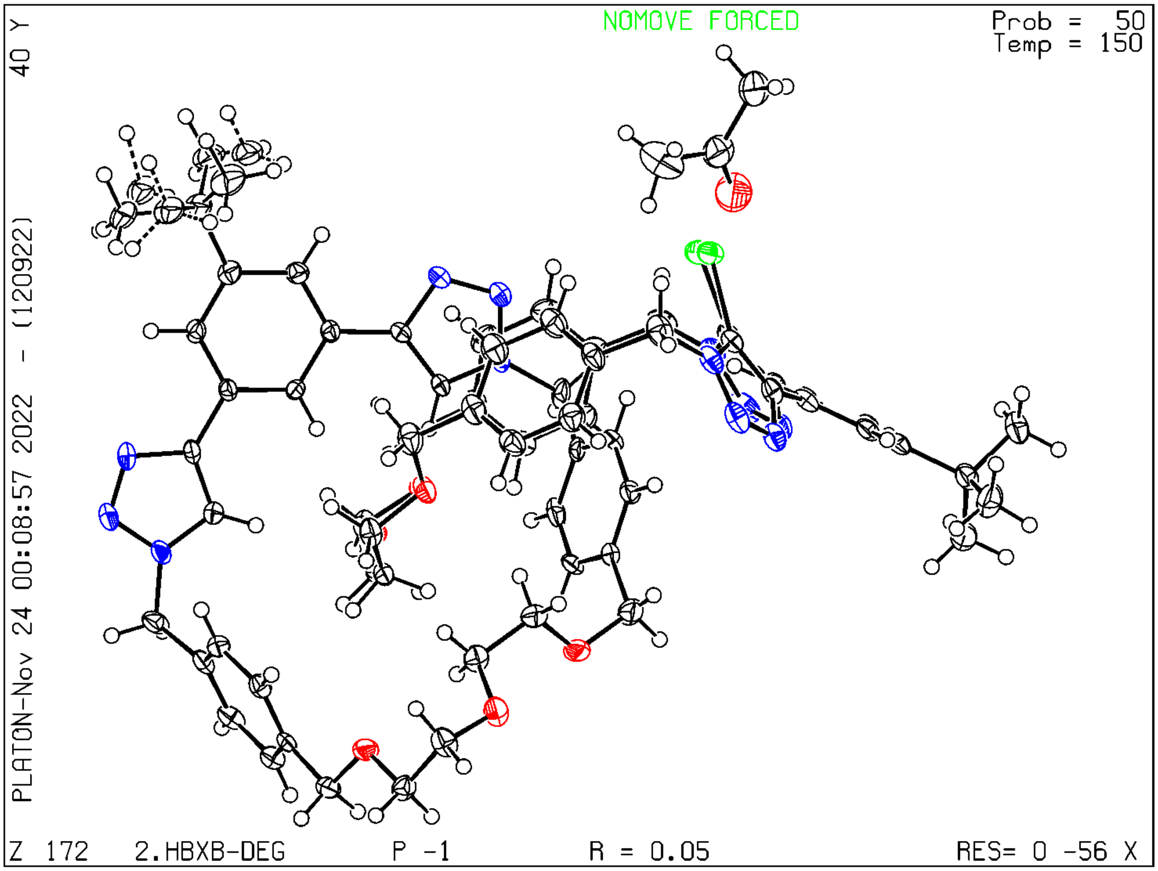

Datablock 2.XB-TEG - ellipsoid plot

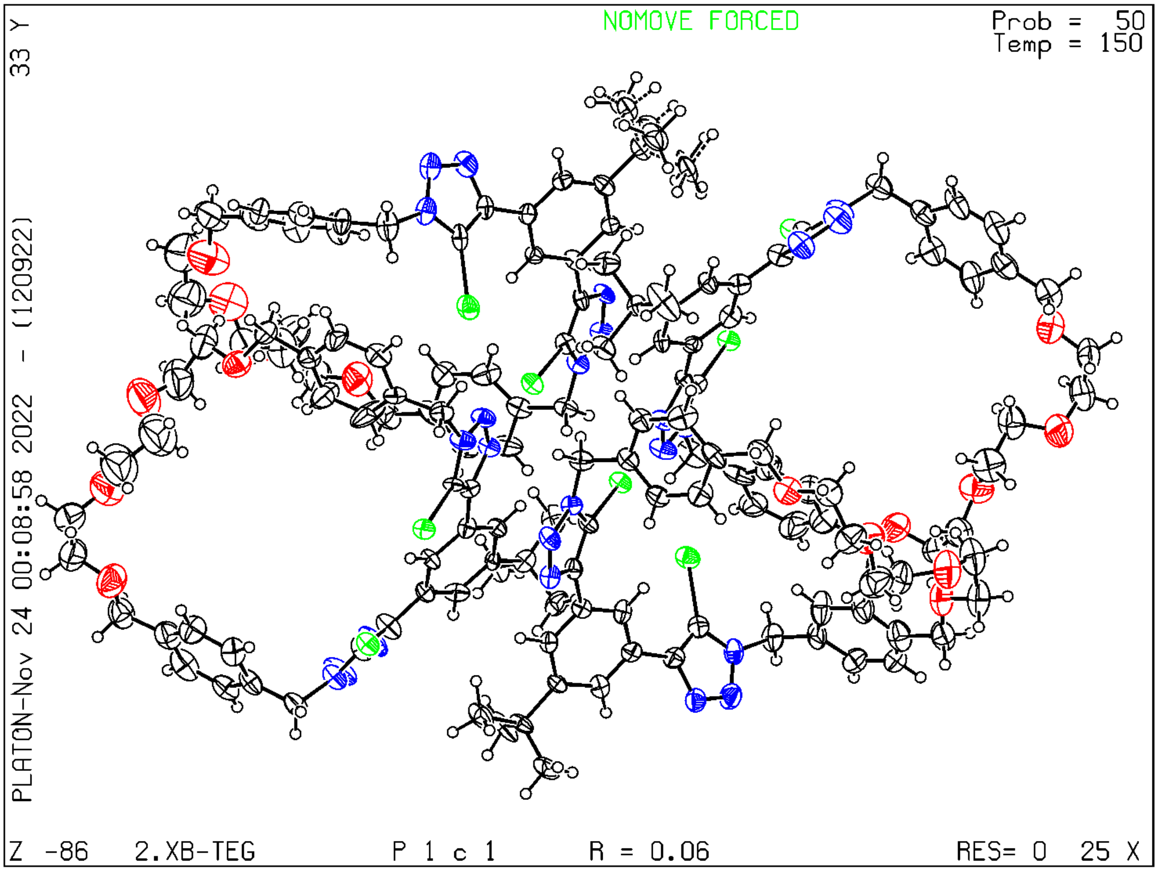

Supplement: Supplementary file 2 — Supporting Information [file ANIE-62-0-s001.pdf]
